# High-Throughput Screening of an FDA-Approved Compound Library Reveals a Novel GAS6 Receptor Agonist for Therapeutic Intervention in Septic Myocardial and microvascular Injury via Modulation of Danger-Associated Molecular Patterns

**DOI:** 10.7150/ijbs.104427

**Published:** 2024-11-11

**Authors:** Haowen Zhuang, Chun Li, Lingjun Wang, Bei Zhou, Zhijiang Guo, Yusheng Huang, Bo Deng, Yulin Ouyang, Junxiong Qiu, Xing Chang, Wei Wang, Junyan Wang

**Affiliations:** 1 State Key Laboratory of Traditional Chinese Medicine Syndrome, Guangzhou University of Chinese Medicine, Guangzhou, 510006, China.; 2 School of Pharmaceutical Sciences, Guangzhou University of Chinese Medicine, Guangzhou, Guangdong, 510006, China.; 3 Center for Drug Evaluation, National Medical Products Administration, Beijing, 510260, China.; 4 Guang'anmen Hospital, China Academy of Chinese Medical Sciences, Beijing, 100053, China; 5 The Second Affiliated Hospital of Guangzhou Medical University, Guangzhou, 510260, China.; 6 Xianning Medical College, Hubei University of Science & Technology, Xianning 437000, China.

**Keywords:** septic cardiomyopathy, PGAM5, VDAC1, mitophagy, mitochondria

## Abstract

PGAM5 and VDAC1 have both been reported to regulate mitophagy. However, the mechanisms by which they regulate sepsis-induced inflammatory microvascular injury remain unverified. In previous studies, we established the role of this regulatory axis in various phenotypic processes, including mitophagy, mitochondrial biogenesis, the mitochondrial unfolded protein response, and mitochondrial dynamics, while further confirming the interactive regulatory proteins within this axis. However, the validation and elucidation of these regulatory phenotypes have primarily focused on ischemic heart diseases such as ischemic myocardial injury and heart failure. Sepsis-related myocardial injury is currently recognized as a significant cardiac impairment, and although there are cardioprotective and nutritional agents available for supportive therapy, fundamental research validating the upstream targets and mechanisms of microvascular injury is still lacking. Based on our previous research, we further explored the role of mitophagy dysfunction mediated by VDAC1 and its upstream regulatory protein PGAM5 in sepsis-induced coronary microvascular injury. We also confirmed the material basis and metabolic pathway regulation targeting the PGAM5- VDAC1 interactive mechanism with relevant drugs. Our findings suggest that PGAM5-mediated mitophagy dysfunction may be a crucial factor leading to sepsis-induced microvascular injury, primarily interacting with VDAC1-mediated mitochondrial membrane dysfunction. Animal experiments revealed that cardiac-specific knockout of PGAM5 could reverse LPS-induced coronary microvascular injury and inflammatory damage, restoring cardiac ejection function and mitophagy functionality. *In vitro* studies also confirmed that the PGAM5-VDAC1 interaction can normalize mitophagy, restoring the normal morphology and structure of mitochondria while maintaining normal mitochondrial energy metabolism levels and respiratory chain function. Further pharmacological research indicated that the active ingredients of traditional Chinese medicine—Puerarin (TCM, a GAS6 Receptor Agonist) can target the PGAM5- VDAC1 axis to regulate mitophagy and inhibit LPS-induced necrotic apoptosis in cardiomyocytes, potentially reversing mitochondrial pathway-related cardiac injury. TCM may emerge as a prospective therapeutic agent targeting the PGAM5- VDAC1 axis.

## Introduction

Sepsis is a severe systemic infection that can lead to myocardial injury, clinically manifesting as cardiac dysfunction^1^. Due to myocardial damage, blood circulation may be obstructed, resulting in decreased blood pressure and potential circulatory failure^2^, which can cause significant fluid loss and further drops in blood pressure^3^, ^4^. In the most severe cases, shock or heart failure may occur, exacerbating myocardial injury and leading to heart failure symptoms such as shortness of breath, exercise intolerance, and cold extremities^5^, ^6^. Clinical studies have confirmed that approximately 50% of patients exhibit myocardial suppression in the early stages, accompanied by symptoms like hypotension and arrhythmias, with subsequent development of cardiac dysfunction and a mortality rate exceeding 70%. In recent years, the importance of coronary microvascular injury accompanying sepsis-related myocardial injury has gained attention^7^-^9^.

The primary pathological mechanisms of microvascular injury include oxidative stress damage, mitochondrial dysfunction, excessive pathological fission, impaired mitochondrial biogenesis, and mitochondrial energy metabolism dysfunction^10^-^13^. These mechanisms further lead to the activation of multiple programmed cell death pathways in cardiomyocytes, mediating damage to both cardiomyocytes and coronary microvascular endothelial cells^14^, ^15^. Additionally, dysregulation of mitophagy can result in mitochondrial pathway-dependent apoptosis, contributing to impaired cardiac ejection function and cardiomyocyte damage^16^-^18^. To counteract sepsis-induced myocardial and coronary microvascular endothelial injury, two primary repair mechanisms are usually engaged: the coordinated action of mitophagy alongside the unfolded protein response, and the regulation of mitochondrial dynamics, and the balance of mitochondrial dynamics^19^-^21^. These mechanisms facilitate normal mitochondrial biogenesis and restore endothelial self-repair functions in microvessels^22^-^25^. Our previous research described the molecular and biological basis for mitochondrial dysfunction in the progression of sepsis-related cardiac dysfunction^26^, ^27^. However, knowledge regarding the targeted regulatory effects on mitochondria and the associated drug mechanisms remains limited.

Mitophagy and dynamics are activated to sustain normal function following mitochondrial injury^28^, but imbalances can lead to several programmed cell death pathways^29^-^33^. Current studies suggest a connection between Phosphoglycerate Mutase 5 (PGAM5) and mitochondrial impairment^34^. When mitochondria experience mild damage, PGAM5 triggers mitochondrial biogenesis and mitophagy to enhance compensatory cellular responses. In contrast, significant mitochondrial damage results in excessive fission driven by PGAM5, causing disrupted dynamics and heightened signals for apoptosis, necrosis, and autophagic cell death, ultimately leading to the demise of cardiomyocytes or vascular endothelial cells^35^, ^36^. Autophagic death, ultimately mediating the death of cardiomyocytes or vascular endothelial cells. PGAM5 primarily exerts its effects through protein-protein interactions and specific Ser/Thr/His phosphatase activity and is regulated by mitochondrial proteases^37^.

Our prior research confirmed that PGAM5 can exacerbate cardiac ischemia-reperfusion injury by impairing mitochondrial quality control^38^. Additionally, PGAM5, a mitochondrial-localized serine/threonine phosphatase, has been identified as a novel necroptosis-inducing protein^39^. Relevant studies have shown that PGAM5 transcription and expression are significantly upregulated in reperfusion-induced myocardial injury^40^. Gene-specific knockout of PGAM5 inhibited the activation of necroptosis pathways mediated by ischemia-reperfusion (I/R), resulting in improved cardiac function and suppressed inflammatory responses. Experimental results further suggest that PGAM5 may serve as a target mediating LPS-related inflammatory (coronary microvascular endothelial) injury. In subcellular contexts, PGAM5 deficiency was associated with elevated mitochondrial DNA copy numbers and transcription levels, which helped normalize mitochondrial respiration, decrease mitochondrial ROS production, and inhibit the pathological opening of the mitochondrial permeability transition pore (mPTP) due to I/R. Further PGAM5 knockout blocked I/R-induced dephosphorylation of Drp1 at S637 but failed to inhibit phosphorylation of Drp1 at S616, which mediates the suppression of mitochondrial fission^40^.

Furthermore, PGAM5 acts as a protein phosphatase that mediates the dephosphorylation of Bax, promoting its translocation to the mitochondrial membrane. The translocation of Bax to the mitochondria increases mitochondrial membrane permeability, decreases mitochondrial membrane potential, and facilitates the release of cytochrome c (Cyt c) into the cytoplasm. Knockout of Bax attenuates the Cyt c release and apoptosis of renal tubular cells induced by PGAM5 overexpression. PGAM5-mediated dephosphorylation of Bax and its mitochondrial translocation are associated with the initiation of Cyt c release and the activation of mitochondrial-dependent apoptotic pathways, which contribute to the development of acute kidney injury (AKI)^41^.

Subsequent experimental findings reveal that knocking down Pgam5 preserves the viability of hepatocytes and mitigates ethanol-induced apoptosis, underscoring its significant role in intensifying hepatocyte dysfunction. Pgam5 mediates the oligomerization of VDAC1, which subsequently leads to the opening of the mitochondrial permeability transition pore (mPTP), mitochondrial swelling, and the initiation of apoptosis. Notably, both the silencing of Pgam5 and treatment with VBIT-12 markedly decrease VDAC1 oligomerization, thereby safeguarding mitochondrial function, stabilizing mitochondrial membrane potential, and further diminishing reactive oxygen species (ROS) levels^42^. Studies conducted *in vivo* with hepatocyte-specific Pgam5 knockout indicate that knocking out PGAM specifically alleviates ethanol-induced liver tissue damage, inflammation, lipid peroxidation, and metabolic imbalances, further confirming its role in the development of alcoholic liver disease (ALD). The experimental results also suggest that Pgam5 interacts with VDAC1, highlighting its important features for mitochondrial protection. VDAC1, a highly abundant channel protein located on the outer mitochondrial membrane, is a crucial regulator of mitochondrial function. In addition to coordinating the transport of metabolic products, cholesterol, and fatty acids across the mitochondrial membrane, VDAC1 is also involved in processes such as apoptosis of cardiomyocytes and endothelial cells, mitochondrial DNA release, and the regulation of mitochondrial oxidative stress^42^. Previous research has indicated that drugs capable of regulating mitophagy (Nature medicine) can maintain mitochondrial calcium homeostasis and normalize mitochondrial function in cardiomyocytes following hypoxic injury through the interaction of TMBIM6 and VDAC1^43^. In order to further confirm the mechanism of traditional Chinese medicine in treating inflammation mediated myocardial injury, we screened an effective myocardial/vascular protective drug through high-throughput sequencing: Puerarin (TCM).

We have confirmed in our previous research that puerarin, as a GAS6 receptor agonist, has vascular protection, anti-inflammatory, anti oxidative stress, and anti apoptotic effects. In the early stage, we also confirmed its regulatory effect on mitophagy in vascular endothelial protection through research, but its mechanism of action on SCM mediated myocardial and microvascular injury has not yet been explained. This mechanism contributes to the improvement of ischemic myocardial injury and provides myocardial protection. Additionally, these drugs stabilize β-tubulin, further enhancing mitochondrial energy metabolism and inhibiting necrotic apoptosis in sinoatrial node cells through mitochondrial pathways. Building on prior findings, this study aims to elucidate the role of PGAM5-VDAC1-mediated mitophagy dysfunction in septic microvascular injury and to further clarify the mechanisms of action of targeted therapeutic agents.

## Methods and Materials

### Animals and septic cardiomyopathy (SCM) model

Approval for all experimental procedures related to the treatment and surgery of the SCM animal model was granted by the Animal Care and Use Committees of the Guangzhou University of Traditional Chinese Medicine. Wild-type male mice (C57BL/6J, 8-10 weeks old) and transgenic experimental mice were provided by the Experimental Animal Center of Guangzhou University of Traditional Chinese Medicine, where they were bred and maintained under ABSL-2 conditions. Upon completion of the animal studies, mice were subjected to euthanasia under deep anesthesia with pentobarbital, and key organs (including the heart and kidneys) were harvested. Following our established protocols for SCM-related animal experiments, lipopolysaccharide (LPS, 20 mg/kg; Escherichia coli 0111, #2630, Sigma-Aldrich) was administered via intraperitoneal injection to induce the SCM mouse model for 48 hours. The control group received an equivalent volume of phosphate-buffered saline. Blood samples were collected and centrifuged (400 g for 20 minutes at 4°C) to separate the plasma. Cardiac tissue was quickly excised and stored at -80°C for further pathological staining analysis^26^.

### ELISA

To confirm the biomarkers of myocardial and microvascular injury, serum levels of creatine kinase MB (CK-MB, #E4607, BioVision), troponin T (TNT, #MBS2104865, MyBioSource, Inc.), and lactate dehydrogenase (LDH, #MS720560, MyBioSource, Inc.) were measured using colorimetric/fluorescent assay kits. In accordance with the manufacturer's protocol, the activity of caspase-3, caspase-9, and caspase-12 was evaluated using ELISA.

### Seahorse assay

To achieve 95% confluence, microvascular endothelial cells were cultured in XF24 cell culture microplates (Cat. #100777-004; Agilent; Santa Clara, CA, USA) at a density of 4 × 10^4^ cells per well. Real-time measurements of mitochondrial respiration rates were conducted using the XF-24 Extracellular Flux Analyzer (Agilent 103592-100; Seahorse Bioscience; Santa Clara, CA, USA). The culture medium was replaced with unbuffered XF medium (XF DMEM, pH 7.4, Catalog #103757-100; Agilent) and the coronary microvascular endothelial cells were incubated for 1 hour at 37°C without CO₂. The XF24 sensor cartridge was hydrated overnight in calibration buffer, followed by the addition of relevant compounds: 2.5 μg/mL oligomycin, 2 μM rotenone, and 4 μM antimycin A to stimulate mitochondrial respiratory activity. Data were processed using the Agilent ATP measurement report generator and analyzed statistically with Prism 9.0 software (GraphPad)^44^.

### Cell culture

Microvascular endothelial cells were obtained from the Experimental Animal Center of Guangzhou University of Traditional Chinese Medicine. The cells were cultured in Dulbecco's Modified Eagle Medium (DMEM) containing glutamine in a 37°C incubator with 5% CO₂. The medium was supplemented with 10% FBS and 100 μg/mL penicillin/streptomycin. To induce SCM (LPS-mediated inflammatory cell injury) *in vitro*, the cells were treated with 10 μg/mL LPS for 24 hours^45^,^46^.

### Immunofluorescence staining

Microvascular endothelial cells were cultured on slides for 48 hours, initially in medium containing 10% FBS for 24 hours, followed by 24 hours in serum-free medium. The cultured microvascular endothelial cells were pre-treated with immunofluorescent probes targeting Tom20 (1:500; #ab78547, Abcam) and lysosomal markers. Cardiac tissue sections were fixed in 4% paraformaldehyde (PFA), embedded in paraffin, and sectioned. The sections were permeabilized with 0.1% saponin and incubated with Gr1 (1:500; #ab25377, Abcam) and TnT (1:500; #MBS533262, MyBioSource, Inc.) antibodies. Cell nuclei were stained with DAPI. The stained slides were examined using a Zeiss Axio Observer microscope equipped with an Apotome attachment.

### Real-time quantitative PCR

Total RNA extraction from 10 mg of microvascular/cardiac tissue was performed using TRIzol reagent (Invitrogen), followed by purification with chloroform and precipitation using isopropanol. RNA concentration was determined with a NanoDrop instrument (Thermo Fisher Scientific).The PrimeScript™ RT reagent kit was used for reverse transcription of RNA, and cDNA was amplified using a fast real-time PCR instrument equipped with TB Green Premix Ex Taq™ II (RR820A, Takara, Japan) (ABI-7900-384, Applied Biosystems). Gene amplification was performed by qPCR. The reaction was initiated at 94°C for 10 minutes, followed by 40 cycles of 94°C for 10 seconds, 60°C for 30 seconds, and 94°C for 10 seconds. All reactions were conducted in duplicate. Amplification curves were analyzed using SDS 1.9.1 software (Applied Biosystems), and the relative mtDNA ratio for each sample was determined based on these curves.

### Construction of Adenoviral Vectors and Plasmid Infection

PGAM5 and VDAC1 expression plasmids were created by inserting the open reading frame of each cDNA into the multiple cloning sites of the pcDNA3.0 vector, which includes HA or Myc tag sequences. Recombinant adenoviral vectors encoding mouse PGAM5 and VDAC1 genes were generated and transfected into HEK293 cells to produce adenovirus for the overexpression of PGAM5 and VDAC1 in endothelial cells. The adenovirus was produced using the AdEasy adenoviral vector system kit (240009, Agilent Technologies)^45^.

### Statistical Analysis

Data are expressed as means ± standard error of the mean (SEM). Comparisons between two groups were made using either the parametric Student's t-test or the non-parametric Mann-Whitney test. For comparisons involving two or more groups, parametric one-way analysis of variance (ANOVA) was performed, followed by Bonferroni post hoc tests. Statistical analysis of histopathological parameters was conducted using the chi-square test. Results were analyzed using GraphPad Prism 9.0 statistical software, with a significance threshold set at P < 0.05.

## Results

### Abnormal activation of PGAM5-mediated mito-UPR as an inductive mechanism for septic coronary microvascular injury

**Figure 1:**
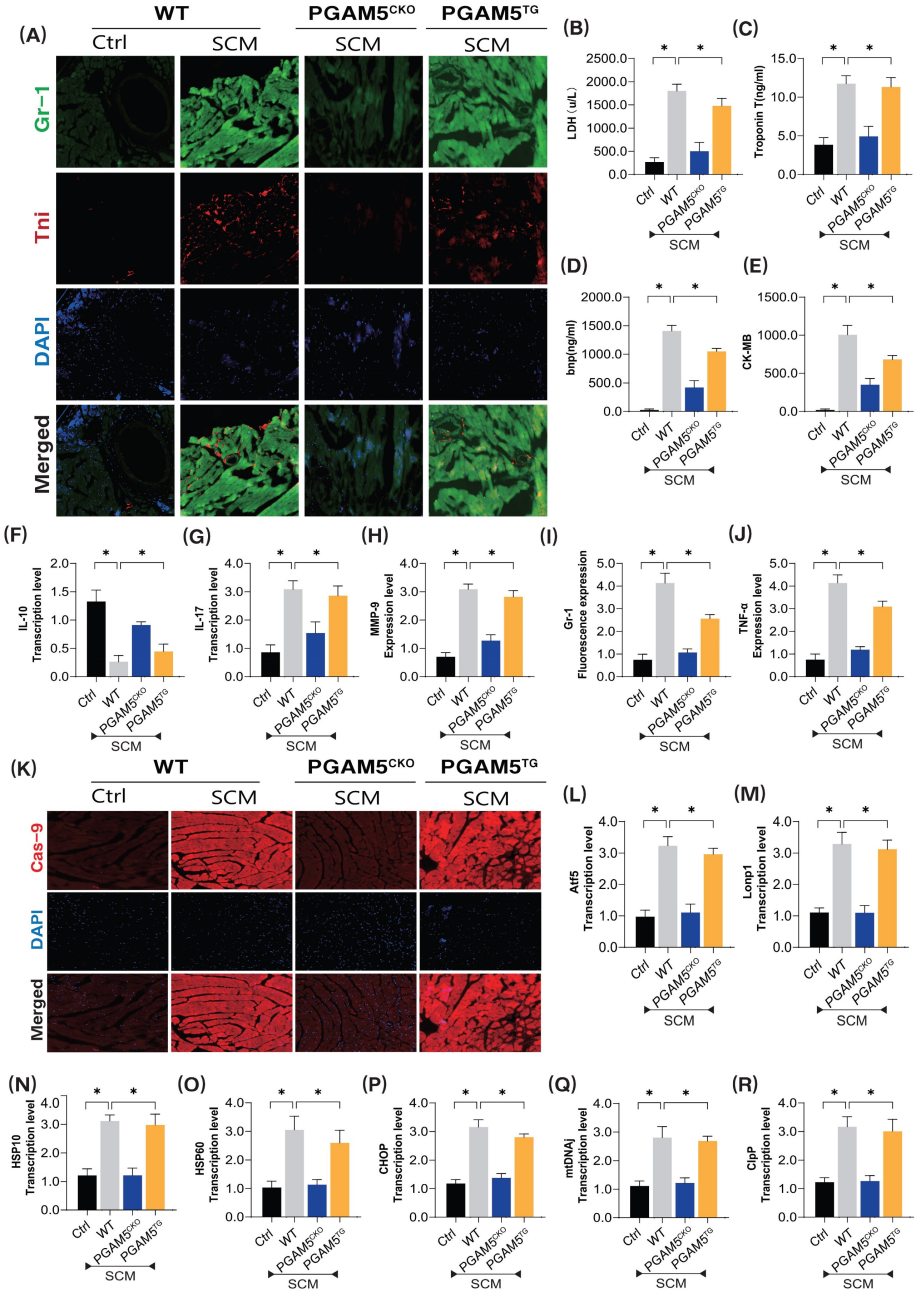
** The pathological mechanism of PGAM5 mediated mitochondrial unfolded protein response involved in microvascular injury in sepsis. (A)** Immunofluorescence detection of markers of sepsis mediated coronary microvascular inflammation and injury; **(B)** The expression level of LDH; **(C)** The expression level of myocardial injury marker (Tnt); **(D)** The expression level of Bnp; **(E)** The expression level of CK-MB; **(F)** The expression level of anti-inflammatory factor IL-10; **(G)** The expression level of inflammatory cytokine IL-17; **(H)** The expression level of inflammatory cytokine MMP-9; **(I)** Fluorescence expression level of Gr-1; **(J)** The expression level of TNF-α; **(K)** The expression level of Caspase-9, a marker of mitochondrial damage pathway; **(L-R)**Transcriptional levels of mito-UPR regulated genes (ATF5/Lonp1/HSP10/HSP60/CHOP/mtDNAj/ClpP; Experiments were repeated at least three times and the data are shown as mean ± SEM (N = 12 mice per group). *p < 0.05.

To further validate the pathological mechanism of PGAM5 in septic coronary microvascular injury, we established a model of septic myocardial injury and examined the pathological mechanisms and target sites of microvascular damage using immunofluorescence dual staining and specific biomarker detection methods (Figure 1A-K). Additionally, we developed PGAM5 gene-modified (cardiac-specific knockout/transgenic) mouse models based on WT wild-type mice (Figure 1A-K).

The experimental results demonstrated significant overexpression of inflammatory damage markers in the coronary microvasculature of mice after modeling septic cardiomyopathy (SCM) (Figure 1A-K). There was also a notable increase in myocardial injury markers such as LDH, Trop T, BNP, and CK-MB, along with significant elevation of pro-inflammatory cytokines and downregulation of anti-inflammatory factors (Figure 1A-K). Notably, the specific knockout of PGAM5 significantly reversed these pathological changes, reducing levels of microvascular inflammatory damage and lowering the expression of inflammatory and myocardial injury markers while increasing anti-inflammatory factor levels (Figure 1A-K). Interestingly, PGAM5 transgenic treatment did not reverse these pathological changes, as this group of mice still exhibited inflammatory damage and pathological alterations in the coronary microvasculature (Figure 1A-K).

To further elucidate the phenotypic mechanisms and pathological changes of PGAM5-mediated coronary microvascular inflammatory damage, we performed tissue fluorescence staining to detect Caspase-9 expression surrounding the coronary microvasculature and further assessed the mRNA expression levels of genes related to the mitochondrial unfolded protein response (Figure 1K-R). The results indicated significant overexpression of Caspase-9 in the perivascular tissues following SCM, accompanied by abnormal activation of genes associated with the mitochondrial unfolded protein response. Importantly, the specific knockout of PGAM5 reversed these phenomena, leading to reduced expression of Caspase-9 and lower mRNA levels of mitochondrial unfolded protein response-related genes (Figure 1K-R). However, PGAM5 transgenic treatment did not reverse these pathological responses. (Figure 1K-R) This further confirms that the dysregulation of protein homeostasis within the mitochondrial pathway is an important phenotype of mitochondrial pathway-mediated cell death, with PGAM5 potentially serving as a critical protein mediating these pathological changes.

### PGAM5-mediated impairment of mitophagy as an inductive mechanism for SCM-induced damage in coronary microvascular mitochondrial pathways

In previous studies, we confirmed that mitophagy and the mitochondrial unfolded protein response exert significant synergistic effects in maintaining mitochondrial and intracellular homeostasis (including calcium stability, redox balance, and the integrity of mitochondrial morphology) during hypoxia or ischemia-induced myocardial injury. Building on these prior experiments, we hypothesized that PGAM5-mediated dysfunction of the mitochondrial unfolded protein response may further contribute to microvascular injury through mitochondrial pathways.

**Figure 2:**
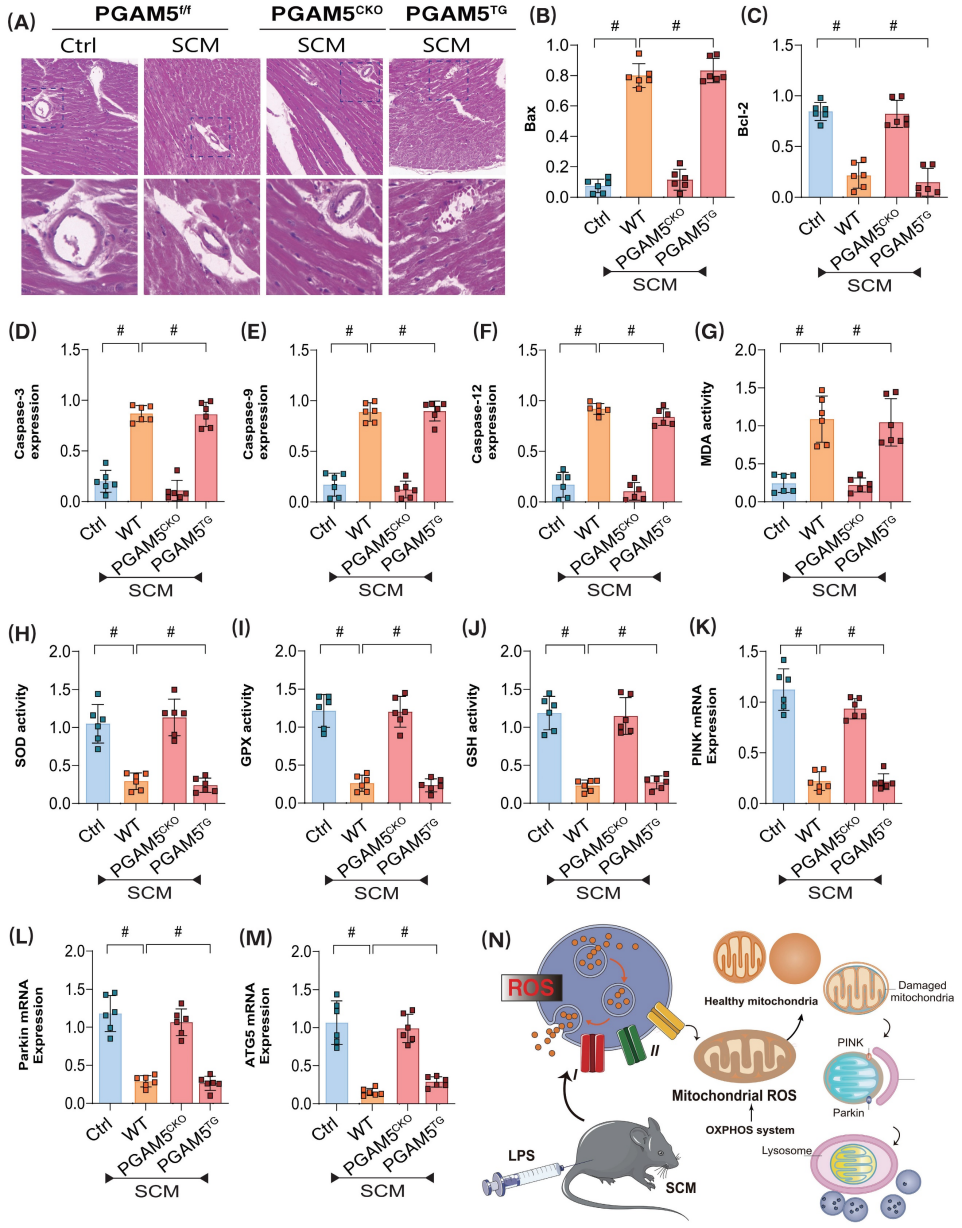
** PGAM5 mediates mitophagy and programmed cell death in the pathological mechanism of microvascular injury in sepsis. (A)** Immunofluorescence detection of coronary microvascular injury;** (B)** The expression level of apoptosis factor Bax; **(C)** The expression level of anti-apoptotic factor Bcl-2; **(D-F)** Expression levels of Caspase-3, Caspase-9, and Caspase-12; **(G)** MDA activity detection;(H) SOD activity detection; **(I)** GPX activity detection; **(J)** GSH activity detection; **(K)** The transcription level of PINK; **(L)** The transcription level of Parkin; **(M)** The transcription level of ATG5; Experiments were repeated at least three times and the data are shown as mean ± SEM (N = 12 mice per group). *p < 0.05.

Coincidentally, further experiments revealed significant morphological and structural abnormalities in the coronary microvasculature of mice post-modeling. However, cardiac-specific knockout of PGAM5 did not improve the pathological changes in microvasculature. To further elucidate the pathological mechanisms of PGAM5-mediated coronary microvascular injury through mitochondrial pathways, we evaluated the expression levels of apoptosis-related markers, including Bax/Bcl-2, Caspase-3, Caspase-9, and Caspase-12. The experimental results indicated a significant increase in Bax expression and a decrease in Bcl-2 levels in the pathological tissues of modeled mice; this was accompanied by elevated expression of Caspase-3, -9, and -12 (Figure 2A-F). Additional analyses of oxidative stress damage and genes related to mitophagy suggested that post-modeling inflammatory damage was associated with overexpression of oxidative stress markers and reduced activity of antioxidant enzymes, alongside decreased expression levels of the mitophagy-related genes PINK and Parkin, as well as a reduction in the autophagy marker gene ATG5(Figure 2G-N). These findings further suggest that PGAM5-mediated dysfunction of the synergistic relationship between mitophagy and the mitochondrial unfolded protein response may represent a critical pathological mechanism underlying coronary microvascular injury following SCM, closely linked to apoptosis and oxidative stress damage within mitochondrial pathways.

### PGAM5-mediated mitochondrial homeostasis disruption as a target for LPS-mediated apoptosis of coronary microvascular endothelial cells

**Figure 3:**
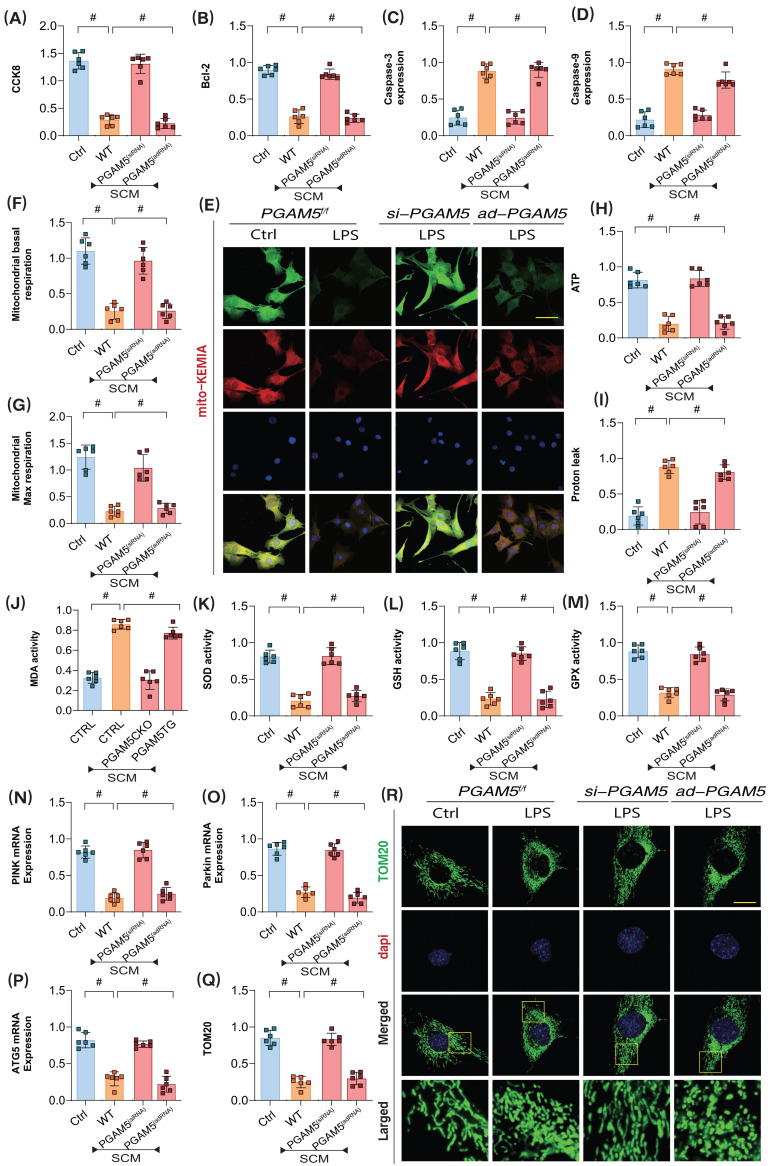
** PGAM5 mediated mitophagy and energy metabolism dysfunction participate in the pathological mechanism of microvascular endothelial cell injury in sepsis. (A)** CCK8 detection of endothelial cell activity in coronary microvascular endothelial cells; **(B)** Bcl-2 activity detection; **(C)** The expression level of Caspase-3; **(D)** The expression level of Caspase-9; **(E)** Laser confocal detection of mitophagy markers; **(F)** Mitochondrial basal respiration detection; **(G)** Maximum mitochondrial respiration detection; **(H)** The level of ATP generation; **(I)** Proton leakage level detection; **(J)** MDA activity detection; **(K)** SOD activity detection; **(L)** GSH activity detection; **(M)** GPX activity detection; **(N)** The transcription level of PINK; **(O)** The transcription level of Parkin; **(P)** Transcriptional level detection of ATG5; **(Q-R)** Fluorescence expression level of mitochondrial lysosomes; Experiments were repeated at least three times and the data are shown as mean ± SEM (N = 12 mice per group). *p < 0.05.

To further confirm the pathological mechanisms of PGAM5-mediated coronary microvascular endothelial injury during the later stages of SCM, we employed lentiviral transfection techniques to achieve knockdown and overexpression of PGAM5 in coronary microvascular endothelial cells (using si-RNA/ad-RNA). Additionally, we intervened with LPS to establish a model of SCM-induced injury in these endothelial cells. CCK8 assay results showed that LPS intervention led to a significant decrease in endothelial cell viability; however, PGAM5 si-RNA treatment reversed this effect, enhancing the viability of endothelial cells post-LPS treatment. In contrast, PGAM5 ad-RNA treatment did not affect the pathological changes in these endothelial cells (Figure 3A).

To further elucidate the pathways by which PGAM5 regulates endothelial cell damage following LPS exposure, we assessed the activity of apoptosis markers and anti-apoptosis marker Bcl-2, Caspase-3, and Caspase-9. The results indicated significant overexpression of Caspase-3, and Caspase-9 following LPS treatment; however, PGAM5 si-RNA treatment reversed the expression levels of these markers. Further PGAM5 ad-RNA treatment did not alter these pathological changes in the endothelial cells (Figure 3B-D).

Additionally, experiments assessing cellular oxidative stress and mitochondrial energy metabolism confirmed that LPS intervention resulted in significant reductions in basal respiration, reserve respiration, and ATP production levels, along with increased proton leak levels (Figure 3E-M). Oxidative stress assays demonstrated a significant elevation of MDA following inflammatory model injury, coupled with a marked decrease in the activities of antioxidant enzymes such as SOD, GPX, and GSH, alongside suppression of mitophagy levels (Figure 3E-M). These results suggest that LPS-mediated inflammatory mediators may induce mitochondrial energy metabolism dysfunction and redox imbalance, which could be important inductive factors for apoptosis via mitochondrial pathways. PGAM5 si-RNA treatment reversed these phenomena, enhancing mitochondrial energy metabolism levels, boosting antioxidant enzyme activity, and inhibiting proton leak and oxidative stress damage. Conversely, PGAM5 ad-RNA treatment did not impact mitochondrial energy metabolism or oxidative stress (Figure 3E-M).

Further laser confocal microscopy and mt-Keima assays confirmed that LPS intervention resulted in decreased fluorescence expression levels of mitophagy lysosomes and low expression of mitophagy markers, along with reduced transcription levels of PINK, Parkin, and ATG5 (Figure 3 E and N-R). Following PGAM5 si-RNA treatment, the levels of mitophagy in cells increased. However, PGAM5 ad-RNA treatment did not affect mitophagy levels (Figure 3 E and N-R). These experimental findings further confirm that LPS intervention may lead to mitochondrial oxidative stress and insufficient energy metabolism, ultimately resulting in PINK- and Parkin-mediated mitophagy dysfunction.

### VDAC1 overexpression impairs mitophagy function in the PINK/Parkin pathway, preceding LPS-mediated apoptosis of coronary microvascular endothelial cells

**Figure 4:**
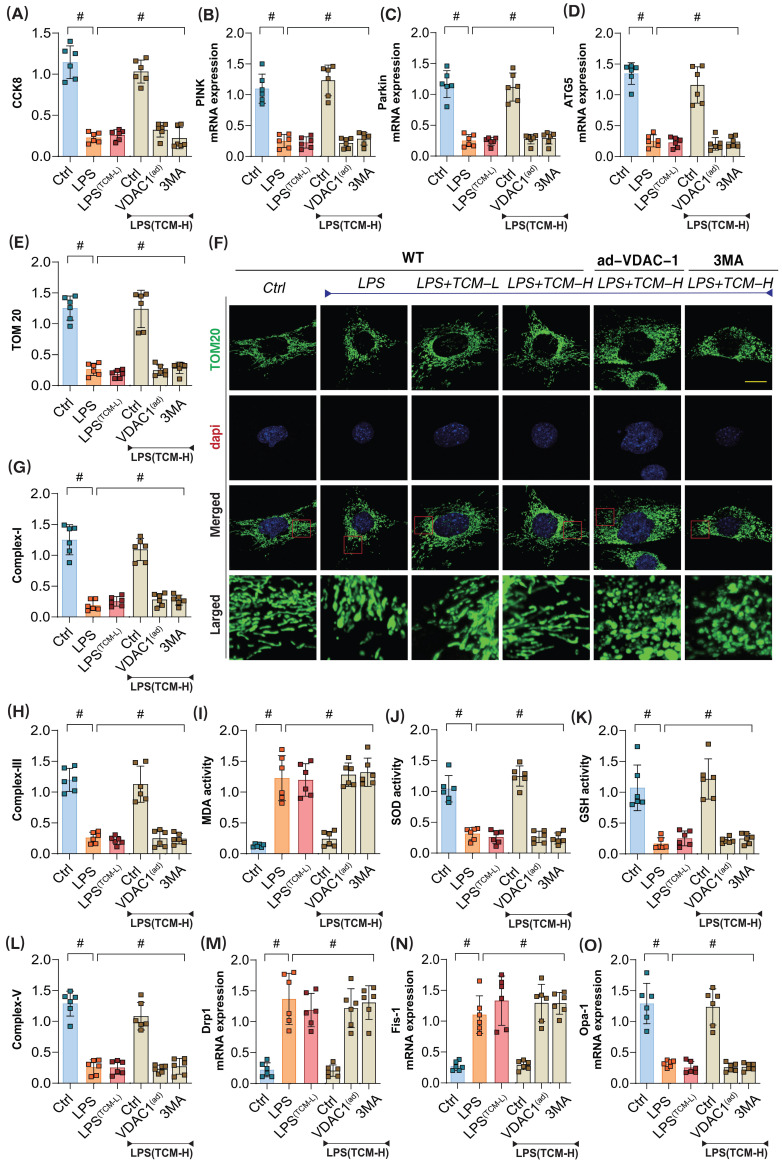
** The pathological mechanism and drug intervention mechanism of VDAC1 mediated mitochondrial homeostasis imbalance involved in sepsis microvascular endothelial cell injury. (A)** CCK8 detection of endothelial cell activity in coronary microvascular endothelial cells; **(B)** The transcription level of PINK; **(C)** The transcription level of Parkin; **(D)** Transcriptional level detection of ATG5; **(E-F)** Fluorescence expression level of mitochondrial lysosomes; **(G)** Mitochondrial respiratory chain complex (Complex-I) activity; **(H)** Mitochondrial respiratory chain complex (Complex III) activity;**(I)** MDA activity; **(J)** SOD activity; **(K)** GSH activity; **(L)** Mitochondrial respiratory chain complex (Complex-V) activity; **(M)** The transcription level of Drp1; **(N)** The transcription level of Fis1; **(O)** The transcription level of Opa1;Experiments were repeated at least three times and the data are shown as mean ± SEM (N = 12 mice per group). *p < 0.05.

We elucidated the interaction mechanisms between the drug and the proteins, demonstrating the high binding affinity of traditional Chinese medicine (TCM-Puerarin) with PGAM5 and VDAC1. To investigate the role of TCM in LPS-mediated inflammatory damage to microvascular endothelial cells, we applied both low-dose and high-dose TCM gradients to intervene in the injured endothelial cells. The results showed that the low-dose gradient did not reverse LPS-mediated inflammatory damage, while the high-dose TCM significantly enhanced cell viability after LPS injury (Figure 4A-O). Interestingly, the mitophagy inhibitor 3MA and VDAC1 ad-RNA treatment abolished the protective effects of TCM on endothelial cell damage, suggesting that VDAC1-mediated mitophagy dysfunction may play a crucial role in endothelial cell inflammatory injury (Figure 4A-O).

Related assessments confirmed that high-dose TCM could significantly reverse the low expression of PINK, Parkin, and ATG5 following LPS-induced inflammatory damage. Concurrently, mitochondrial respiratory chain complexes also exhibited significant downregulation after LPS modeling (Figure 4A-O); however, high-dose TCM increased their expression levels, further modulating the balance of oxidative stress markers and antioxidant enzyme activities (Figure 4A-O). Additional examinations of mitochondrial dynamics-regulating genes Drp-1, Fis1, and Opa1 revealed that LPS modeling significantly elevated the transcription levels of Drp1 and Fis1 while reducing Opa1 transcription levels (Figure 4A-O). High-dose TCM reversed these changes, whereas low-dose TCM did not exhibit any regulatory effects on mitochondrial dynamics. Notably, low-dose TCM did not influence mitophagy or dynamics, nor did it affect oxidative stress or mitochondrial respiratory chain complexes. However, the application of the mitophagy inhibitor 3MA and VDAC1 ad-RNA treatment eliminated the regulatory effects of TCM on mitophagy and dynamics (Figure 4A-O).

These results indicate that high-dose TCM treatment can restore mitophagy levels and normalize the function of mitochondrial respiratory chain complexes, while simultaneously mitigating oxidative stress damage in endothelial cells via mitochondrial pathways. VDAC1, as a mitochondrial anion channel, may be a key regulatory protein mediating endothelial mitochondrial damage post-LPS. TCM might regulate mitophagy and respiratory chain function by inhibiting VDAC1 activation, representing a crucial pathway through which TCM modulates endothelial cell energy metabolism and homeostasis.

### PGAM5 leads to dysregulation of mitophagy and unfolded protein response synergy in LPS-mediated coronary microvascular endothelial cells

**Figure 5:**
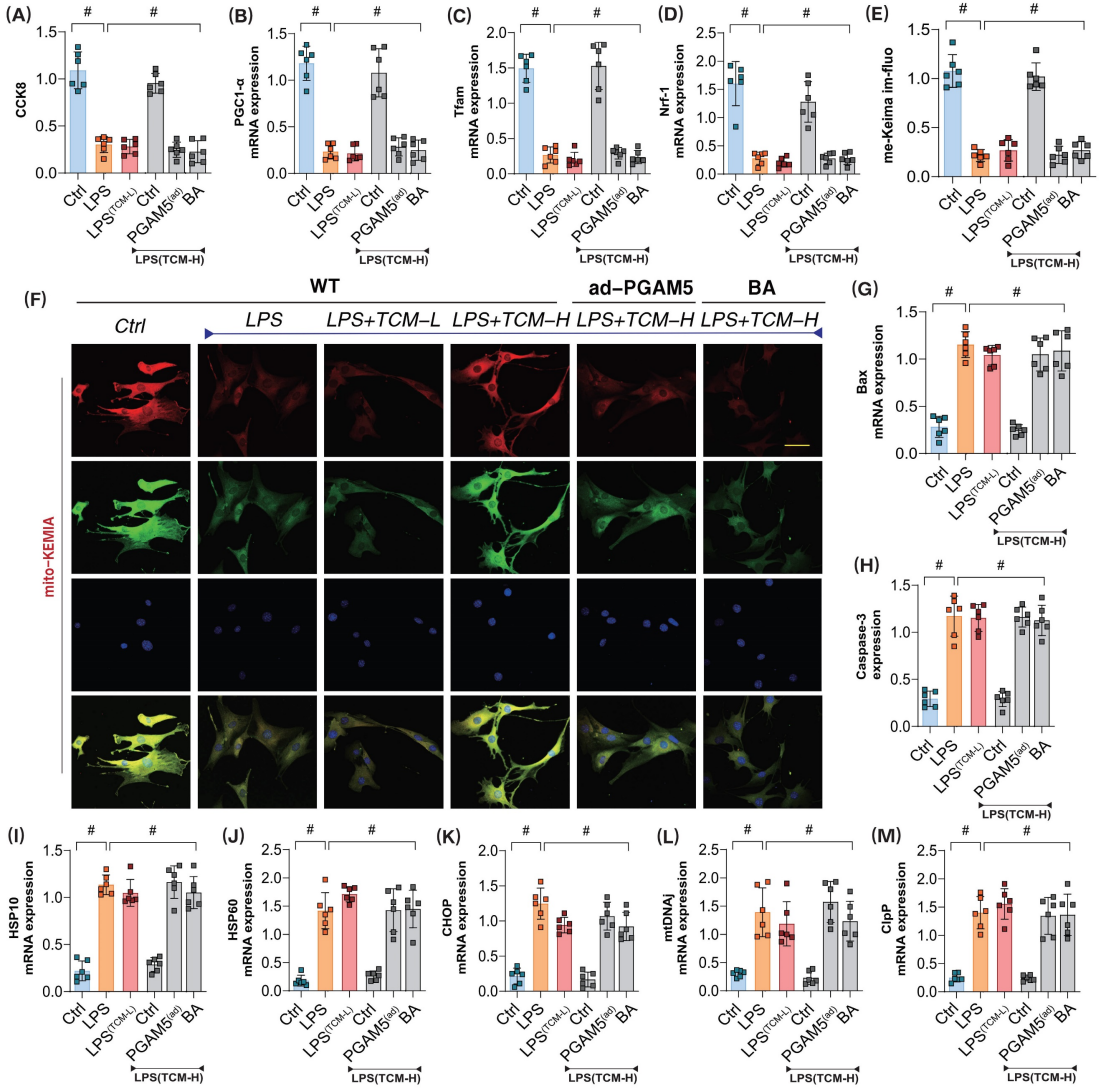
** The mitochondrial dysfunction mediated by PGAM5 is involved in the pathological mechanism and drug intervention mechanism of microvascular endothelial cell injury in sepsis. (A)** CCK8 detection of endothelial cell activity in coronary microvascular endothelial cells; **(B)** The transcription level of Pgc1-α; **(C)** The transcription level of Tfam; **(D)** Transcriptional level detection of Nrf-1; **(E-F)** Fluorescence expression level of mitophagy(mt-Keima); **(G)** Bax activity; **(H)** Caspase-3 activity; **(I-M)**; Transcriptional levels of mito-UPR regulated genes (HSP10/HSP60/CHOP/mtDNAj/ClpP); Experiments were repeated at least three times and the data are shown as mean ± SEM (N = 12 mice per group). *p < 0.05.

To further verify whether PGAM5 is the targeted protein through which TCM improves endothelial cell damage in the mitochondrial pathway after LPS exposure, we assessed microvascular endothelial cell viability using the CCK8 assay. Additionally, we examined mitochondrial biogenesis and the expression of genes related to mitophagy and the unfolded protein response to evaluate TCM's regulatory effects on the associated phenotypes of endothelial damage. The results indicated that significant overactivation of the unfolded protein response occurred in mitochondria following modeling, as evidenced by elevated mRNA levels of related regulatory genes. The low-dose gradient did not reverse LPS-mediated impairments in mitochondrial biogenesis or mitophagy and failed to further affect the overactivation of the unfolded protein response (Figure 5A-M). Further assessment of Caspase-3 and Bax expression confirmed a notable activation of apoptosis in conjunction with reduced mitophagy and biogenesis, as well as excessive unfolded protein response (Figure 5A-M). However, high-dose TCM could reverse these effects, enhancing mitophagy and biogenesis while suppressing the overactivation of the unfolded protein response and reducing Caspase-3 and Bax levels. Notably, PGAM5 ad-RNA treatment abolished the regulatory effects of high-dose TCM on these mechanisms. Additionally, the mitochondrial energy metabolism inhibitor BA blocked the therapeutic effects of TCM (Figure 5A-M). These results suggest that TCM may regulate the mitophagy-unfolded protein response through PGAM5, thereby influencing mitochondrial biogenesis and energy metabolism functions and activating apoptosis via mitochondrial pathways.

### TCM regulates mitophagy through the PGAM5-VDAC1 interaction mechanism to improve coronary microvascular injury

Previous experimental results confirm a strong interaction between PGAM5 and VDAC1. To further investigate whether TCM regulates apoptosis in endothelial cells via the PGAM5-VDAC1 interaction mechanism following LPS exposure, we established an LPS inflammation model in PGAM5 transgenic mice. Additionally, we created si-RNA and ad-RNA cell models targeting VDAC1. We then examined the effects of TCM on microvascular endothelial cell damage by assessing mitophagy, mito-biogenesis, mito-dynamics and the active of mitochondrial respiratory chain complexes.

**Figure 6:**
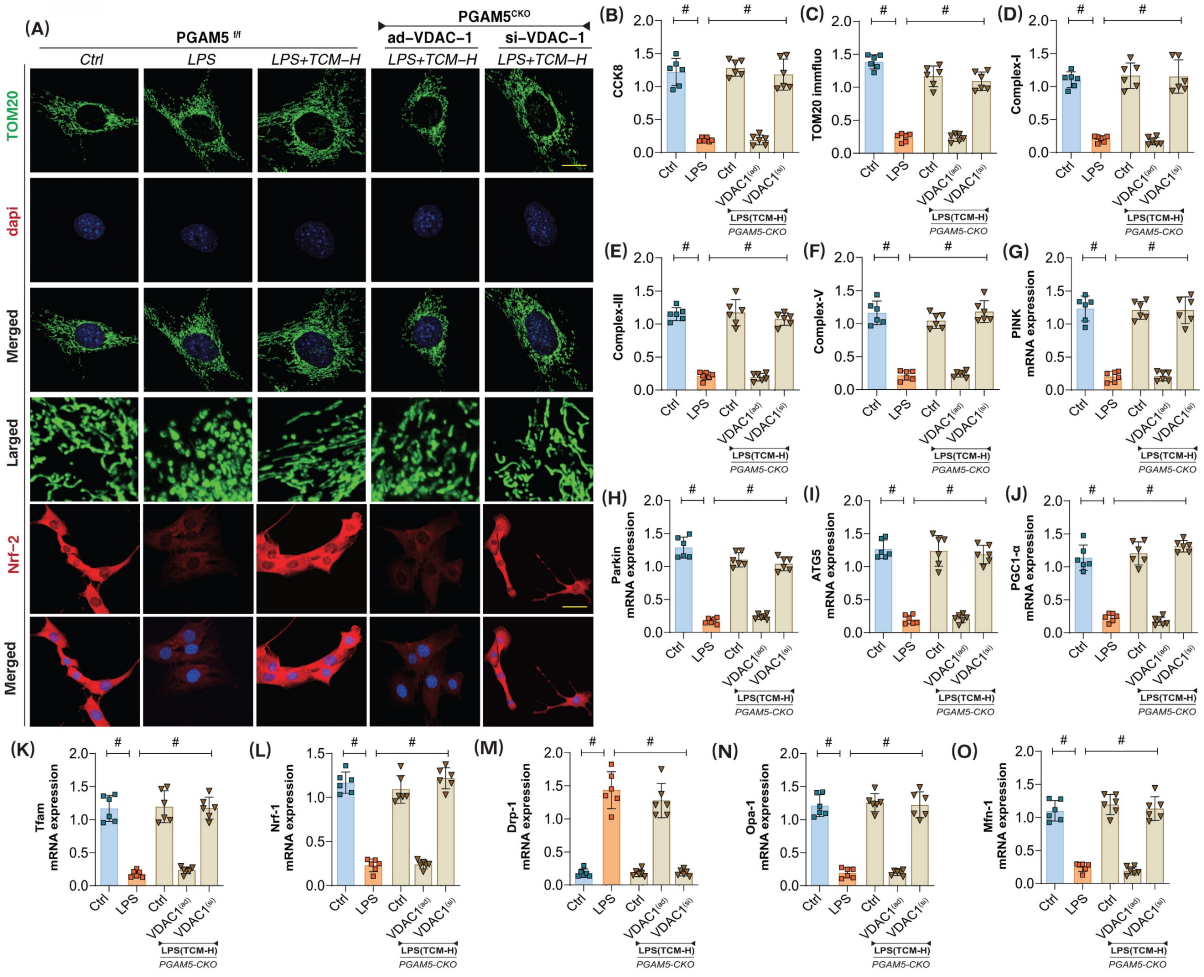
** The interaction mechanism of PGAM5-VDAC1 is involved in sepsis microvascular injury and drug action mechanism. (A,C)** Fluorescence expression level of mitochondrial lysosomes; **(B)** CCK8 detection of endothelial cell activity in coronary microvascular endothelial cells;** (D)** Mitochondrial respiratory chain complex (Complex-I) activity;** (E-F)** Mitochondrial respiratory chain complex (Complex-III/V) activity;** (G)** The transcription level of PINK; **(H)** The transcription level of Parkin;**(I)**; The transcription level of ATG5;** (J)** The transcription level of PGC1-α; **(K)** The transcription level of Tfam; **(L)** The transcription level of Nrf-1; **(M)** The transcription level of Drp-1; **(N)** The transcription level of Opa-1; **(O)** The transcription level of Mfn-1; Experiments were repeated at least three times and the data are shown as mean ± SEM (N = 12 mice per group). *p < 0.05.

The results indicated that the cardiac-specific knockout of PGAM5 and si-VDAC1 treatment did not affect TCM's ability to activate mitophagy or biogenesis, nor did they influence mitochondrial dynamics or the regulation of respiratory chain complexes (Figure 6A-O). Further experiments revealed that PGAM5 knockout and ad-VDAC1 treatment abolished TCM's regulatory effects on mitophagy and biogenesis. There was a significant increase in the mRNA expression of mitochondrial fission proteins (Drp1 and Fis1), while the fusion proteins Mfn/Opa1 were not further activated (Figure 6A-O). These findings suggest that TCM may modulate VDAC1 through PGAM5, thereby impacting the activation of PINK/Parkin-mediated mitophagy, inhibiting mitochondrial fission, and enhancing mitochondrial biogenesis and the expression of respiratory chain complexes. The interaction mechanism of PGAM5-VDAC1 may be crucial for TCM's regulation of mitochondrial homeostasis in endothelial cells after LPS injury.

**Figure 7:**
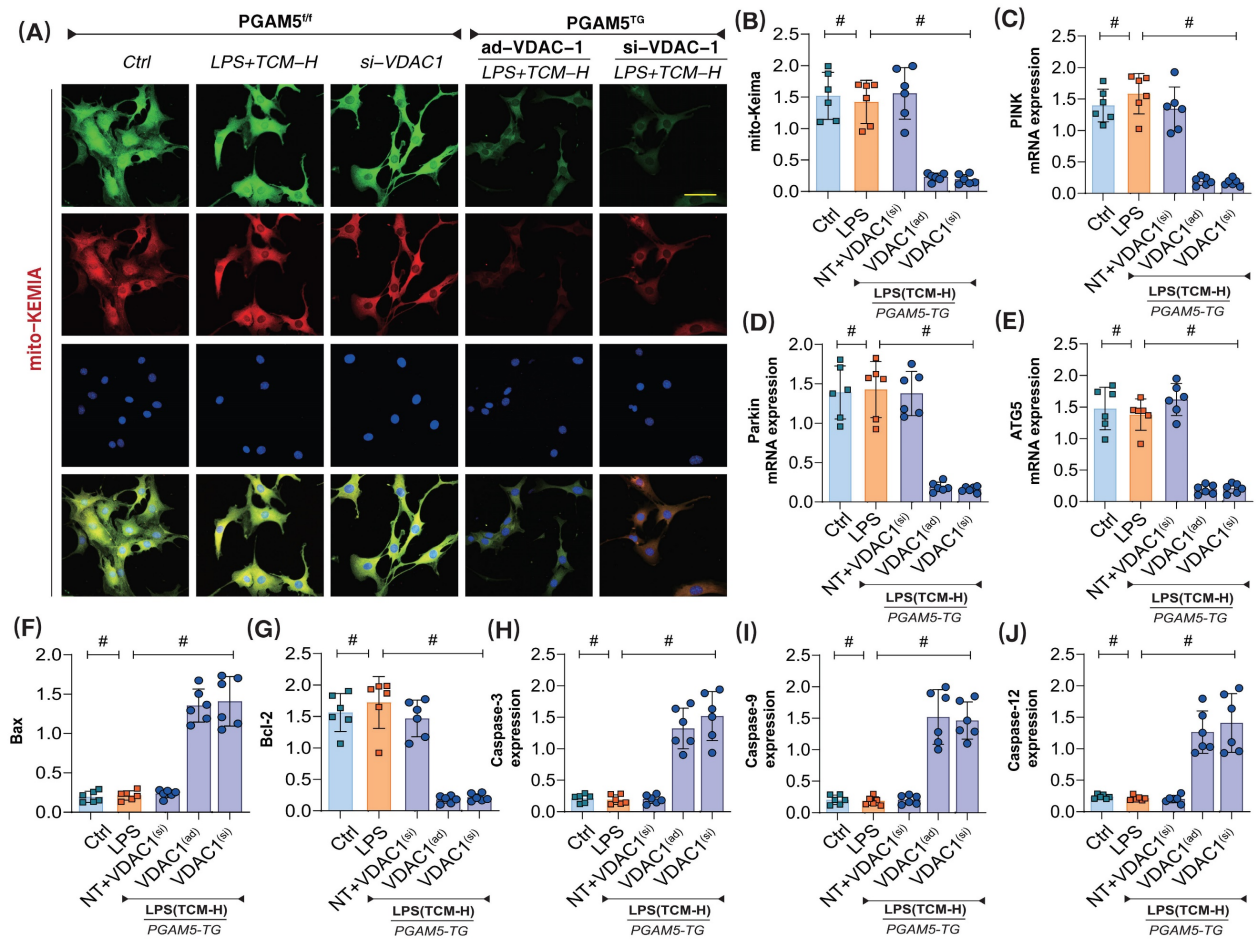
** The interaction mechanism of PGAM5-VDAC1 is involved in sepsis microvascular injury and drug action mechanism. (A-B)** Laser confocal detection of mitophagy markers;**(C)** The transcription level of PINK; **(D)** The transcription level of Parkin;** (E)** The transcription level of ATG5;** (F)** Bax activity; **(G)** BcL-2 activity; **(H)** Caspase-3 activity; **(I)**; Caspase-9 activity;** (J)** Caspase-12 activity; Experiments were repeated at least three times and the data are shown as mean ± SEM (N = 12 mice per group). *p < 0.05.

Further experiments assessed mitophagy and the expression levels of respiratory chain complexes in the context of apoptosis. The results showed that PGAM5 transgenic treatment and ad-VDAC1 eliminated the regulatory effects of TCM on mitophagy and apoptosis (Figure 7A-J). Additionally, PGAM5 transgenic treatment and si-VDAC1 treatment similarly abolished TCM's regulatory effects on mitochondrial apoptosis and mitophagy (Figure 7A-J). These results confirm that PGAM5 is likely an upstream regulatory protein of VDAC1, and TCM may further regulate VDAC1 activation through PGAM5, thereby maintaining mitochondrial homeostasis, supporting mitochondrial energy metabolism, and inhibiting apoptosis via mitochondrial pathways.

**Figure 8:**
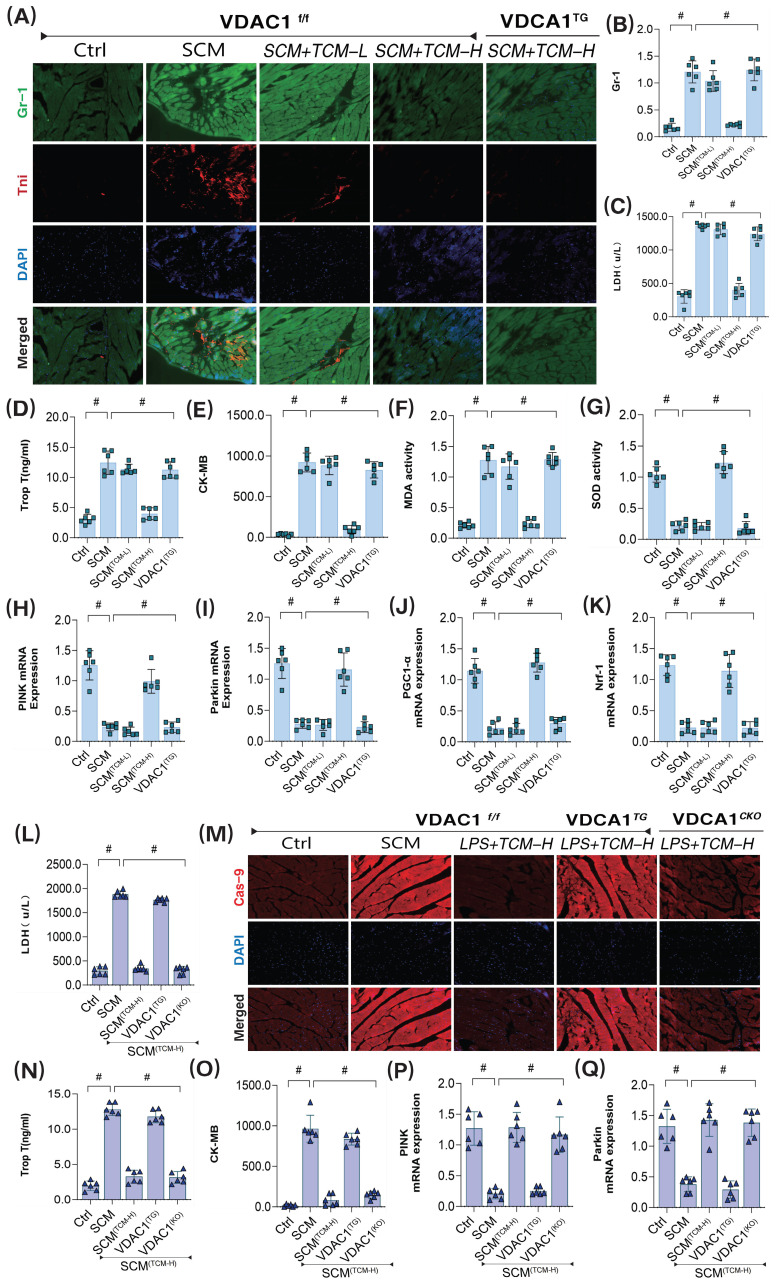
**The interaction mechanism of VDAC1 is involved in sepsis microvascular injury and drug action mechanism. (A-B)** Immunofluorescence detection of markers of sepsis mediated coronary microvascular inflammation and injury; **(C)** The expression level of LDH; **(D)** The expression level of myocardial injury marker (Tnt);** (E)** The expression level of CK-MB; **(F)** The expression level of MDA; **(G)** The expression level of SOD; **(H)** The transcription level of PINK;** (I)** The transcription level of Parkin;** (J)** The transcription level of PGC1-α;** (K)** The transcription level of Nrf-1;** (L)** The expression level of LDH;**(M)** Immunofluorescence detection of markers of sepsis mediated coronary microvascular injury; **(N)** The expression level of myocardial injury marker (Tnt); **(O)** The expression level of CK-MB; **(P)** The transcription level of PINK; **(Q)** The transcription level of Parkin; Experiments were repeated at least three times and the data are shown as mean ± SEM (N = 12 mice per group). *p < 0.05.

To further substantiate the downstream regulatory effects of PGAM5 on the VDAC1 pathway, we constructed VDAC1 transgenic animals. We employed GR-1 protein immunofluorescence, and transcriptomic analysis of mitophagy and biogenesis to verify TCM's regulatory effects on coronary microvascular damage following LPS treatment. The results confirmed that low-dose TCM did not improve coronary microvascular injury or high expression of inflammatory markers post-SCM, while high-dose TCM exhibited significant therapeutic effects on microvascular injury (Figure 8A-Q). Additionally, gene expression analysis related to mitophagy revealed that high-dose TCM significantly increased the mRNA levels of PINK, Parkin, and ATG5, activating mitophagy and biogenesis, suppressing oxidative stress damage, and further ameliorating coronary microvascular inflammation and structural damage. Notably, VDAC1 and PGAM5 transgenic treatments abolished TCM's therapeutic effects, while cardiac-specific knockouts of VDAC1 and PGAM5 did not affect TCM's efficacy (Figure 8A-Q). These results further suggest that TCM may improve LPS-mediated SCM coronary microvascular damage by regulating mitophagy through the PGAM5-VDAC1 axis.

## Discussion

In this study, we provide evidence for the targeted regulatory role of PGAM5-VDAC1 in the progression of microvascular injury caused by SCM. Through animal and cell experiments, combined with relevant genetic modification systems and metabolomics studies, we further demonstrate that PGAM5 can bind to VDAC1, inhibiting PINK/Parkin-mediated mitophagy, thereby preventing pathological mitochondrial fission, normalizing mitochondrial function, and improving microvascular injury. The primary results of this research can be summarized as: 1) LPS-induced septic myocardial injury activates PGAM5-mediated microvascular endothelial damage; 2) Knockout or silencing of PGAM5 can alleviate endothelial inflammation damage, inhibit endothelial cell apoptosis, improve myocardial structural disorder, and suppress the expression levels of cardiac injury markers; 3) At the molecular level, PGAM5 overexpression is observed after LPS-mediated inflammatory injury, inhibition of mitochondrial oxidative stress, and dysfunction of the mitochondrial respiratory chain along with abnormal opening of mPTP; 4) PGAM5-mediated mitophagy dysfunction is accompanied by abnormal balance of mitochondrial fission/fusion, impaired mitochondrial biogenesis, and abnormal activation of the unfolded protein response; 5) Mechanistically, PGAM5 can interact with VDAC1, disrupting mitochondrial homeostasis; 6) Intervention with the Puerarin can reverse PGAM5 overexpression and VDAC1 transcription levels, normalizing mitochondrial morphology and restoring mitochondrial energy metabolism. Based on our findings, this could be an important drug for regulating endothelial cell damage via mitochondrial pathways.

Research indicates that septic myocardial injury is associated with disrupted mitochondrial oxidative phosphorylation^36^, ^47^, ^48^. Under sufficient cellular oxygen conditions, the ability to produce adenosine triphosphate (ATP) decreases, leading to what is termed "cellular hypoxia," a primary cause of limited myocardial function^49^-^53^. However, studies on the activity indicators of various mitochondrial respiratory chain complexes during sepsis appear contradictory. Further experimental results reveal that the expression of complex I is reduced (indicating decreased oxidative phosphorylation coupling), complex III expression is lowered (suggesting inhibition of the electron transport chain), and complex IV expression is enhanced (indicating abnormal increased permeability of the mitochondrial inner membrane), suggesting that mitochondrial damage is related to decreased activity of respiratory chain complex enzymes. In a rat model of sepsis induced by intraperitoneal injection of lipopolysaccharide, it was found that, immediately post-injection and up to 24 hours later, mitochondrial complex III respiration declined. After six and 24 hours of injection, complex I activity decreased by 30%, with complexes II and IV demonstrating decreased activity only at the 24-hour interval^26^.

Sepsis resulted in a 33% reduction in myocardial oxygen consumption in rats, with mitochondrial oxygen consumption decreasing by 27% and complex I activity declining by 29%. These changes led to decreased mitochondrial ATP synthesis and overall levels. During sepsis, reactive oxygen species cause damage to functional proteins and biocatalysts on mitochondrial membranes, including those in myocardial mitochondrial complexes, significantly hindering mitochondrial oxygen uptake^26^, ^54^, ^55^. Increased electron leakage from mitochondria leads to elevated superoxide generation, exacerbating mitochondrial dysfunction. Further studies also found that in septic rat cardiomyocytes, due to the inhibition of complex I activity, there is an increase in superoxide anions and hydrogen peroxide, activating apoptosis via mitochondrial pathways^56^, ^57^. These findings confirm that mitochondrial energy metabolism dysfunction and respiratory chain dysfunction are important regulatory mechanisms leading to myocardial injury and intracellular homeostasis dysfunction following I/R; however, the upstream regulatory proteins and targeted mechanisms have yet to be validated^58^, ^59^.

PGAM5 is recognized as an upstream regulatory factor that modulates mitochondrial homeostasis and damage to the inner and outer mitochondrial membranes. Previous studies have revealed the anti-inflammatory effects and negative regulatory roles of PGAM5 in various diseases. Further functional regulation of related proteins has confirmed that, during myocardial ischemic injury, activation of Ripk3 can lead to upregulation of PGAM5 expression. Overexpression of PGAM5 can further activate CypD phosphorylation, which triggers necrotic apoptosis of endothelial cells via enhanced opening of the mitochondrial permeability transition pore (mPTP). However, pharmacological studies indicate that intervention with melatonin inhibits mPTP opening and downregulates PGAM5 expression, thereby blocking the Ripk3-PGAM5-CypD signaling pathway and suppressing necrotic apoptosis in endothelial cells^60^.

This is consistent with our findings, which demonstrate that LPS-mediated septic damage further results in multi-pathway injury to myocardial or coronary microvasculature. Both myocardial and microvascular tissues display different levels of apoptosis and necrotic cell death, alongside irregular opening of the mitochondrial permeability transition pore (mPTP) and disturbances in mitochondrial energy metabolism^60^-^62^. These factors may represent critical pathways for microvascular endothelial injury. Further research also shows that inhibiting PGAM5 can lead to reduced expression of Drp1, thereby preventing significant necrotic apoptosis in myocardial cells following ischemia-reperfusion treatment. There exists a positive feedback and regulatory relationship between RIPK1 and PGAM5, suggesting that PGAM5 may serve as a novel therapeutic target for the prevention of myocardial ischemia-reperfusion (I/R) injury^63^. Our results also suggest that microvascular endothelial cells exhibit significant redox imbalance following septic damage, accompanied by abnormal activation of RIPK-VDAC1. This process mediates the activation of Drp1 and Fis1, leading to mitochondrial structural damage and suppression of mitophagy levels. In other stress-inducing environments, myocardial stress injury is associated with increased expression of mitochondrial fission proteins and SP1, along with decreased levels of p-AMPK and Drp1^S637^. Notably, EMPA can reverse these phenotypic changes, further inhibiting PGAM5 overexpression and pathological mitochondrial fission, while also modulating the interaction between the SP1 and PGAM5 promoters, thus improving myocardial cell viability^64^.

In addition to mitochondrial fission, numerous studies have demonstrated that indirectly activating mitochondrial biogenesis can improve survival rates in septic animals, whereas inhibiting mitochondrial biogenesis can worsen prognosis^26^. However, some studies indicate that cardiomyocyte-specific overexpression of PGC-1α significantly increases mitochondrial biogenesis, yet this increase does not excessively trigger endotoxemia^26^, ^65^. Moreover, enhanced mitochondrial biogenesis may alter mitochondrial dynamics, potentially damaging mitochondrial function. Thus, we hypothesize that upstream regulation of mitophagy is crucial for improving endothelial/myocardial damage induced by sepsis^66^. Our previous research also showed that following LPS-induced stress, wild-type mice exhibited significant upregulation of myocardial VDAC1 transcription and expression, accompanied by characteristic changes in myocardial contractile/relaxation function, cardiac inflammation, and cell death. Notably, these changes were exacerbated in TMBIM6^CKO^ mice treated with LPS, while TMBIM6^TG^ mice showed minimal effects. In primary cardiomyocytes treated with LPS, the lack of TMBIM6 led to additional impairment of mitochondrial respiration and ATP production, with mitochondrial dysregulation occurring due to increased fission, reduced mitophagy, and disturbances in mitochondrial biogenesis. TMBIM6 can exert cardioprotective effects against LPS-induced sepsis by interacting with mitochondrial Ca^2+^ uptake, inhibiting/blocking overexpression of the VDAC1, and preventing its oligomerization^26^. This is consistent with our findings that abnormal activation of mitochondrial VDAC1 may mediate important pathways of programmed and non-programmed cell death in endothelial cells, suggesting that upstream target modulation of PGAM5 could represent a potential therapeutic target. Based on prior research, we further confirmed that mitophagy dysfunction and regulation of the PGAM5-VDAC1 axis may contribute to metabolic abnormalities in late sepsis.

Currently, this study has several limitations. Firstly, based on our previous research, we have yet to identify the upstream targets and proteins of PGAM5, nor have we further validated their interaction. Additionally, the phenotypic regulatory mechanisms of the PGAM5-VDAC1 interaction across different animal models remain unexplored. Furthermore, we have not confirmed the receptor-dependent and non-receptor-dependent pathways regulating mitophagy dysfunction following SCM, necessitating future validation through post-translational modifications and gene knockout models. Thirdly, further experimental results have yet to elucidate whether there are upstream and downstream interactions between PGAM5-mediated mitophagy, mitochondrial fission, mitochondrial biogenesis, and the unfolded protein response. In future studies, we aim to confirm these interactions through genetically modified animal models with various phenotypes. Fourthly, we have not validated the targeted effects of PGAM5 and its regulatory role on mitophagy through other drug components or compound therapies, warranting pharmacological experiments to confirm these interactions and related mechanisms.

To conclude, the findings imply that PGAM5 serves a protective function against coronary microvascular injury caused by SCM by normalizing mitophagy processes. Mechanistically, TCM can regulate mitochondrial homeostasis and endothelial cell activity through mitophagy and mito-dynamics, thereby modulating endothelial damage. The results of our study position PGAM5 as a newly identified pathway in the pathogenesis of SCM, while TCM serves as a valuable intervention that may address endothelial and microvascular damage through mitochondrial pathways, ultimately targeting the improvement of microcirculatory dysfunction following septic cardiomyopathy.

## Supplementary Material

Supplementary figures and tables.

## Figures and Tables

**Table 1:** Primer sequences

Gene	Forward sequence	Reverse sequence	Product length/bp
PGC1-α	GAATCAAGCCACTACAGACACCG	CATCCCTCTTGAGCCTTTCGTG	136
CHOP	GGAGGTCCTGTCCTCAGATGAA	GCTCCTCTGTCAGCCAAGCTAG	122
ATF5	GCTCGTAGACTATGGGAAACTCC	CAGTCATCCAATCAGAGAAGCCG	137
ATG5	CTTGCATCAAGTTCAGCTCTTCC	AAGTGAGCCTCAACCGCATCCT	107
IL-10	CGGGAAGACAATAACTGCACCC	CGGTTAGCAGTATGTTGTCCAGC	130
Nrf1	GGCAACAGTAGCCACATTGGCT	GTCTGGATGGTCATTTCACCGC	141
IL-17	CAGACTACCTCAACCGTTCCAC	TCCAGCTTTCCCTCCGCATTGA	130
Mfn1	CCAGGTACAGATGTCACCACAG	TTGGAGAGCCGCTCATTCACCT	149
HSP60	TGCTCATCGGAAGCCATTGGTC	TTGACTGCCACAACCTGAAGACC	108
HSP10	GCCGAAACTGTAACCAAAGGTGG	CTCCAACTTTCACACTGACAGGC	139
LonP1	CCAAGCATGTGATGGACGTGGT	GTCCAAGTTCTCATCACTCTGCC	146
ClpP	ATCGCCATCCAGGCAGAGGAAA	ATGTAGCGGTCCCTCTCCATTG	119
Tfam	GAGGCAAAGGATGATTCGGCTC	CGAATCCTATCATCTTTAGCAAGC	116
Caspase-12	CAGATGAGGAACGTGTGTTGAGC	GGAACCAGTCTTGCCTACCTTC	131
Caspase-3	GGAGTCTGACTGGAAAGCCGAA	CTTCTGGCAAGCCATCTCCTCA	113
MMP-9	GCTGACTACGATAAGGACGGCA	TAGTGGTGCAGGCAGAGTAGGA	136
Caspase-9	GCTGTGTCAAGTTTGCCTACCC	CCAGAATGCCATCCAAGGTCTC	124
Drp-1	GCGAACCTTAGAATCTGTGGACC	CAGGCACAAATAAAGCAGGACGG	104
Fis1	GCTGGTTCTGTGTCCAAGAGCA	GACATAGTCCCGCTGTTCCTCT	144
Opa1	TCTCAGCCTTGCTGTGTCAGAC	TTCCGTCTCTAGGTTAAAGCGCG	103
TNF-a	GGTGCCTATGTCTCAGCCTCTT	GCCATAGAACTGATGAGAGGGAG	139
Bax	AGGATGCGTCCACCAAGAAGCT	TCCGTGTCCACGTCAGCAATCA	103
Bcl-2	CCTGTGGATGACTGAGTACCTG	AGCCAGGAGAAATCAAACAGAGG	123
Pink1	CGACAACATCCTTGTGGAGTGG	CATTGCCACCACGCTCTACACT	173
Parkin	CCAGAGGAAAGTCACCTGCGAA	GTTCGAGCAGTGAGTCGCAATC	110
