# Supplementary material for: High-Throughput Screening of an FDA-Approved Compound Library Reveals a Novel GAS6 Receptor Agonist for Therapeutic Intervention in Septic Myocardial and microvascular Injury via Modulation of Danger-Associated Molecular Patterns
Source: Int J Biol Sci. 2024 Nov 11;20(15):6222–40. doi: 10.7150/ijbs.104427 (PMC11628332; doi:10.7150/ijbs.104427)
Supplement: Supplementary file 1 — Supplementary figures and tables. [file ijbsv20p6222s1.pdf]

Supplementary Figure 1

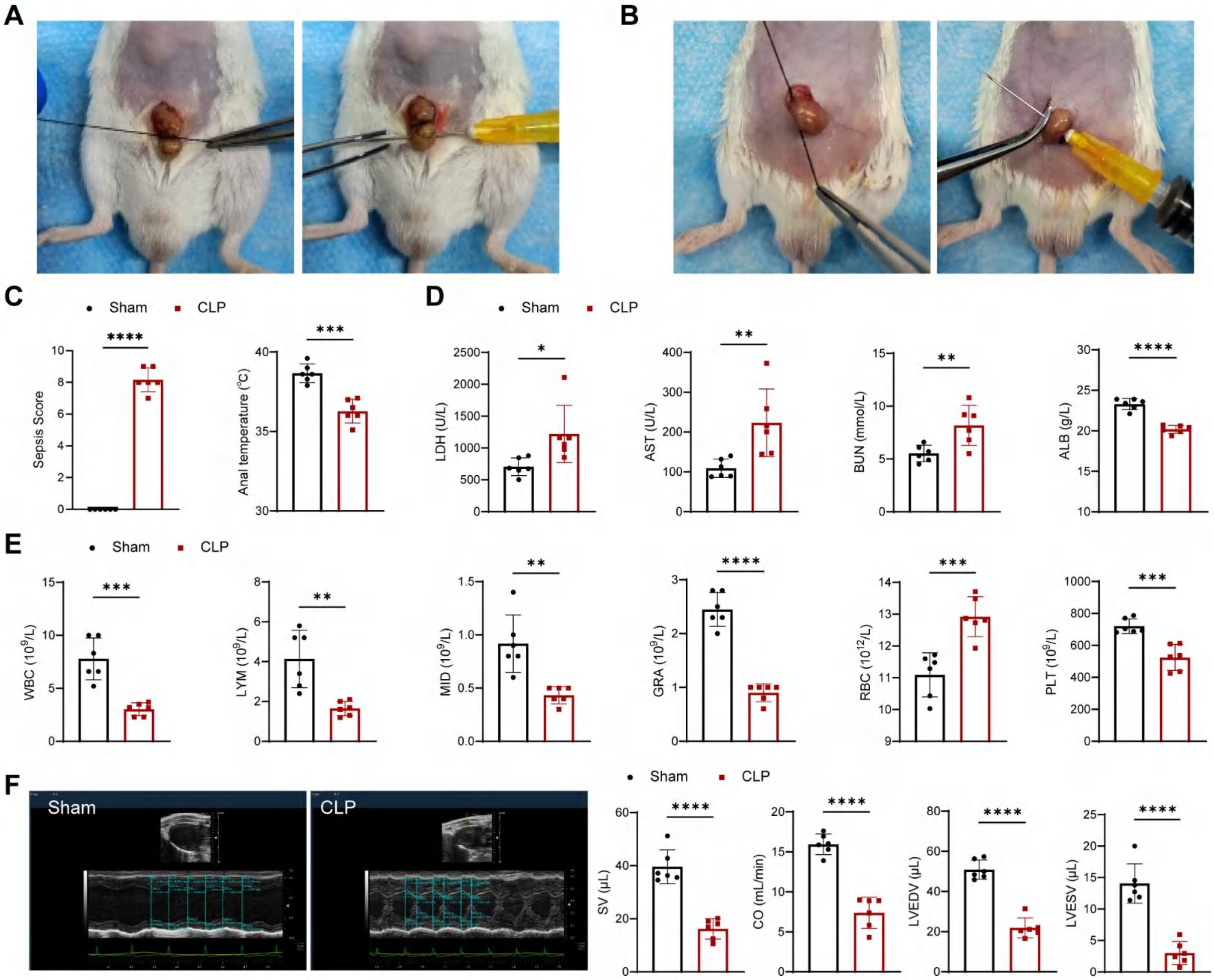

**Supplementary Figure 2**

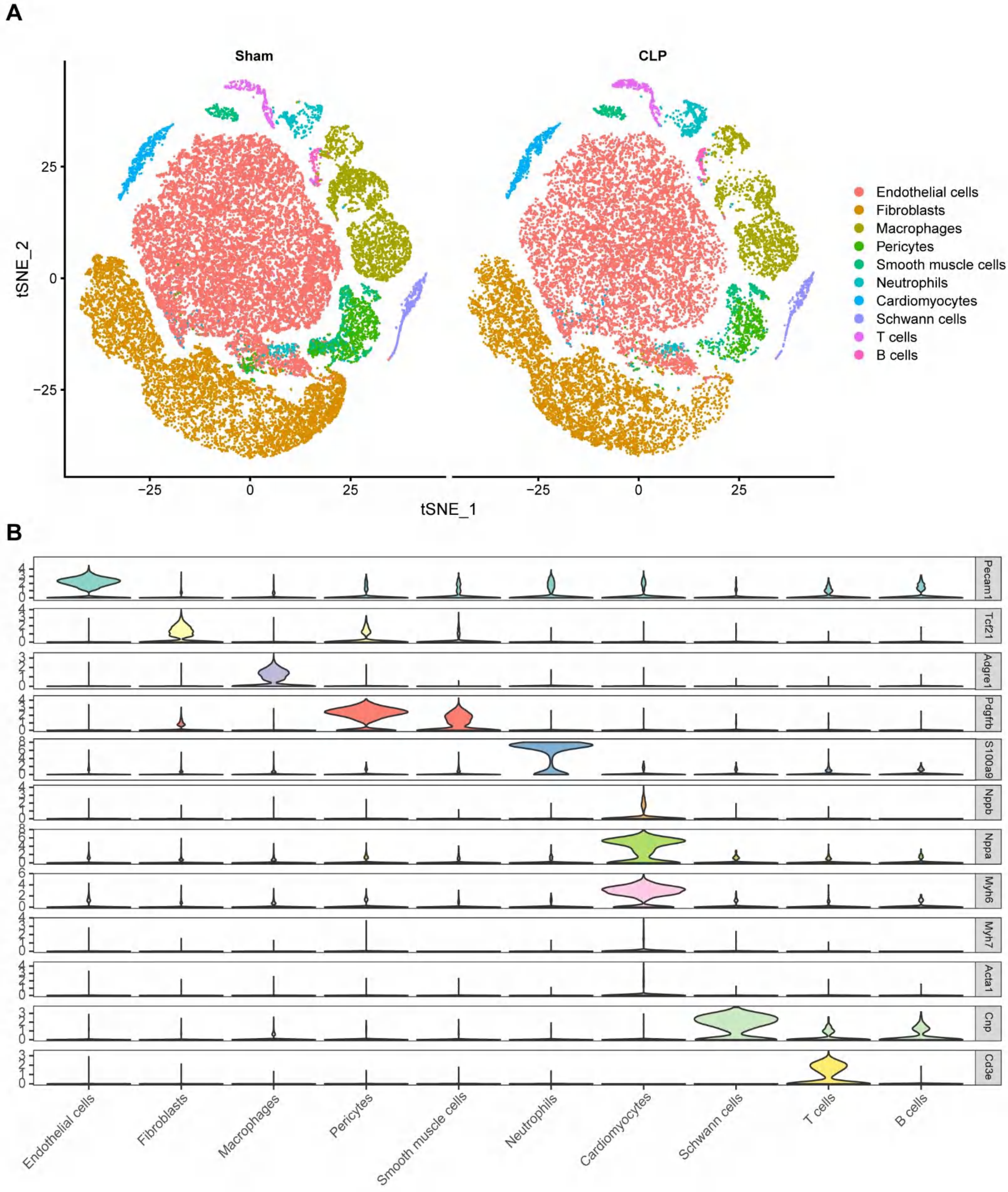

**Supplementary Figure 3**

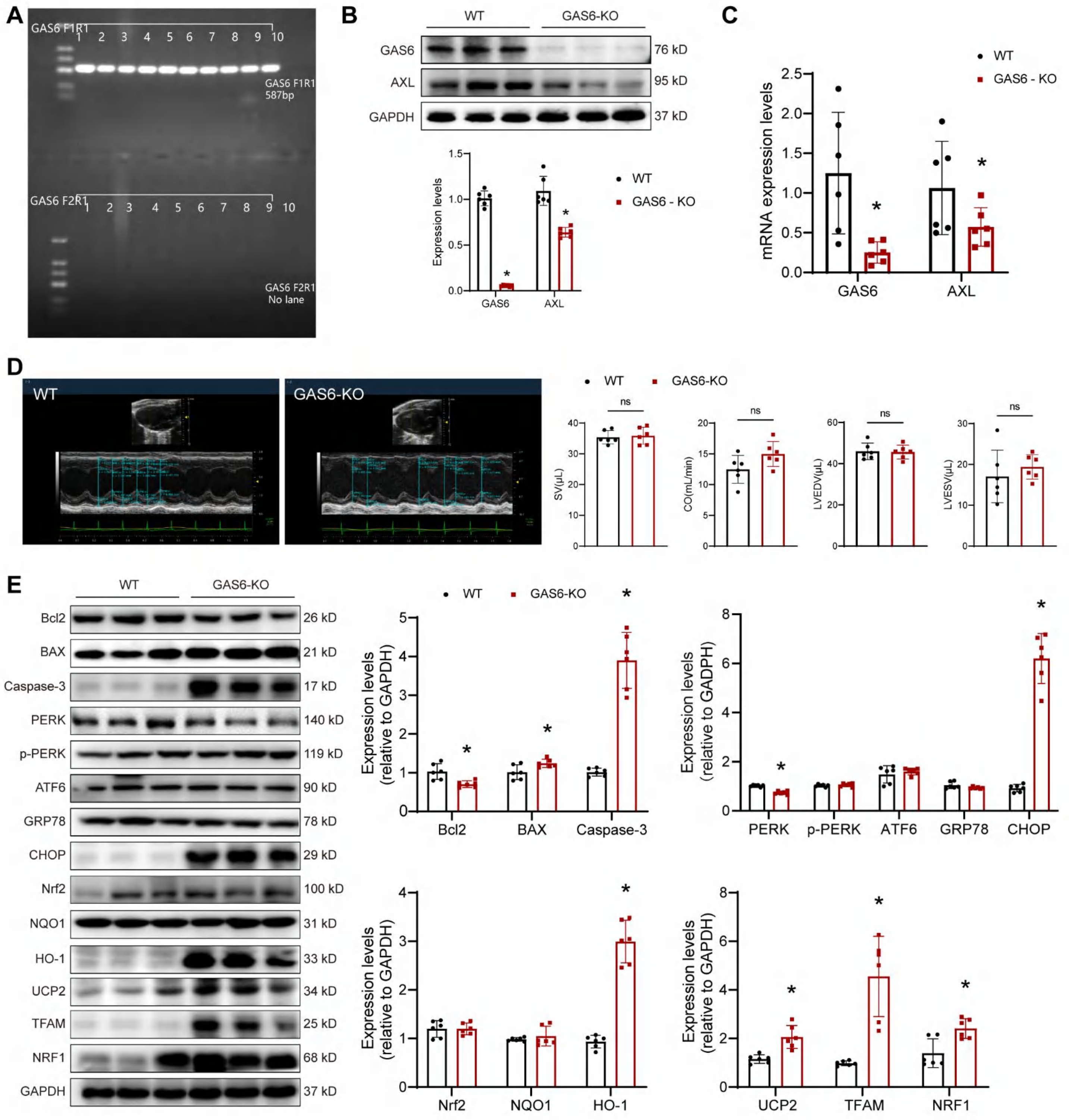

**Supplementary Figure 4**

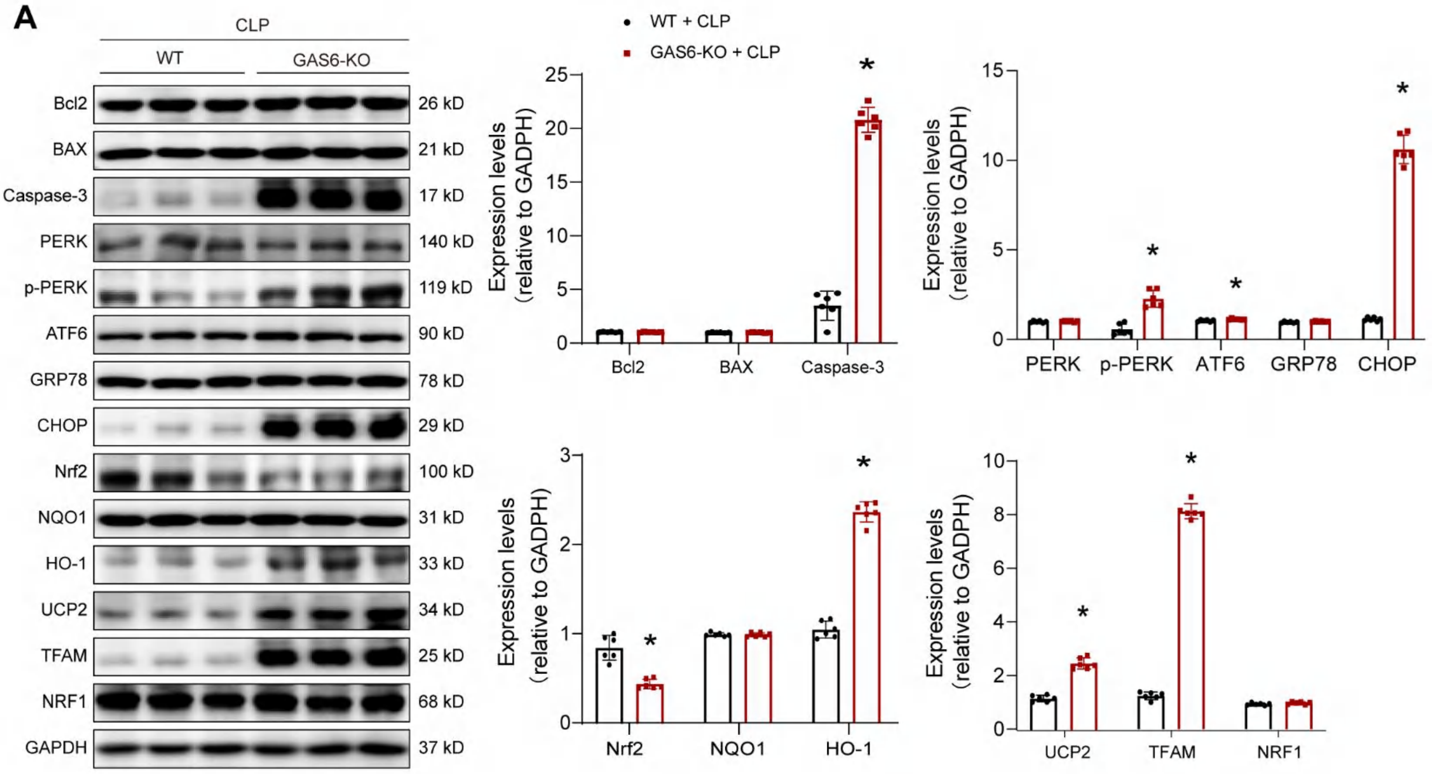

**Supplementary Figure 10**

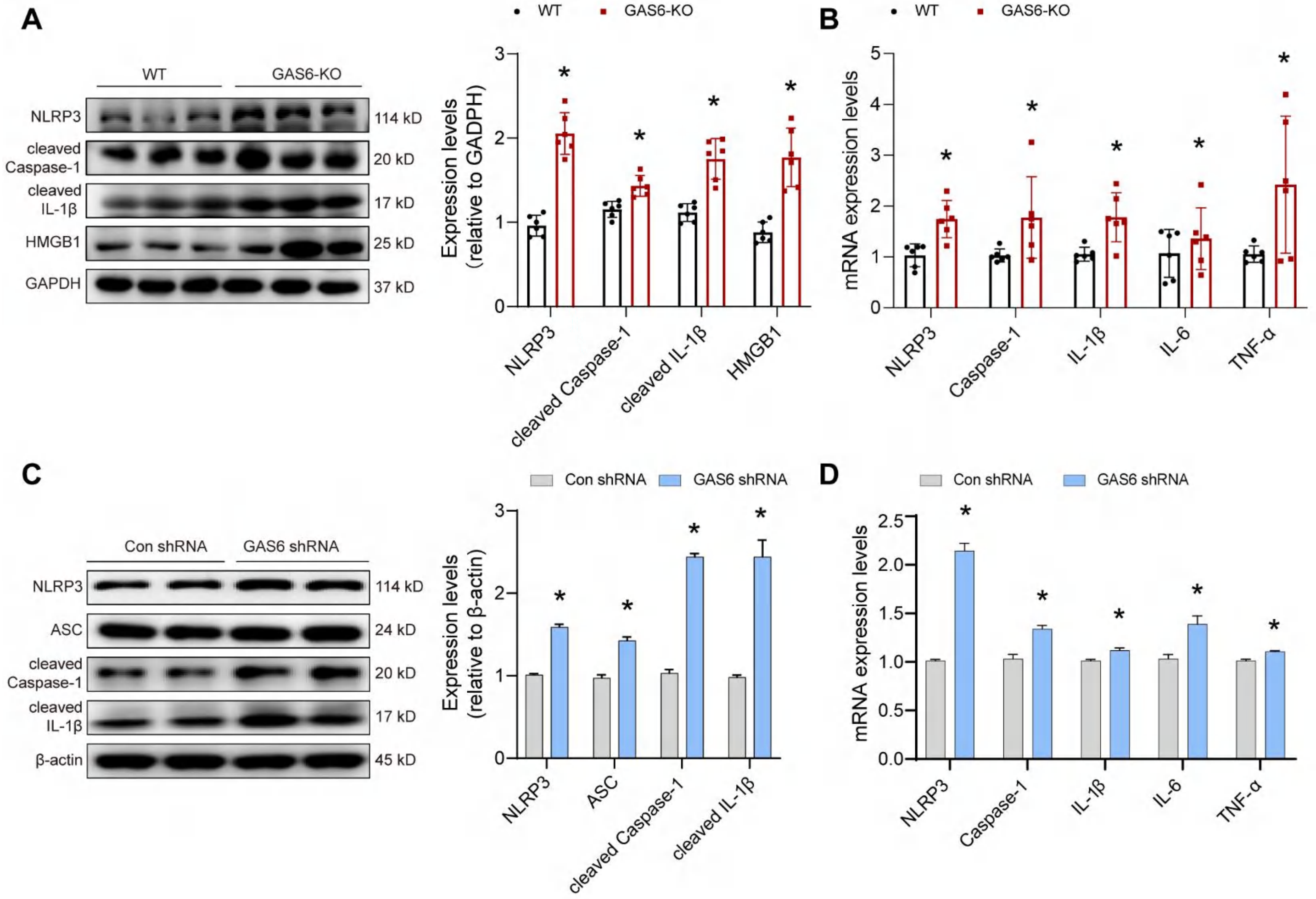

**Supplementary Figure 5**

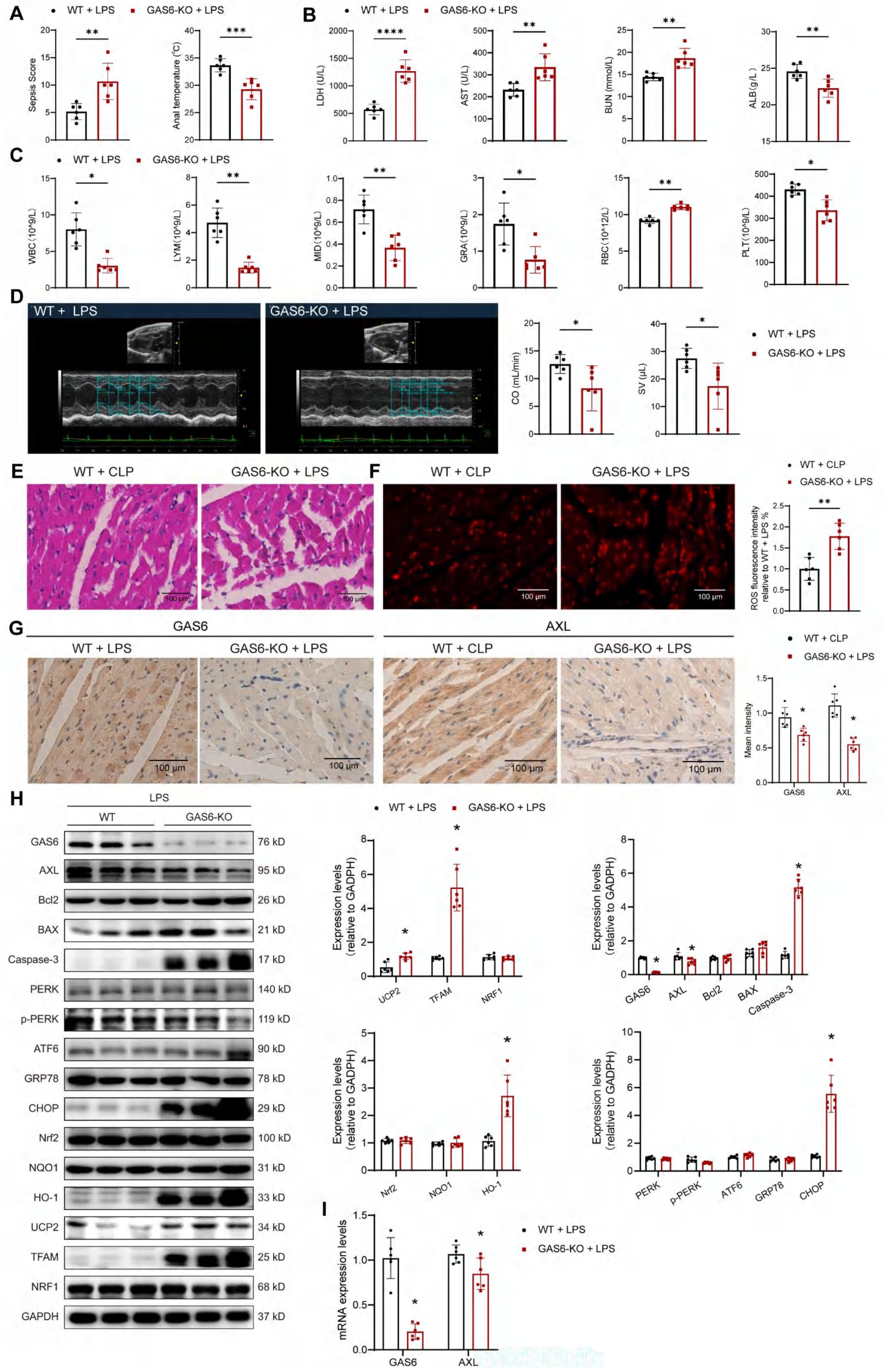

**Supplementary Figure 11**

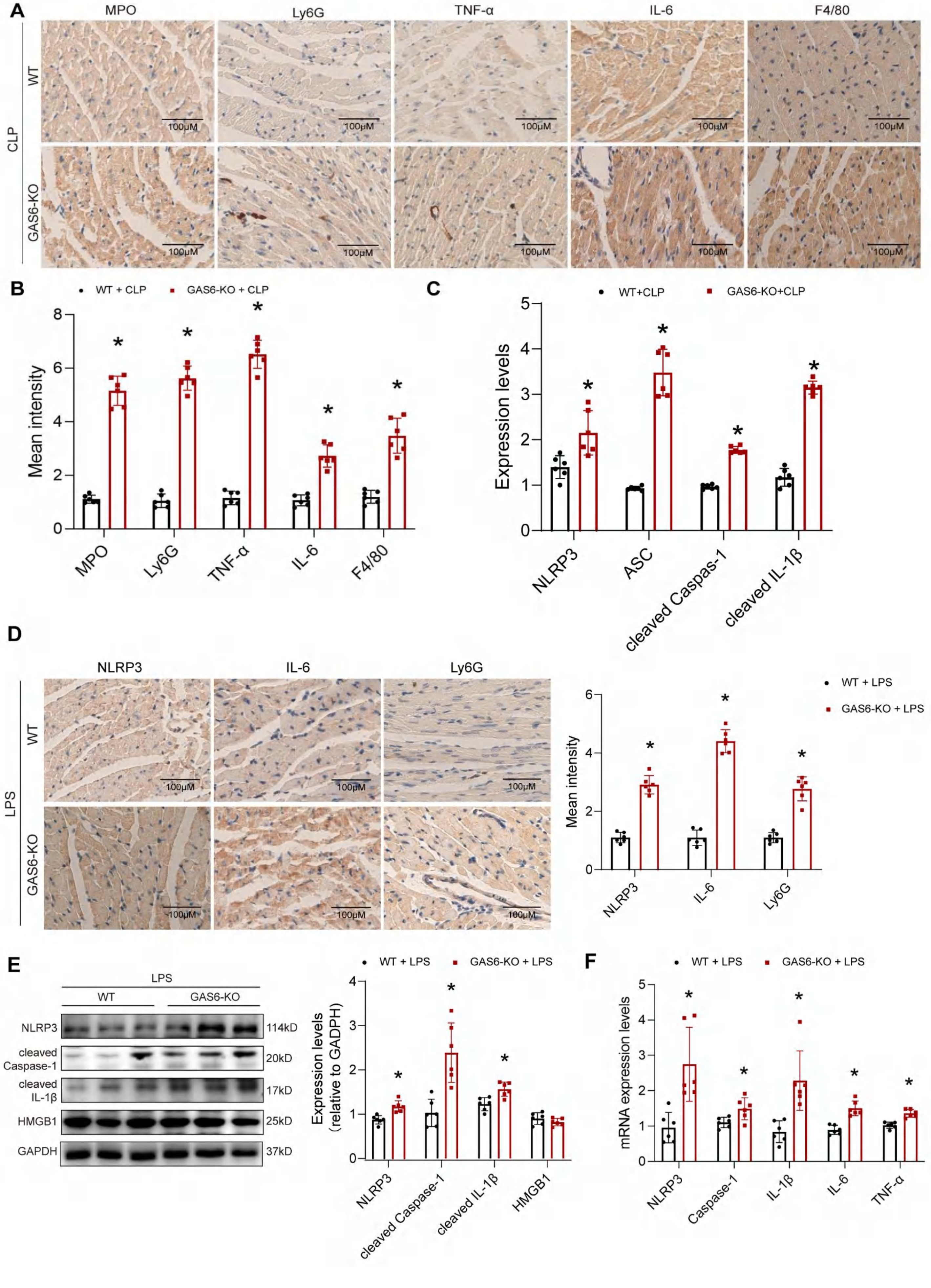

**Supplementary Figure 12**

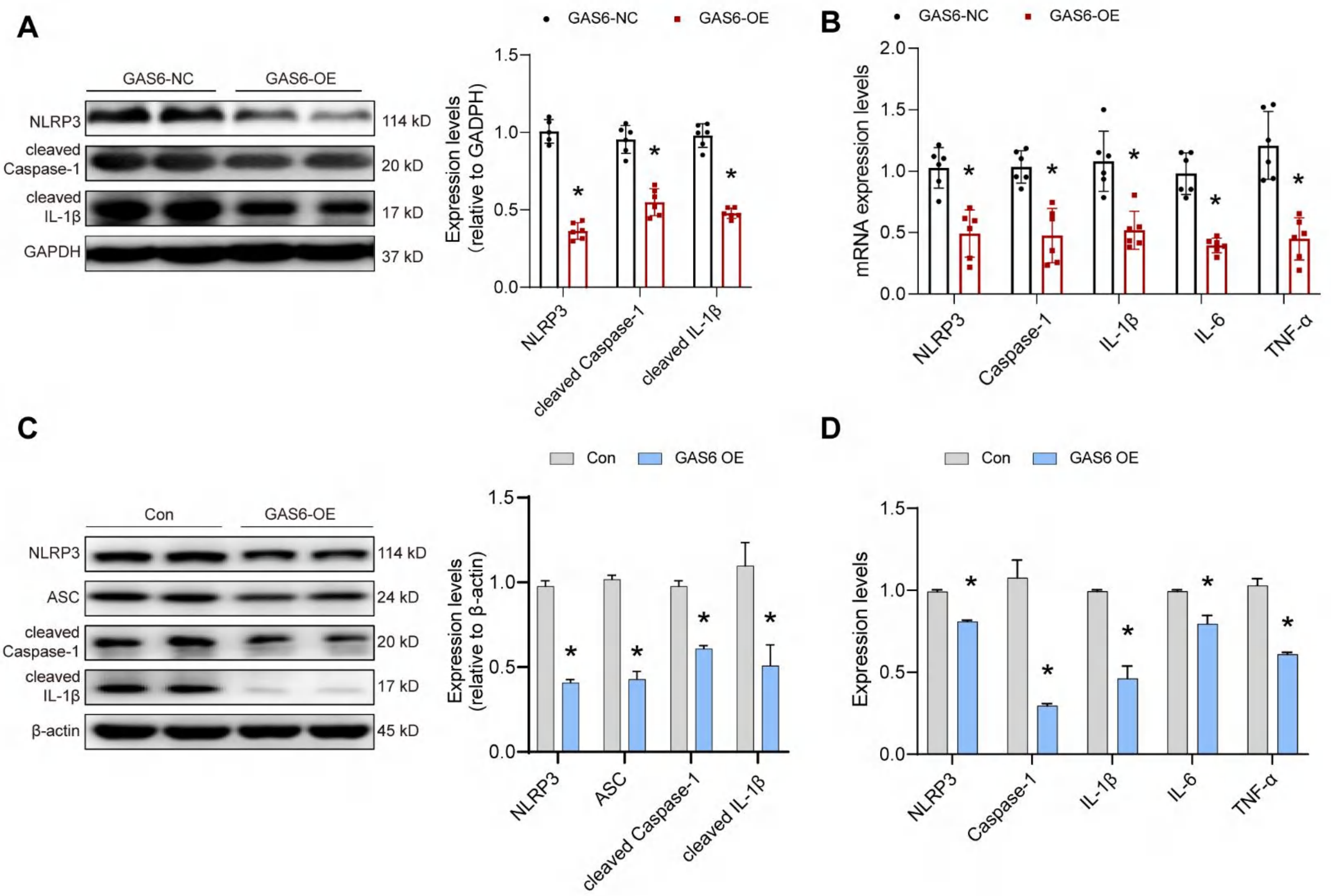

**Supplementary Figure 6**

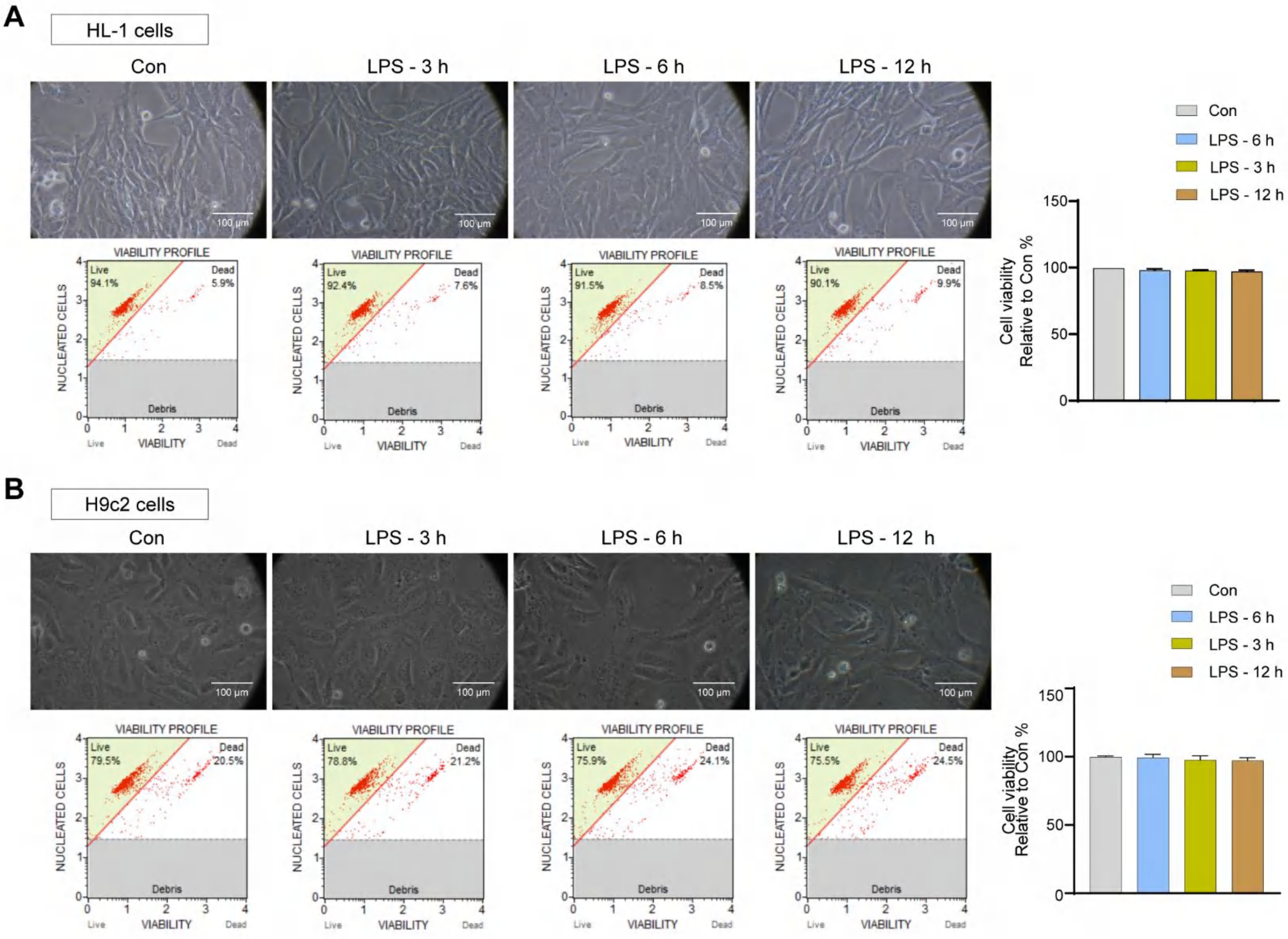

**Supplementary Figure 13**

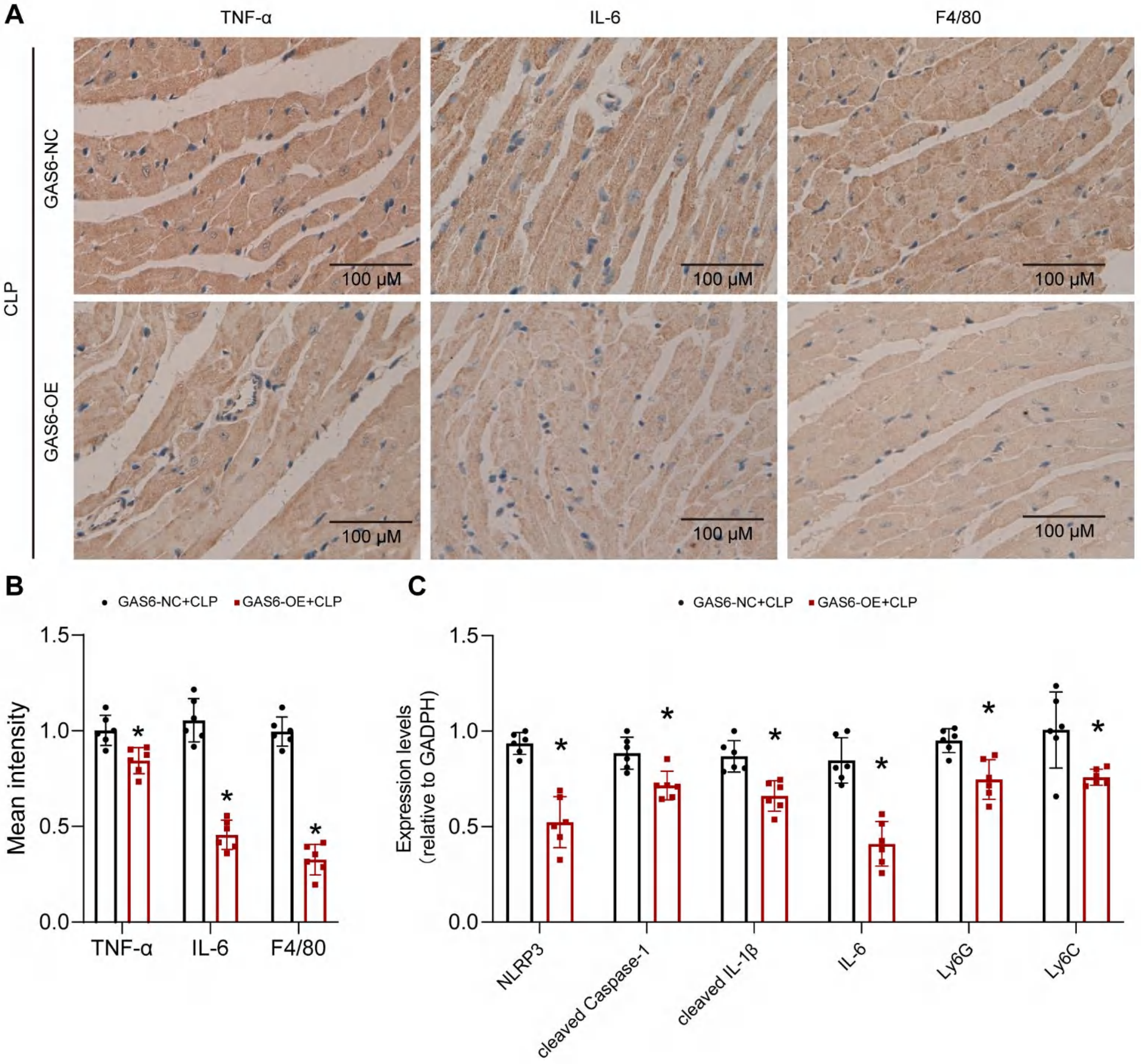

**Supplementary Figure 7**

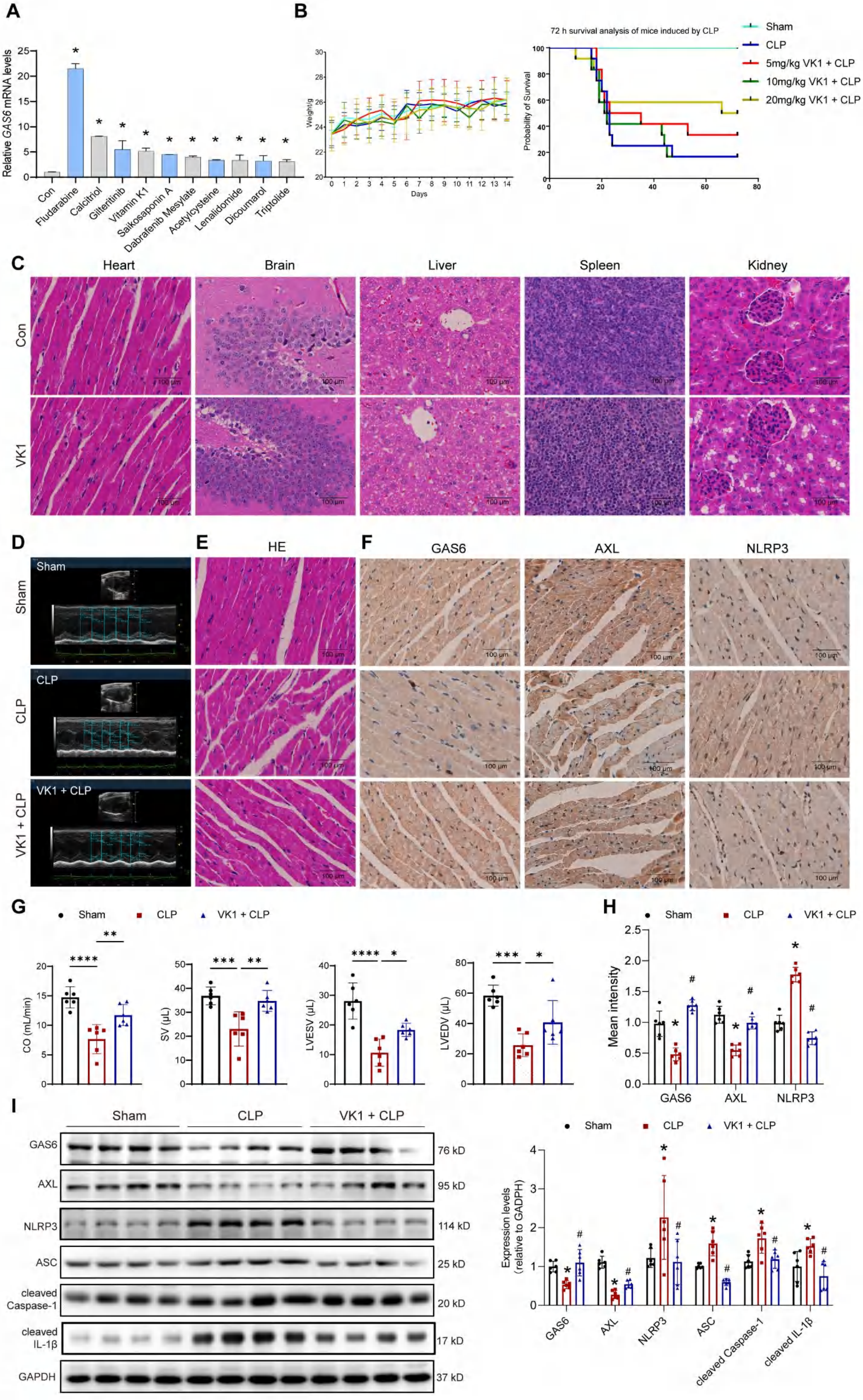

**Supplementary Figure 8**

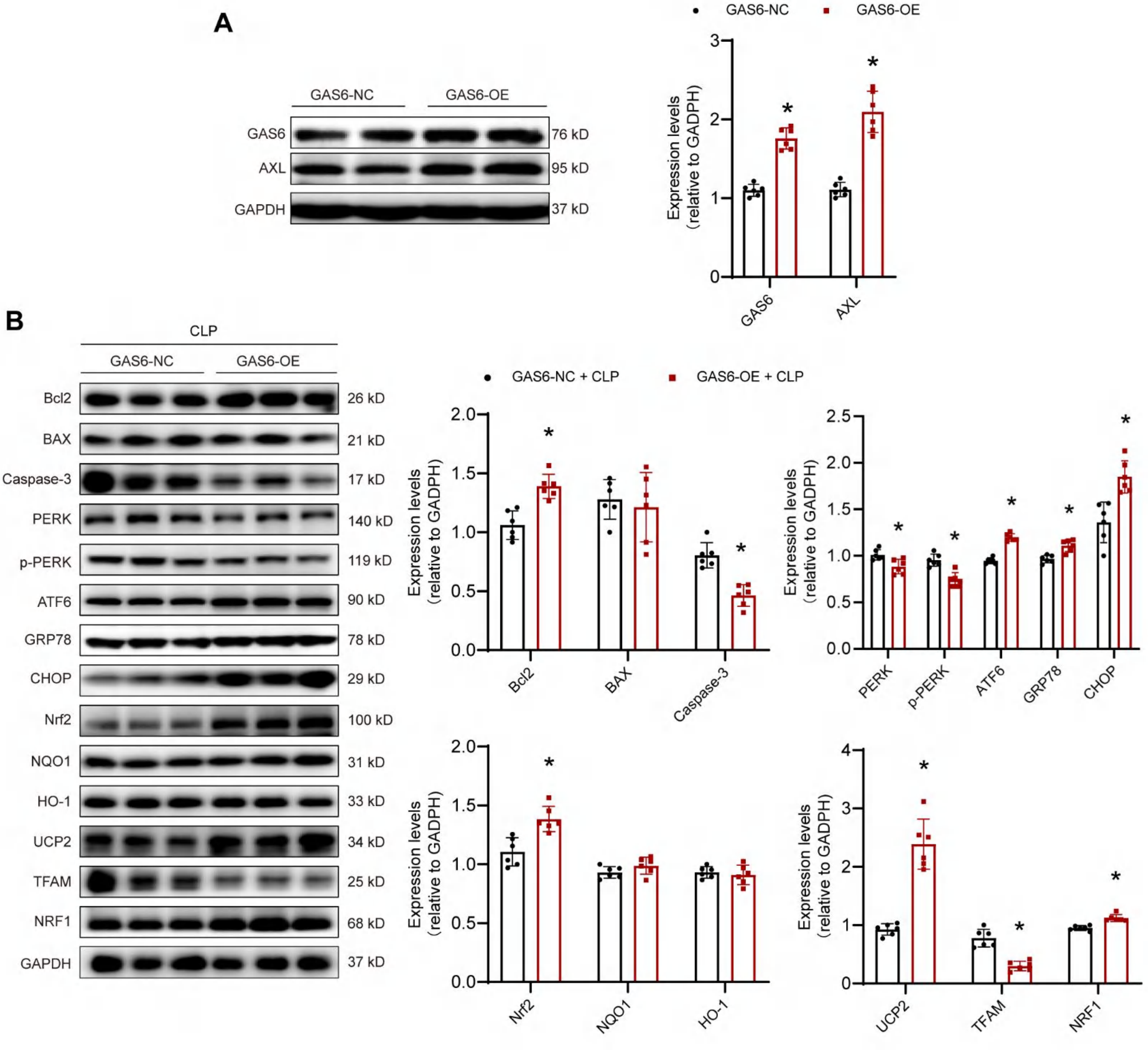

**Supplementary Figure 14**

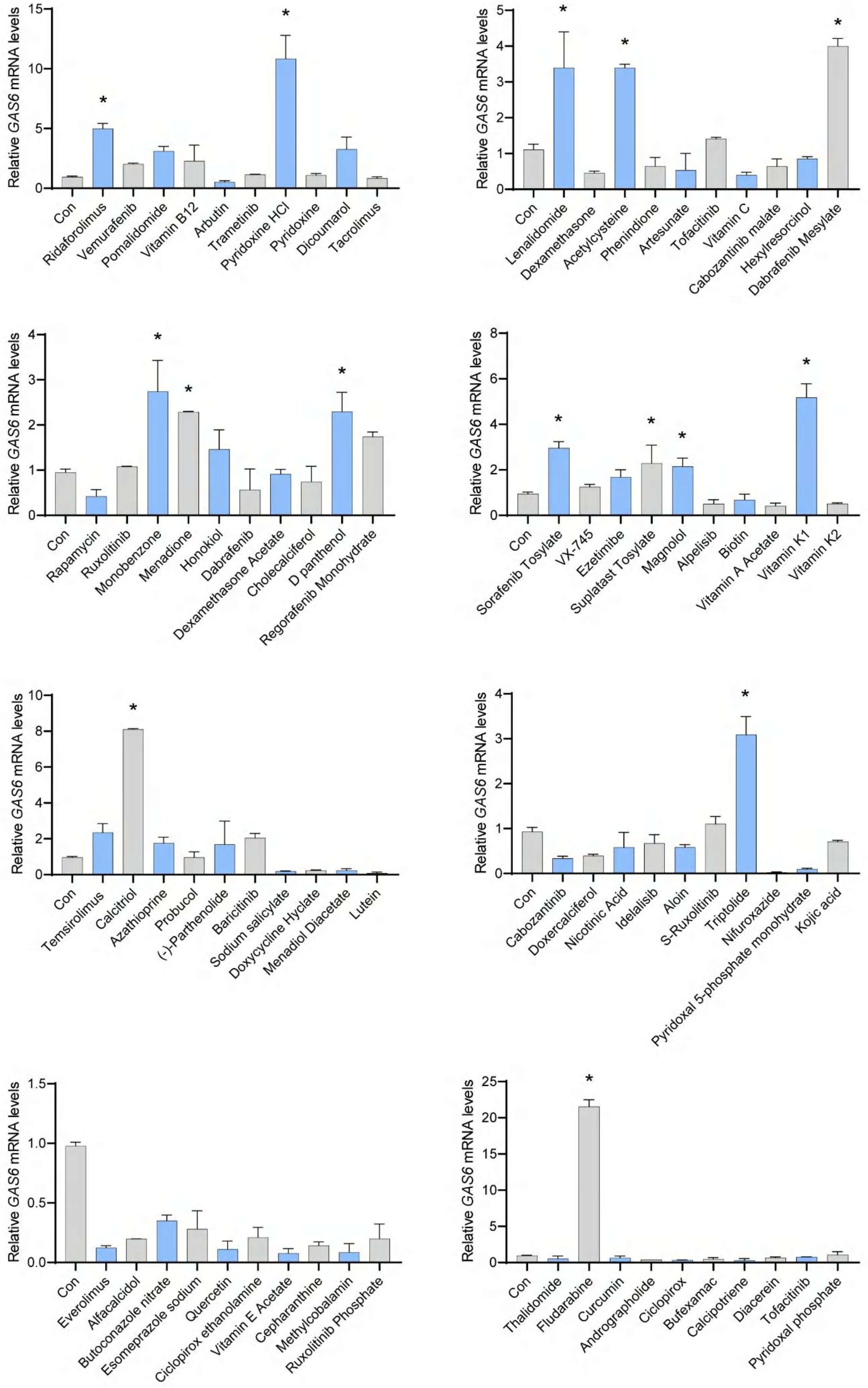

**Supplementary Figure 9**

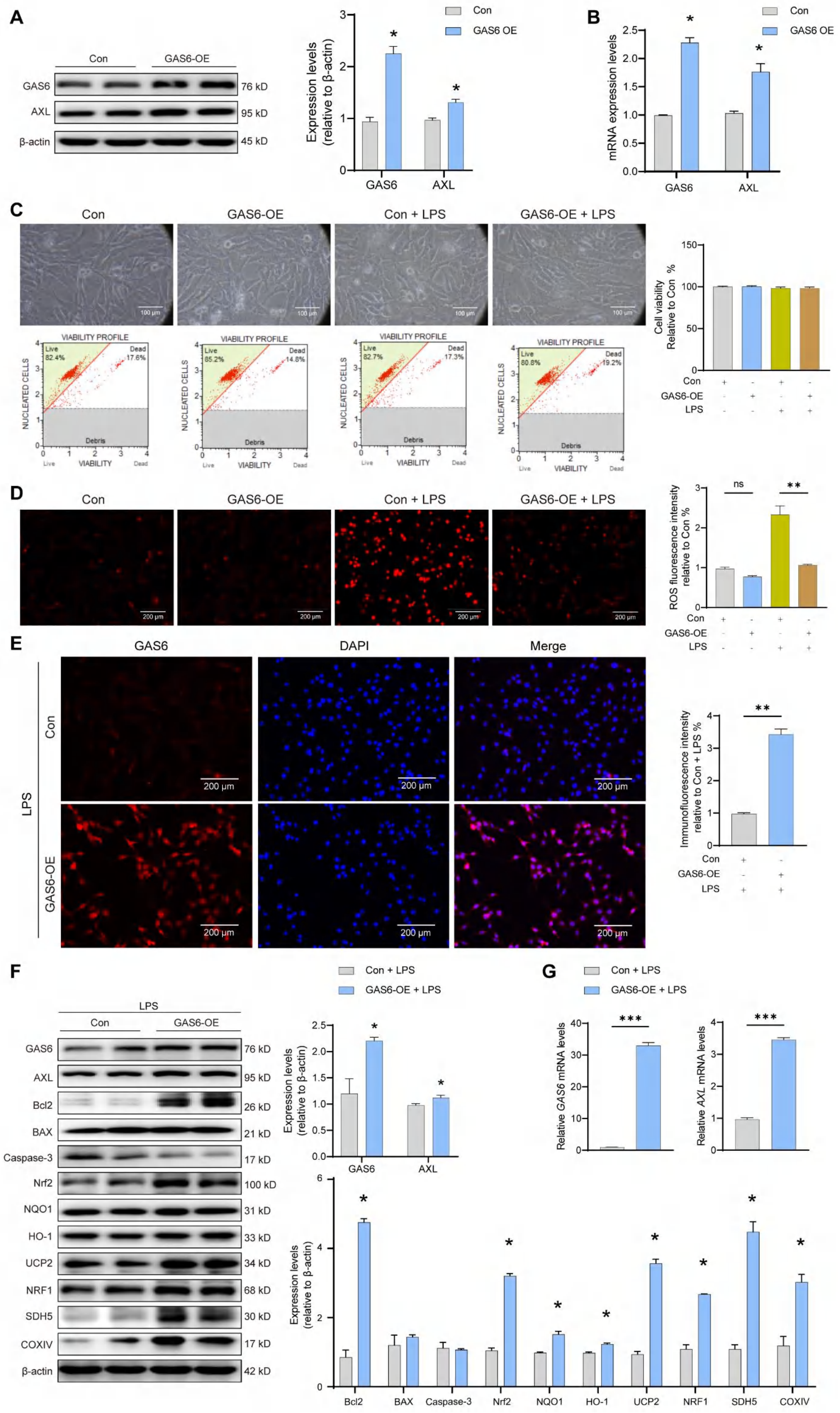

**Supplementary Figure 15**

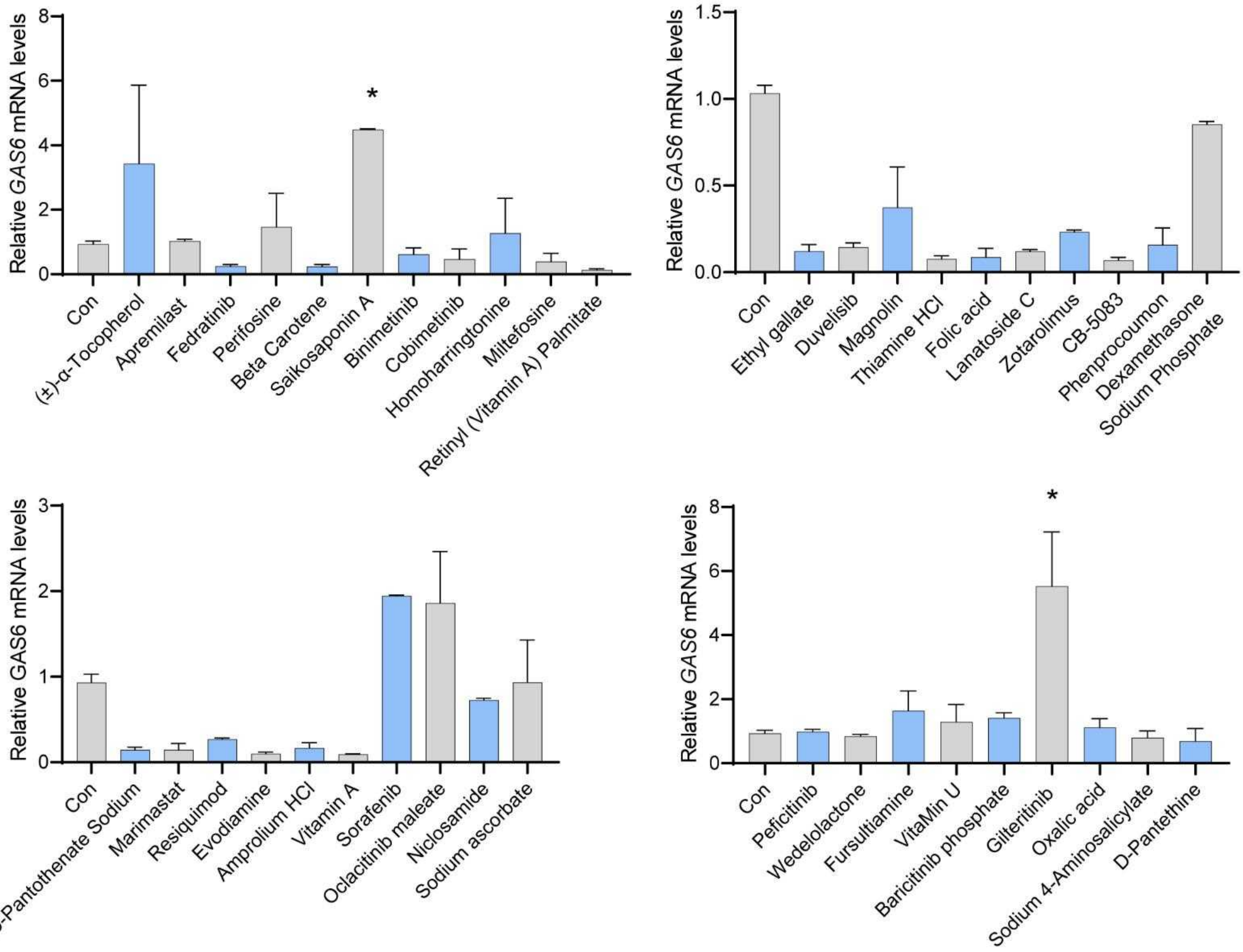

**Supplementary Figure 16**

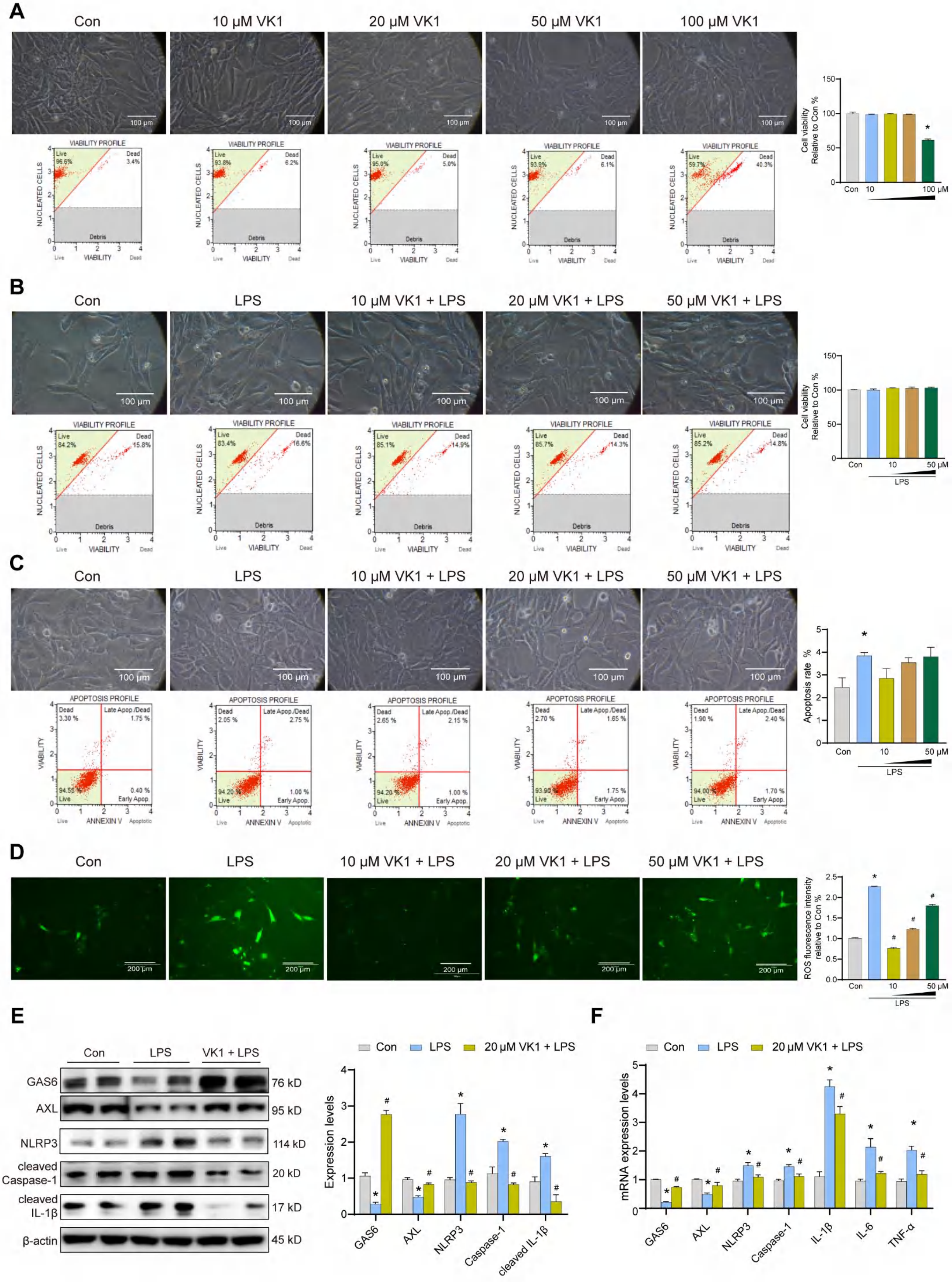

**Supplementary Figure 17**

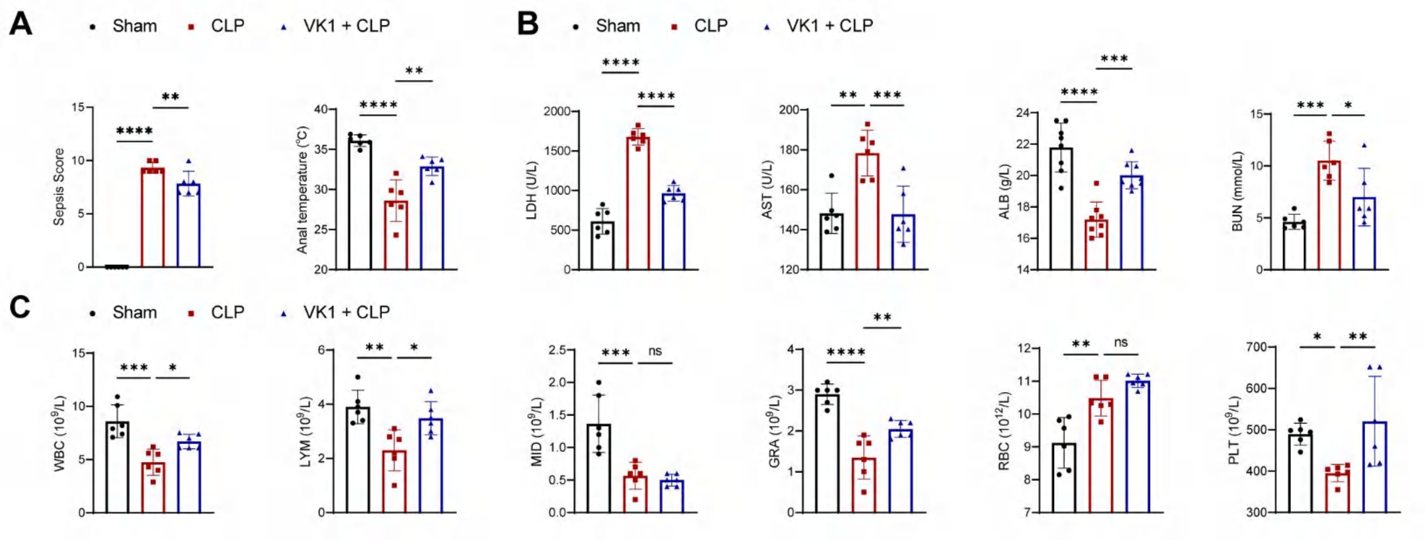

**Supplementary Figure 18**

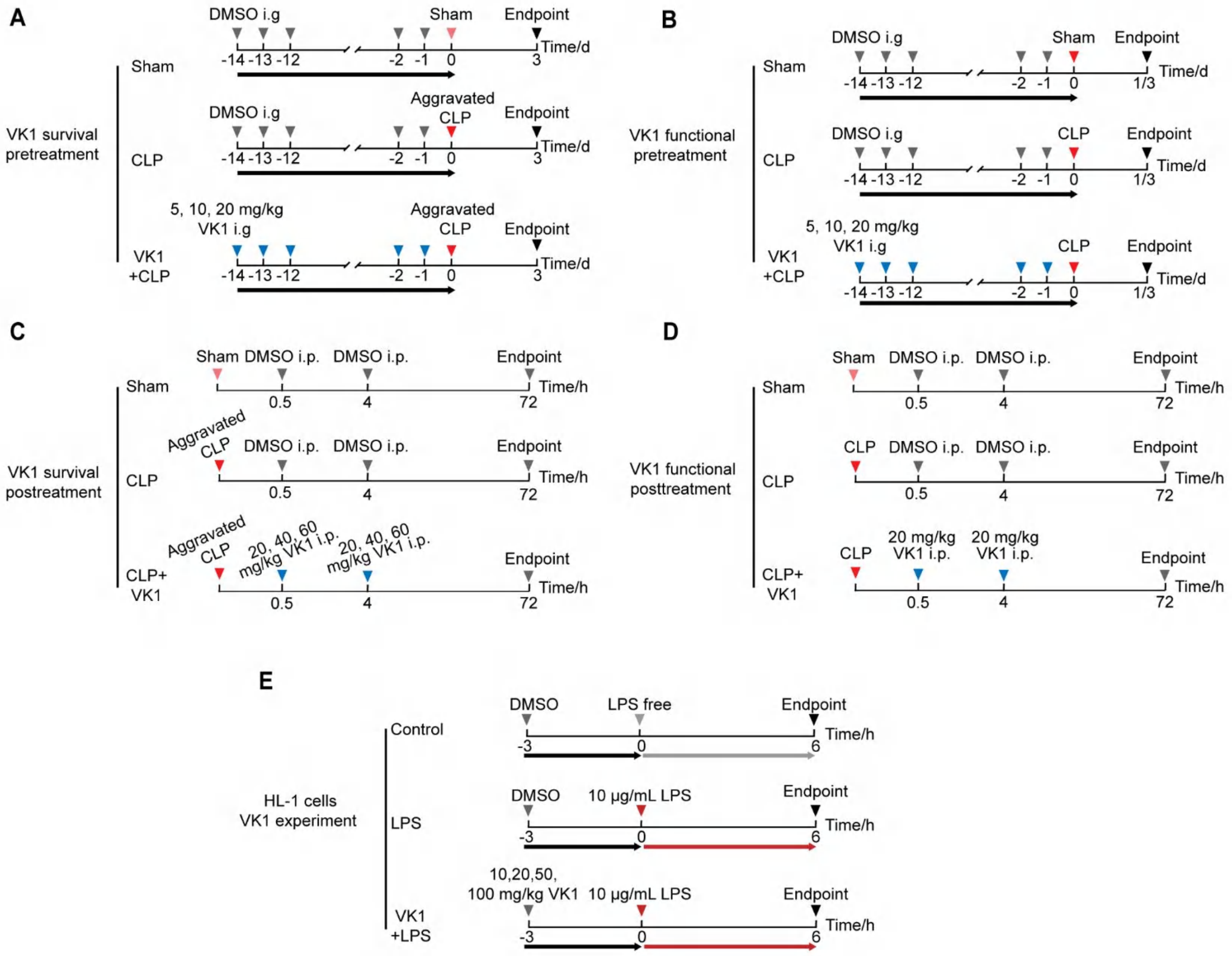

# SUPPLEMENTAL Materials

## SUPPLEMENTAL TABLES

**Table S1 Baseline characteristics of patients with sepsis and non-sepsis.**

| Variables | Total(n=40) | Non-Sepsis (n=20) | Sepsis (n=20) | P value |
|---|---|---|---|---|
| **Age (years)** | 55.18±15.52 | 57.10±14.02 | 53.25±16.66 | 0.4456 |
| **Male gender (%)** | 26(65.00%) | 14(70.00%) | 12(60.00%) | 0.5070 |
| **BMI** | 24.8125±3.76 | 23.77±3.97 | 25.86±3.43 | 0.2220 |
| **SOFA** | —— | —— | 12.35±3.26 | —— |
| **Renal dysfunction** | 7(17.50%) | 3(15.00%) | 4(20.00%) | 0.9990 |
| **Respiratory dysfunction** | 7(17.50%) | 1(5.00%) | 6(30.00%) | 0.0960 |
| **Liver dysfunction** | 13(32.500%) | 4(20.00%) | 9(45.00%) | 0.0910 |
| **Heart dysfunction** | 9(22.50%) | 2(10.00%) | 7(35.00%) | 0.1130 |
| **Diabetes mellitus** | 9(22.50%) | 4(20.00%) | 5(25.00%) | 0.9990 |
| **Fatty liver** | 5(12.50%) | 3(15.00%) | 2(10.00%) | 0.9990 |
| **ALT** | 35.92±38.15 | 26.80±20.58 | 45.05±48.17 | 0.1371 |
| **AST** | 46.70±56.48 | 25.9±14.14 | 67.50±72.90 | 0.0194 |
| **GGT** | 50.90±66.76 | 27.9±15.35 | 73.90±87.29 | 0.0295 |
| **ALP** | 116.78±107.25 | 84.4±26.82 | 149.15±142.09 | 0.0584 |
| **RBC** | 3.88±1.70 | 4.93±1.83 | 2.87±0.58 | <0.0001 |
| **HGB** | 109.14±30.89 | 133.22±22.13 | 86.26±17.92 | <0.0001 |
| **WBC** | 9.99±6.04 | 7.33±3.46 | 12.52±6.82 | 0.0063 |
| **PLT** | 174.00±80.24 | 208.21±60.95 | 141.50±82.82 | 0.0085 |

Data are expressed as mean ± SEM, median, or percent.

BMI: Body mass index; ALT: glutamic-pyruvic transaminase; AST: glutamic oxalacetic transaminase; GGT: glutamyl transpeptidase; ALP: alkaline phosphatase; RBC: red blood cell; HGB: hemoglobin; WBC: white blood cell; PLT: blood platelet.

**Table S2 Baseline characteristics of patients with heart failure and non-heart failure.**

| Variables | Total(n=20) | Non-Heart failure (n=10) | Heart failure (n=10) | *P* value |
|---|---|---|---|---|
| **Age (years)** | 54.15±15.20 | 50.8±19.71 | 57.50±7.16 | 0.3505 |
| **Male gender (%)** | 14(70.00%) | 6(60.00%) | 8(80.00%) | 0.3290 |
| **BMI** | 23.45±4.22 | 22.84±3.58 | 24.06±4.70 | 0.5433 |
| **Smoking** | 9(45.00%) | 4(40.00%) | 5(50.00%) | 0.6530 |
| **Alcohol consumption** | 11(55.00%) | 5(50.00%) | 6(60.00%) | 0.6530 |
| **Medication history** | 9(45.00%) | 5(50.00%) | 4(40.00%) | 0.6530 |
| **SBP** | 120.95±17.30 | 120.70±21.18 | 121.20±12.24 | 0.9518 |
| **DBP** | 72.40±10.46 | 71.50±11.10 | 73.30±9.69 | 0.7182 |
| **NYHA Class** | 2.70±0.78 | 2.20±0.75 | 3.20±0.40 | 0.0024 |
| **Heart rate** | 83.95±11.99 | 85.70±15.94 | 82.20±5.25 | 0.5393 |
| **HFrEF** | 4(40.00%) | 0(0) | 4(40.00%) | 0.0870 |
| **LVEF** | 57.45±12.58 | 63.70±6.25 | 51.20±14.13 | 0.0259 |
| **LVIDd** | 9..68±2.01 | 10.15±2.43 | 9.21±1.32 | 0.3211 |
| **RVIDd** | 17.05±2.62 | 15.60±0.92 | 18.50±2.94 | 0.0112 |
| **LVEDd** | 48.80±9.48 | 43.30±4.17 | 54.30±10.10 | 0.0074 |
| **NT-proBNP** | 914.94±809.90 | 241.88±84.24 | 1475.00±685.03 | <0.0001 |
| **Albumin** | 38.43±4.88 | 39.21±4.92 | 37.65±4.72 | 0.5010 |
| **Sodium** | 139.20±3.28 | 140.30±3.38 | 138.10±2.77 | 0.1484 |
| **Inosinic acid** | 68.25±17.87 | 65.40±19.61 | 71.10±15.42 | 0.5018 |
| **Hemoglobin** | 118.74±34.42 | 113.38±42.38 | 124.10±22.72 | 0.5121 |
| **CHD** | 10(50.00%) | 4(40.00%) | 6(60.00%) | 0.1780 |
| **Hypertension** | 8(40.00%) | 4(40.00%) | 4(40.00%) | 1.0000 |
| **Pulmonary hypertension** | 2(10.00%) | 0(0) | 2(20.00%) | 0.1360 |
| **Heart failure duration (d)** | 194.75±488.83 | 0.00±0.00 | 389.50±634.08 | 0.0819 |
| **PCI** | 3(30.00%) | 0(0) | 3(30.00%) | 0.0600 |

Data are expressed as mean ± SEM, median, or percent.

BMI: Body mass index; SBP: Systolic blood pressure; DBP: Diastolic blood pressure; NYHA Class, The New York Heart Association functional classification; LVEF: Left ventricular ejection fraction; LVIDd: left ventricular internal dimension diastole; RVIDd: right ventricular internal dimension, diastole; LVEDd: left ventricular end diastolic diameter; CHD: Coronary artery disease; NT-proBNP, N-terminal pro-B type natriuretic peptide; PCI: percutaneous coronary intervention.

| Variables | Score and description |
|---|---|
| **Appearance** | 0- Coat is smooth |
| | 1- Patches of hair piloerected |
| | 2- Majority of back is piloerected |
| | 3- Piloerection may or may not be present, mouse appears "puffy" |
| | 4- Piloerection may or may not be present, mouse appears emaciated |
| **Level of consciousness** | 0- Mouse is active |
| | 1- Mouse is active but avoids standing upright |
| | 2- Mouse activity is noticeably slowed. The mouse is still ambulant. |
| | 3- Activity is impaired. Mouse only moves when provoked, movements have a tremor |
| | 4- Activity severely impaired. Mouse remains stationary when provoked, with possible tremor |
| **Activity** | 0- Normal amount of activity. Mouse is any of: eating, drinking, climbing, running, fighting |
| | 1- Slightly suppressed activity. Mouse is moving around bottom of cage |
| | 2- Suppressed activity. Mouse is stationary with occasional investigative movements |
| | 3- No activity. Mouse is stationary |
| | 4- No activity. Mouse experiencing tremors, particularly in the hind legs |
| **Response to stimulus** | 0- Mouse responds immediately to auditory stimulus or touch |
| | 1- Slow or no response to auditory stimulus; strong response to touch (moves to escape) |
| | 2- No response to auditory stimulus; moderate response to touch (moves a few steps) |
| | 3- No response to auditory stimulus; mild response to touch (no locomotion) |
| | 4- No response to auditory stimulus. Little or no response to touch. Cannot right itself if pushed over |
| **Eyes** | 0- Open |
| | 1- Eyes not fully open, possibly with secretions |
| | 2- Eyes at least half closed, possibly with secretions |
| | 3- Eyes half closed or more, possibly with secretions |
| | 4- Eyes closed or milky |
| **Respiration rate** | 0- Normal, rapid mouse respiration |
| | 1- Slightly decreased respiration (rate not quantifiable by eye) |
| | 2- Moderately reduced respiration (rate at the upper range of quantifying by eye) |
| | 3- Severely reduced respiration (rate easily countable by eye, 0.5 s between breaths) |
| | 4- Extremely reduced respiration (>1 s between breaths) |
| **Respiration quality** | 0- Normal |
| | 1- Brief periods of laboured breathing |
| | 2- Laboured, no gasping |
| | 3- Laboured with intermittent gasps |
| | 4- Gasping |

**Table S3 Murine sepsis score (MSS)**

**Table S4 Information about correlation immunohistochemical antibody**

| Antibodies | Concentration | Product No | Manufacturers |
|---|---|---|---|
| anti-GAS6 | 1:200 | bs-7549R | Bioss Biotechnology Co., Ltd, Beijing, China |
| anti-AXL | 1:200 | bs-5180R | Bioss Biotechnology Co., Ltd, Beijing, China |
| anti-NLRP3 | 1:200 | bs-10021R | Bioss Biotechnology Co., Ltd, Beijing, China |
| anti-IL-6 | 1:200 | GB11117 | Servicebio, Wuhan, Hubei, China |
| anti-MPO | 1:200 | 22225-1-AP | Proteintech, Wuhan, China |
| anti-F4/80 | 1:200 | GB11027 | Servicebio, Wuhan, Hubei, China |
| anti-Ly6G | 1:200 | GB11229 | Servicebio, Wuhan, Hubei, China |
| anti-TNF-α | 1:200 | GB11188 | Servicebio, Wuhan, Hubei, China |

**Table S5 Information about correlation primers**

| Primer | Sequences |
|---|---|
| mouse-GAS6 | F：5‘-TACCTACAGGCTCAACTACACC-3’ |
| | R：5‘-CTCAACTGCCAGGACCAC-3’ |
| mouse-AXL | F：5‘-ATGGCCGACATTGCCAGTG-3’ |
| | R：5‘-CGGTAGTAATCCCCGTTGTAGA-3’ |
| mouse-NLRP3 | F：5‘-ACCTCAACAGTCGCTACAC-3’ |
| | R：5‘-GTCCTCGGGCTCAAACA-3’ |
| mouse-Caspase-1 | F：5‘-AGAACAGAACAAAGAAGATGGCACA-3’ |
| | R：5‘-GTGCCATCTTCTTTGTTCTGTTCTT-3’ |
| mouse-IL-1β | F：5‘-TGGACCTTCCAGGATGAGGACA-3’ |
| | R：5‘-GTTCATCTCGGAGCCTGTAGTG-3’ |
| mouse-IL-6 | F：5‘-TACCACTTCACAAGTCGGAGGC-3’ |
| | R：5‘-CTGCAAGTGCATCATCGTTGTT-3’ |
| mouse-TNF-α | F：5‘-GGTGCCTATGTCTCAGCCTCTT-3’ |
| | R：5‘-GCCATAGAACTGATGAGAGGGAG-3’ |
| mouse-β-actin | F：5‘-CTTTTCCAGCCTTCCTTCTT-3’ |
| | R：5‘-GGTCTTTACGGATGTCAACG-3’ |
| rat-GAS6 | F：5‘-AAACGGTCAAGGCCAATACA -3’ |
| | R：5‘-ATGCGAGCCACGACTTCTAC-3’ |
| rat-AXL | F：5‘-ATCGGAGGAAGAAGGAGACG-3’ |
| | R：5‘-TGCCCAGACTGTTCAAGGTG-3’ |
| rat-NLRP3 | F：5‘-CAGAAGCTGGGGTTGGTGAA-3’ |
| | R：5‘-CCCATGTCTCCAAGGGCATT-3’ |
| rat-Caspase-1 | F：5‘-CTGGAGCTTCAGTCAGGTCC-3’ |
| | R：5‘-CTTGAGGGAACCACTCGGTC-3’ |
| rat-IL-1β | F：5‘-AACTGTGAAATAGCAGCTTTCG-3’ |
| | R：5‘-CTGTGAGATTTGAAGCTGGATG-3’ |
| rat-IL-6 | F：5‘-TCCGGAGAGGAGACTTCACA-3’ |
| | R：5‘-TGCCATTGCACAACTCTTTTCT-3’ |
| rat-TNF-α | F：5‘-ACTGAACTTCGGGGTGATCG-3’ |
| | R：5‘-TGGTGGTTTGCTACGACGTG-3’ |
| rat-β-actin | F：5‘-AGAGCTATGAGCTGCCTGAC -3’ |
| | R：5‘-AATTGAATGTAGTTTCATGGATG-3’ |

**Table S6 Information about antibodies used in Western blot**

| Antibodies | Concentration | Product No | Manufacturers |
|---|---|---|---|
| anti-GAS6 | 1:1000 | bs-7549R | Bioss Biotechnology Co., Ltd, Beijing, China |
| anti-AXL | 1:1000 | bs-5180R | Bioss Biotechnology Co., Ltd, Beijing, China |
| anti-NLRP3 | 1:1000 | bs-10021R | Bioss Biotechnology Co., Ltd, Beijing, China |
| anti-Caspase-1 | 1:1000 | GB11383 | Servicebio, Wuhan, Hubei, China |
| anti-IL-1 | 1:1000 | bs-0812R | Bioss Biotechnology Co., Ltd, Beijing, China |
| anti-ASC | 1:500 | sc-514414 | Santa Cruz Biotechnology, Dallas, TX, USA |
| anti-IL-6 | 1:500 | sc-57315 | Santa Cruz Biotechnology, Dallas, TX, USA |
| anti-Ly6G | 1:1000 | bs-2576R | Bioss Biotechnology Co., Ltd, Beijing, China |
| anti-Ly6C | 1:1000 | A33313 | Boster Biological Technology Co., Ltd, Wuhan, Hubei, China |
| anti-BAX | 1:1000 | 2772s | Cell Signaling Technology, Inc, Danvers, Massachusetts, USA |
| anti-Bcl2 | 1:1000 | BA0412 | Boster Biological Technology Co., Ltd, Wuhan, Hubei, China |
| anti-Caspase-3 | 1:500 | sc-56053 | Santa Cruz Biotechnology, Dallas, TX, USA |
| anti-Nrf2 | 1:1000 | PB9290 | Boster Biological Technology Co., Ltd, Wuhan, Hubei, China |
| anti-HO-1 | 1:1000 | ab189491 | Abcam Biotechnology, Cambridge, United Kingdom |
| anti-NQO1 | 1:500 | sc-393736 | Santa Cruz Biotechnology, Dallas, TX, USA |
| anti-ATF6 | 1:500 | sc-166659 | Santa Cruz Biotechnology, Dallas, TX, USA |
| anti-GRP78 | 1:1000 | 3177s | Cell Signaling Technology, Inc, Danvers, Massachusetts, USA |
| anti-p-PERK | 1:1000 | bs-23340R | Bioss Biotechnology Co., Ltd, Beijing, China |
| anti-PERK | 1:1000 | 3192s | Cell Signaling Technology, Inc, Danvers, Massachusetts, USA |
| anti-CHOP | 1:1000 | A00311-2 | Boster Biological Technology Co., Ltd, Wuhan, Hubei, China |
| anti-NRF1 | 1:500 | sc-101102 | Santa Cruz Biotechnology, Dallas, TX, USA |
| anti-UCP2 | 1:1000 | GB11377 | Servicebio, Wuhan, Hubei, China |
| anti-TFAM | 1:500 | sc-166965 | Santa Cruz Biotechnology, Dallas, TX, USA |
| anti-GAPDH | 1:1000 | GB11002 | Servicebio, Wuhan, Hubei, China |
| anti-β-actin | 1:1000 | GB11001 | Servicebio Technology Co., Ltd, Wuhan, China |
| Goat anti-mouse IgG | 1:5000 | 7076s | Cell Signaling Technology, Inc., Danvers, Massachusetts, USA |
| Goat anti-rabbit IgG | 1:5000 | BA1054 | Boster Biological Technology Co., Ltd., CA, USA |

**Table S7 Information about FDA-approved drugs library used in this study**

| No | Barcode | Catalog Number | Product Name | CAS Number | M.w. | Significant Toxicity (10 μM) |
|---|---|---|---|---|---|---|
| 1A1 | 54416774 | S1022 | Ridaforolimus (Deforolimus, MK-8669) | 572924-54-0 | 990.21 | NO |
| 1B1 | 46002394 | S1029 | Lenalidomide (CC-5013) | 191732-72-6 | 259.26 | NO |
| 1C1 | 55195245 | S1039 | Rapamycin (Sirolimus) | 53123-88-9 | 914.18 | NO |
| 1D1 | 55191625 | S1040 | Sorafenib Tosylate | 475207-59-1 | 637.03 | NO |
| 1E1 | 55449921 | S1044 | Temsirolimus (CCI-779, NSC 683864) | 162635-04-3 | 1030.29 | NO |
| 1F1 | 54412381 | S1119 | Cabozantinib (XL184, BMS-907351) | 849217-68-1 | 501.51 | NO |
| 1G1 | 55210717 | S1120 | Everolimus (RAD001) | 159351-69-6 | 958.22 | NO |
| 1H1 | 45997586 | S1193 | Thalidomide | 50-35-1 | 258.23 | NO |
| 1A2 | 54527905 | S1267 | Vemurafenib (PLX4032, RG7204) | 918504-65-1 | 489.92 | NO |
| 1B2 | 55420235 | S1322 | Dexamethasone (DHAP) | 50-02-2 | 392.46 | NO |
| 1C2 | 45917691 | S1378 | Ruxolitinib (INCB018424) | 941678-49-5 | 306.37 | NO |
| 1D2 | 54408717 | S1458 | VX-745 | 209410-46-8 | 436.26 | NO |
| 1E2 | 55206536 | S1466 | Calcitriol | 32222-06-3 | 416.64 | NO |
| 1F2 | 55199021 | S1467 | Doxercalciferol | 54573-75-0 | 412.65 | NO |
| 1G2 | 55193428 | S1468 | Alfacalcidol | 41294-56-8 | 400.64 | NO |
| 1H2 | 54469915 | S1491 | Fludarabine | 21679-14-1 | 285.23 | NO |
| 1A3 | 45529212 | S1567 | Pomalidomide | 19171-19-8 | 273.24 | NO |
| 1B3 | 55203466 | S1623 | Acetylcysteine | 616-91-1 | 163.19 | NO |
| 1C3 | 54491197 | S1652 | Monobenzone | 103-16-2 | 200.23 | NO |
| 1D3 | 45529577 | S1655 | Ezetimibe | 163222-33-1 | 409.40 | NO |
| 1E3 | 46102129 | S1721 | Azathioprine | 446-86-6 | 277.26 | NO |
| 1F3 | 54363749 | S1744 | Nicotinic Acid | 59-67-6 | 123.11 | NO |
| 1G3 | 54404701 | S1833 | Butoconazole nitrate | 64872-77-1 | 474.79 | NO |
| 1H3 | 55471402 | S1848 | Curcumin | 458-37-7 | 368.38 | NO |
| 1A4 | 46489757 | S1902 | Vitamin B12 | 68-19-9 | 1355.37 | NO |
| 1B4 | 55416552 | S1921 | Phenindione | 83-12-5 | 222.24 | NO |
| 1C4 | 55578687 | S1949 | Menadione | 58-27-5 | 172.18 | NO |
| 1D4 | 46484393 | S2015 | Suplatast Tosylate | 94055-76-2 | 499.64 | NO |
| 1E4 | 54422743 | S2119 | Probucol | 23288-49-5 | 516.84 | NO |
| 1F4 | 55418076 | S2226 | Idelalisib (CAL-101, GS-1101) | 870281-82-6 | 415.42 | NO |
| 1G4 | 55199109 | S2233 | Esomeprazole sodium | 161796-78-7 | 367.40 | NO |
| 1H4 | 46048734 | S2261 | Andrographolide | 5508-58-7 | 350.45 | NO |
| 1A5 | 55203563 | S2263 | Arbutin | 497-76-7 | 272.25 | NO |
| 1B5 | 54408494 | S2265 | Artesunate | 88495-63-0 | 384.42 | NO |
| 1C5 | 55585080 | S2310 | Honokiol | 35354-74-6 | 266.33 | NO |
| 1D5 | 46084158 | S2321 | Magnolol | 528-43-8 | 266.33 | NO |
| 1E5 | 55555229 | S2341 | (-)-Parthenolide | 20554-84-1 | 248.32 | NO |

| | | | | | | |
|---|---|---|---|---|---|---|
| 1F5 | 55460561 | S2375 | Aloin | 1415-73-2 | 418.39 | NO |
| 1G5 | 55460533 | S2391 | Quercetin | 117-39-5 | 302.24 | NO |
| 1H5 | 54493851 | S2528 | Ciclopirox | 29342-05-0 | 207.27 | NO |
| 1A6 | 55187768 | S2673 | Trametinib (GSK1120212) | 871700-17-3 | 615.39 | NO |
| 1B6 | 46050809 | S2789 | Tofacitinib (CP-690550,Tasocitinib) | 477600-75-2 | 312.37 | NO |
| 1C6 | 46077641 | S2807 | Dabrafenib (GSK2118436) | 1195765-45-7 | 519.56 | NO |
| 1D6 | 55199049 | S2814 | Alpelisib (BYL719) | 1217486-61-7 | 441.47 | NO |
| 1E6 | 55209289 | S2851 | Baricitinib (LY3009104, INCB028050) | 1187594-09-7 | 371.42 | NO |
| 1F6 | 55508075 | S2902 | S-Ruxolitinib (INCB018424) | 941685-37-6 | 306.37 | NO |
| 1G6 | 55472772 | S3019 | Ciclopirox ethanolamine | 41621-49-2 | 268.35 | NO |
| 1H6 | 54437559 | S3023 | Bufexamac | 2438-72-4 | 223.27 | NO |
| 1A7 | 46083002 | S3113 | Pyridoxine HCl | 58-56-0 | 205.64 | NO |
| 1B7 | 45499662 | S3114 | Vitamin C | 50-81-7 | 176.12 | NO |
| 1C7 | 54469834 | S3124 | Dexamethasone Acetate | 1177-87-3 | 434.50 | NO |
| 1D7 | 55464316 | S3130 | Biotin (Vitamin B7) | 58-85-5 | 244.31 | NO |
| 1E7 | 46229537 | S3137 | Sodium salicylate | 54-21-7 | 161.11 | NO |
| 1F7 | 55475528 | S3604 | Triptolide (PG490) | 38748-32-2 | 360.40 | NO |
| 1G7 | 55578642 | S3681 | Vitamin E Acetate | 58-95-7 | 472.74 | NO |
| 1H7 | 55452693 | S3739 | Calcipotriene | 112965-21-6 | 412.60 | NO |
| 1A8 | 45823293 | S3980 | Pyridoxine | 65-23-6 | 169.18 | NO |
| 1B8 | 55535567 | S4001 | Cabozantinib malate (XL184) | 1140909-48-3 | 635.59 | NO |
| 1C8 | 43999908 | S4063 | Cholecalciferol (Vitamin D3) | 67-97-0 | 384.64 | NO |
| 1D8 | 54425069 | S4083 | Vitamin A Acetate | 127-47-9 | 328.49 | NO |
| 1E8 | 54530574 | S4163 | Doxycycline Hyclate | 24390-14-5 | 512.94 | NO |
| 1F8 | 46203086 | S4182 | Nifuroxazide | 965-52-6 | 275.22 | NO |
| 1G8 | 56053831 | S4238 | Cepharanthine | 481-49-2 | 606.71 | NO |
| 1H8 | 54410212 | S4267 | Diacerein | 13739-02-1 | 368.29 | NO |
| 1A9 | 55207745 | S4299 | Dicoumarol | 66-76-2 | 336.29 | NO |
| 1B9 | 55209901 | S4571 | Hexylresorcinol | 136-77-6 | 194.27 | NO |
| 1C9 | 54409767 | S4695 | D panthenol | 81-13-0 | 205.25 | NO |
| 1D9 | 54440374 | S4698 | Vitamin K1 | 84-80-0 | 450.70 | NO |
| 1E9 | 46213671 | S4779 | Menadiol Diacetate | 573-20-6 | 258.27 | NO |
| 1F9 | 47036060 | S4871 | Pyridoxal 5-phosphate monohydrate | 41468-25-1 | 265.16 | NO |
| 1G9 | 55521125 | S4994 | Methylcobalamin | 13422-55-4 | 1344.38 | NO |
| 1H9 | 46248800 | S5001 | Tofacitinib (CP-690550) Citrate | 540737-29-9 | 504.49 | NO |
| 1A10 | 47463042 | S5003 | Tacrolimus (FK506) | 104987-11-3 | 804.02 | NO |
| 1B10 | 54430655 | S5069 | Dabrafenib Mesylate | 1195768-06-9 | 615.67 | NO |
| 1C10 | 55426564 | S5077 | Regorafenib Monohydrate | 1019206-88-2 | 500.83 | NO |
| 1D10 | 54414606 | S5082 | Vitamin K2 | 863-61-6 | 444.65 | NO |
| 1E10 | 55539164 | S5103 | Lutein | 127-40-2 | 568.87 | NO |
| 1F10 | 47020953 | S5174 | Kojic acid | 501-30-4 | 142.11 | NO |
| 1G10 | 55557110 | S5243 | Ruxolitinib Phosphate | 1092939-17-7 | 404.36 | NO |
| 1H10 | 55556796 | S5311 | Pyridoxal phosphate | 54-47-7 | 247.14 | NO |
| 2A1 | 54418281 | S5454 | Saikosaponin D | 20874-52-6 | 780.98 | YES |

| | | | | | | |
|---|---|---|---|---|---|---|
| 2B1 | 55546081 | S5466 | Saikosaponin A | 20736-09-8 | 780.98 | NO |
| 2C1 | 55413994 | S5550 | Ethyl gallate | 831-61-8 | 198.17 | NO |
| 2D1 | 55537861 | S5554 | Lanatoside C | 17575-22-3 | 985.12 | NO |
| 2E1 | 55219634 | S5558 | D-Pantothenate Sodium | 867-81-2 | 241.22 | NO |
| 2F1 | 55545008 | S5592 | Vitamin A | 68-26-8 | 286.45 | NO |
| 2G1 | 55585094 | S5733 | Stearic acid | 57-11-4 | 284.48 | YES |
| 2H1 | 54525112 | S5754 | Baricitinib phosphate | 1187595-84-1 | 469.41 | NO |
| 2A2 | 56024802 | S6104 | (±)-α-Tocopherol | 10191-41-0 | 430.71 | NO |
| 2B2 | 55424985 | S7007 | Binimetinib (MEK162, ARRY-162, ARRY-438162) | 606143-89-9 | 441.23 | NO |
| 2C2 | 55416799 | S7028 | Duvelisib (IPI-145, INK1197) | 1201438-56-3 | 416.86 | NO |
| 2D2 | 55428041 | S7091 | Zotarolimus(ABT-578) | 221877-54-9 | 966.21 | NO |
| 2E2 | 55557687 | S7156 | Marimastat (BB-2516) | 154039-60-8 | 331.41 | NO |
| 2F2 | 55436559 | S7397 | Sorafenib | 284461-73-0 | 464.82 | NO |
| 2G2 | 55537479 | S7650 | Peficitinib (ASP015K, JNJ-54781532) | 944118-01-8 | 326.39 | NO |
| 2H2 | 55582498 | S7754 | Gilteritinib (ASP2215) | 1254053-43-4 | 552.71 | NO |
| 2A3 | 55528173 | S8034 | Apremilast (CC-10004) | 608141-41-9 | 460.50 | NO |
| 2B3 | 54445994 | S8041 | Cobimetinib (GDC-0973, RG7420) | 934660-93-2 | 531.31 | NO |
| 2C3 | 46458297 | S8048 | Venetoclax (ABT-199, GDC-0199) | 1257044-40-8 | 868.44 | YES |
| 2D3 | 43855361 | S8101 | CB-5083 | 1542705-92-9 | 413.47 | NO |
| 2E3 | 43999406 | S8133 | Resiquimod | 144875-48-9 | 314.38 | NO |
| 2F3 | 55577587 | S8195 | Oclacitinib maleate | 1640292-55-2 | 453.51 | NO |
| 2G3 | 55567312 | S9042 | Wedelolactone | 524-12-9 | 314.25 | NO |
| 2H3 | 55575179 | S9354 | Oxalic acid | 144-62-7 | 90.03 | NO |
| 2A4 | 55511276 | S2736 | Fedratinib (SAR302503, TG101348) | 936091-26-8 | 524.68 | NO |
| 2B4 | 56055641 | S9015 | Homoharringtonine | 26833-87-4 | 545.62 | NO |
| 2C4 | 55224606 | S9102 | Magnolin | 31008-18-1 | 416.46 | NO |
| 2D4 | 56015413 | S2188 | Phenprocoumon | 435-97-2,53621-47-9 (sodium salt) | 280.32 | NO |
| 2E4 | 55473308 | S2382 | Evodiamine | 518-17-2 | 303.36 | NO |
| 2F4 | 55466628 | S3030 | Niclosamide | 50-65-7 | 327.12 | NO |
| 2G4 | 54400147 | S6614 | Fursultiamine | 804-30-8 | 398.54 | NO |
| 2H4 | 46096943 | S4073 | Sodium 4-Aminosalicylate | 6018-19-5 | 211.15 | NO |
| 2A5 | 55212494 | S1037 | Perifosine (KRX-0401) | 157716-52-4 | 461.66 | NO |
| 2B5 | 55548727 | S3056 | Miltefosine | 58066-85-6 | 407.57 | NO |
| 2C5 | 49534014 | S3211 | Thiamine HCl (Vitamin B1) | 67-03-8 | 337.27 | NO |
| 2D5 | 55458282 | S4028 | Dexamethasone Sodium Phosphate | 55203-24-2 | 516.40 | NO |
| 2E5 | 55513375 | S4144 | Amprolium HCl | 137-88-2 | 315.24 | NO |
| 2F5 | 55508459 | S4245 | Sodium ascorbate | 134-03-2 | 201.13 | NO |
| 2G5 | 55543552 | S4811 | VitaMin U | 3493-12-7 | 199.70 | NO |
| 2H5 | 45486111 | S5220 | D-Pantethine | 16816-67-4 | 554.72 | NO |
| 2A6 | 55467066 | S1767 | Beta Carotene | 7235-40-7 | 536.87 | NO |
| 2B6 | 54433849 | S4126 | Retinyl (Vitamin A) Palmitate | 79-81-2 | 524.86 | NO |
| 2C6 | 55214202 | S4605 | Folic acid | 59-30-3 | 441.40 | NO |

## SUPPLEMENTAL FIGURES

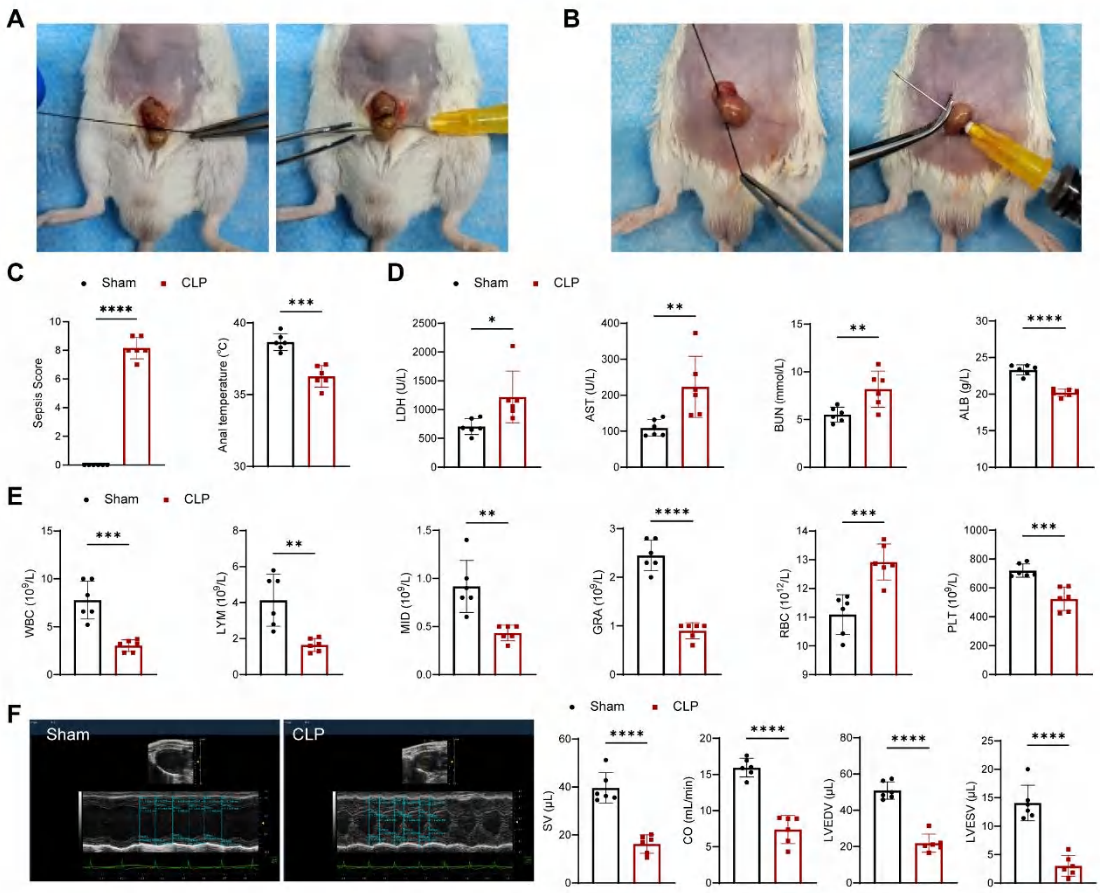


**Fig. S1 The construction of sepsis models *in vivo*. (A)** Construction of CLP model for functional experiments (left: cecal ligation; right: cecal perforation); **(B)** Construction of aggravated CLP model for survival analysis (left: cecal ligation; right: cecal perforation); **(C)** The sepsis score and anal temperature in mice 8 h following CLP; **(D)** Blood biochemical parameters in mice 8 h following CLP; **(E)** Blood routine parameters in mice 8 h following CLP; **(F)** Representative and quantified (CO, SV, LVEDV and LVESV) echocardiographic results of the long axis in mice 8 h following CLP. n=6 per group, Mean ± SD. $^{*}P$<0.05, $^{**}P$<0.01, $^{***}P$<0.001 vs. Sham.

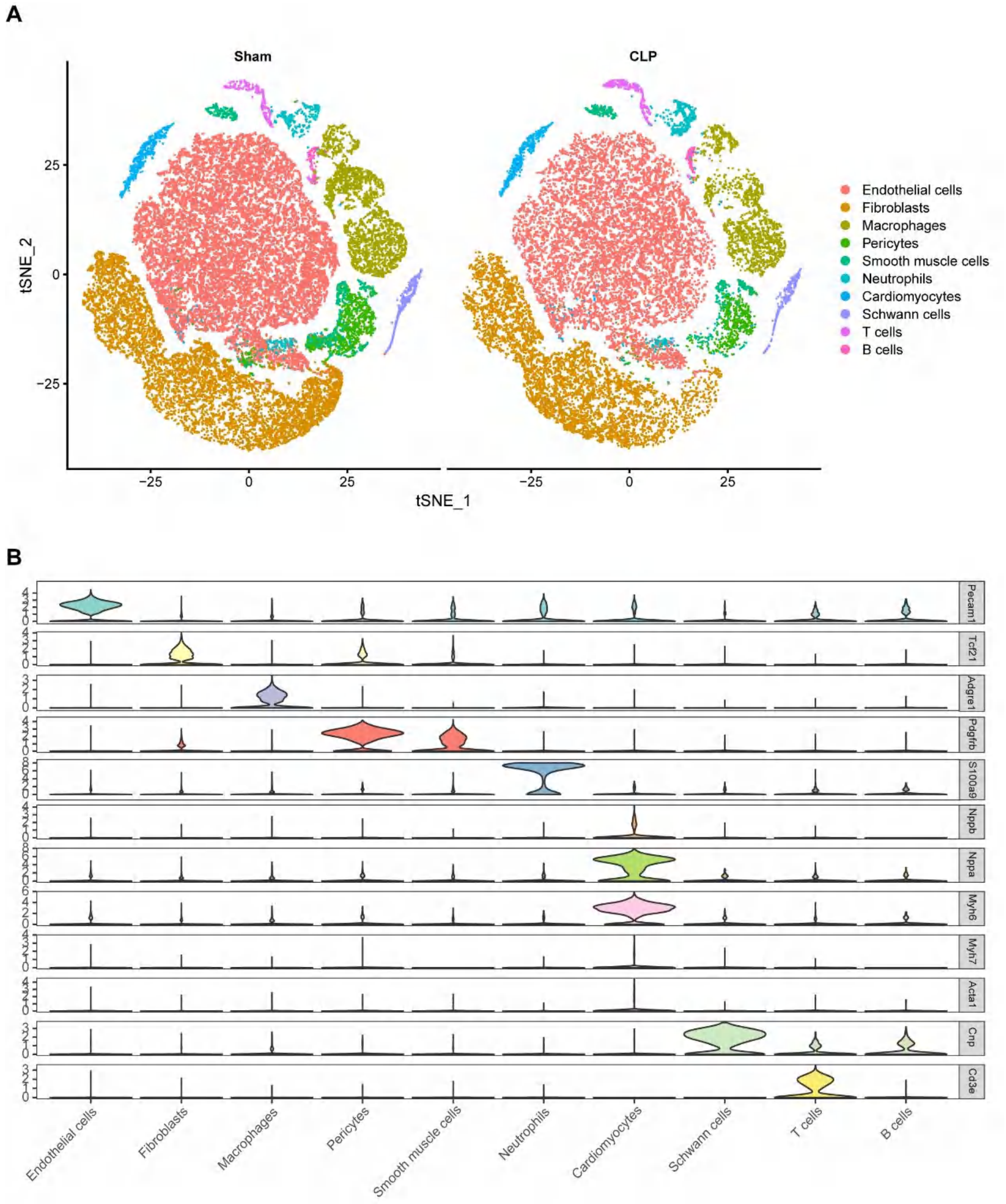


**Fig. S2 Supplemental data of Single-cell RNA sequencing in cell clusters of heart tissues. (A)** t-distributed Stochastic Neighbor Embedding (t-SNE) visualization of nuclei from hearts of 3 Sham mice and 3 CLP mice. Cells are color-coded according to their different types; **(B)** Violin plots showing single-cell normalized expression of the most discriminative surface markers in the 10 cell populations.

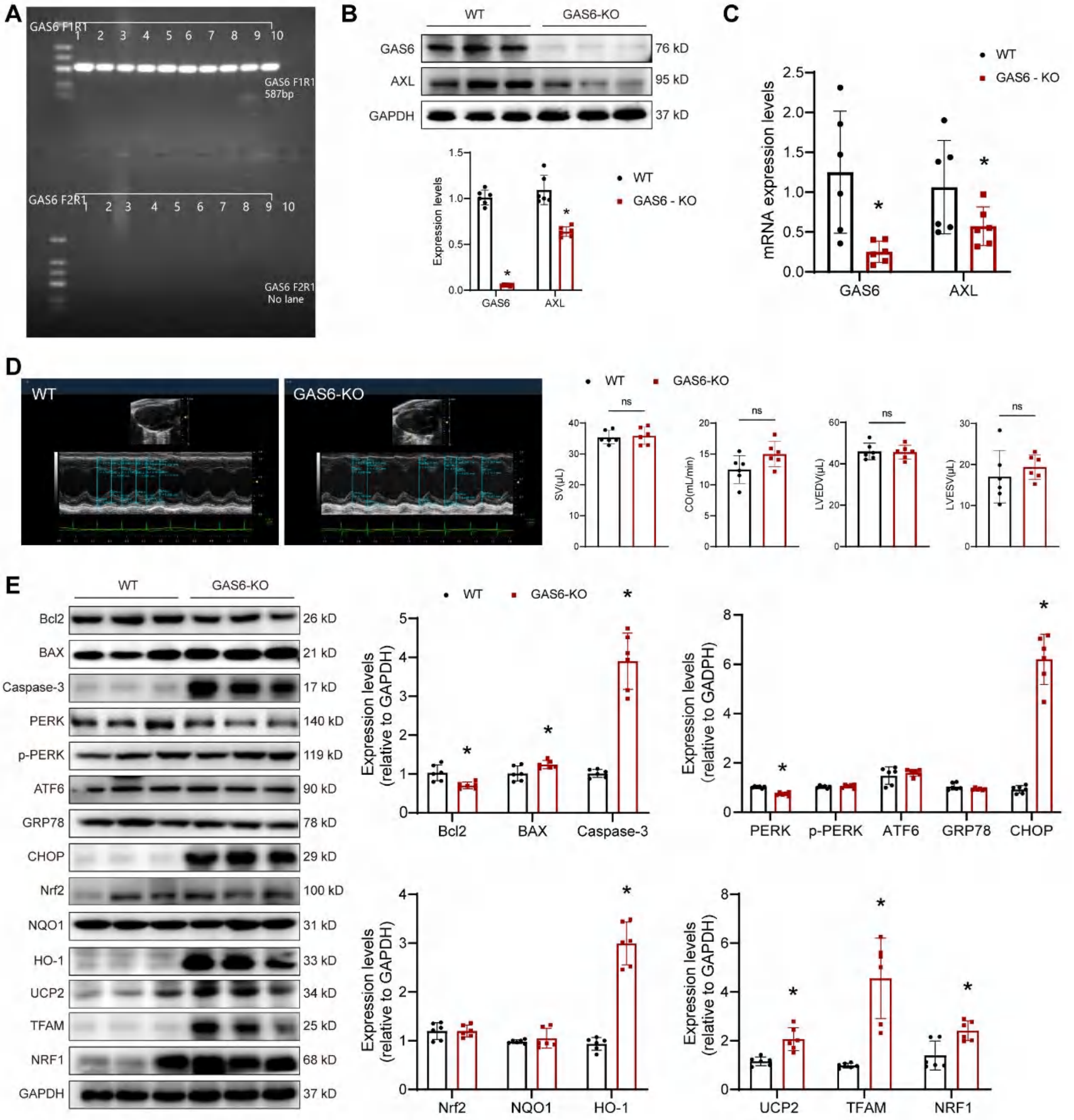


**Fig. S3 The effects of GAS6 deficiency alone *in vivo* on CLP-free mice. (A)** Southern blot images in mice with GAS6 deficiency, n=10; **(B)** Representative and pooled Western blot analysis of myocardial GAS6 and AXL in mice with GAS6 deficiency. n=6; **(C)** qRT-PCR analysis of myocardial *GAS6* and *AXL* in mice with GAS6 deficiency, n=6; **(D)** Representative and quantified (CO, SV, LVEDV and LVESV) echocardiographic results of the long axis in mice with GAS6 deficiency, n=6; and **(E)** Representative and pooled Western blot analysis of myocardial Bcl2, BAX, Caspase-3, PERK, p-PERK, ATF6, GRP78, CHOP, Nrf2, NQO1, HO-1, UCP2, TFAM and NRF1 in mice with GAS6 deficiency, n=6. Mean ± SD, $^{*}P$<0.05 vs. WT.

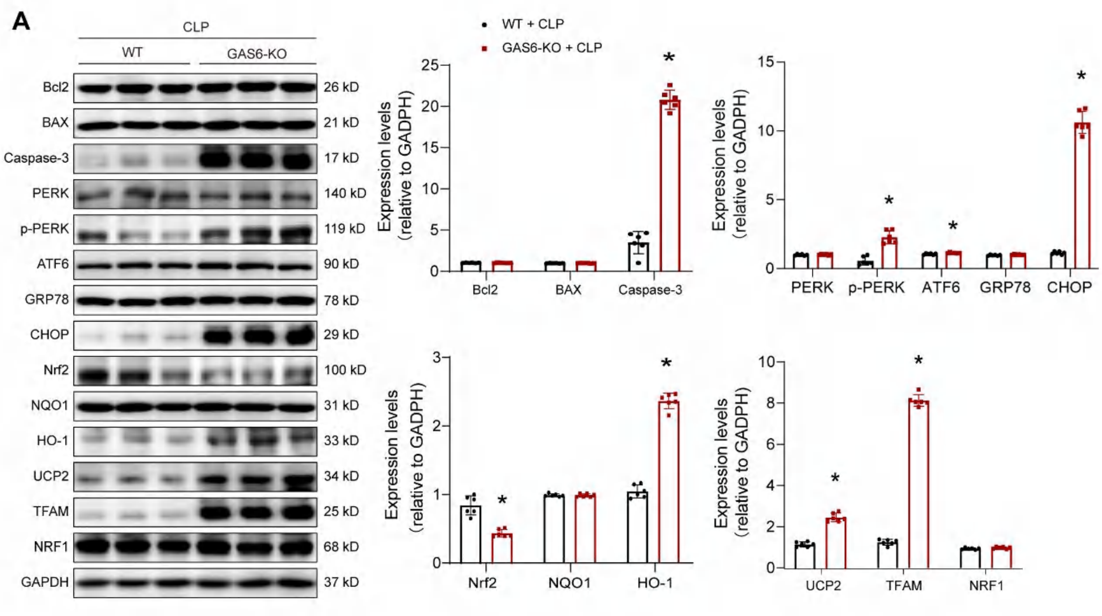


**Fig. S4 The effects of GAS6 knockout *in vivo* on CLP-induced various pathways. (A)** Representative and pooled Western blot analysis of myocardial Bcl2, BAX, Caspase-3, PERK, p-PERK, ATF6, GRP78, CHOP, Nrf2, NQO1, HO-1, UCP2, TFAM and NRF1 in CLP-injured mice with GAS6-KO. Mean ± SD. n=6 per group, $^{*}P<0.05$. $^{**}P<0.01$, $^{***}P<0.001$, vs. WT-CLP.

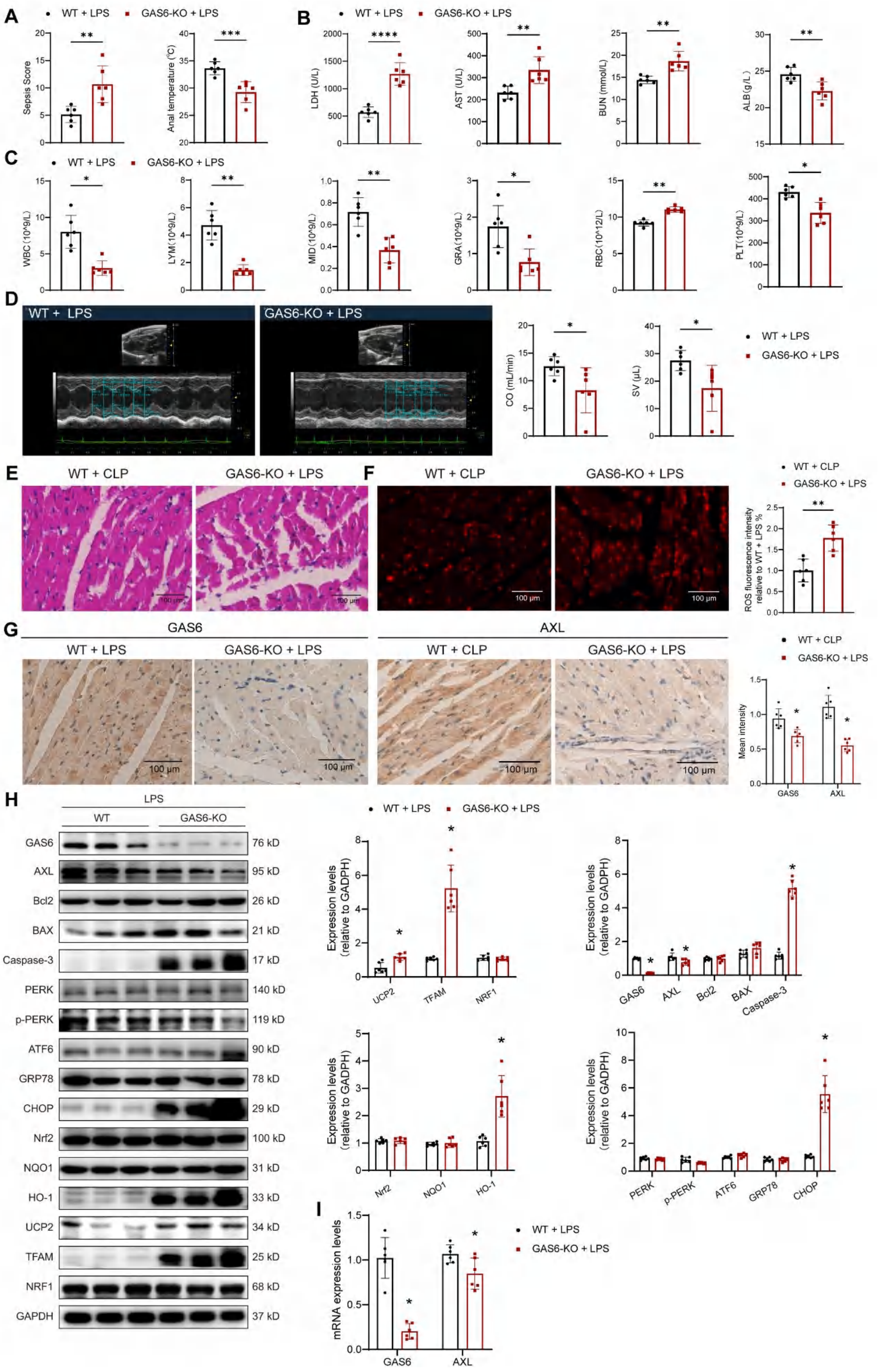


**Fig. S5 GAS6 deficiency evoked deterioration of systemic biometrics, cardiac structure and**

**function in LPS mice. (A)** The sepsis score and anal temperature in mice 8 h following LPS; **(B)** Blood biochemical parameters in mice 8 h following LPS; **(C)** Blood routine parameters in mice 8 h following LPS; Representative and quantified (CO and SV) echocardiographic results of the long **(D)** axis in mice 8 h following LPS; **(E)** Representative images of H&E staining in myocardia 8 h following LPS； **(F)** Representative images of DHE staining and quantitative analysis in myocardia 8 h following LPS; **(G)** Representative images of GAS6 and AXL IHC staining, and quantitative analysis of IHC staining in myocardia 8 h following LPS； **(H)** Representative and pooled Western blot analysis of myocardial GAS6, AXL, Bcl2, BAX, Caspase-3, PERK, p-PERK, ATF6, GRP78, CHOP, Nrf2, NQO1, HO-1, UCP2, TFAM and NRF1 in mice 8 h after LPS; and **(I)** qRT-PCR analysis of myocardial *GAS6* and *AXL* mRNA in mice 8 h after LPS. Mean ± SD. n=6 per group. $^{*}P<0.05$, $^{**}P<0.01$, $^{***}P<0.001$ vs. WT-LPS.

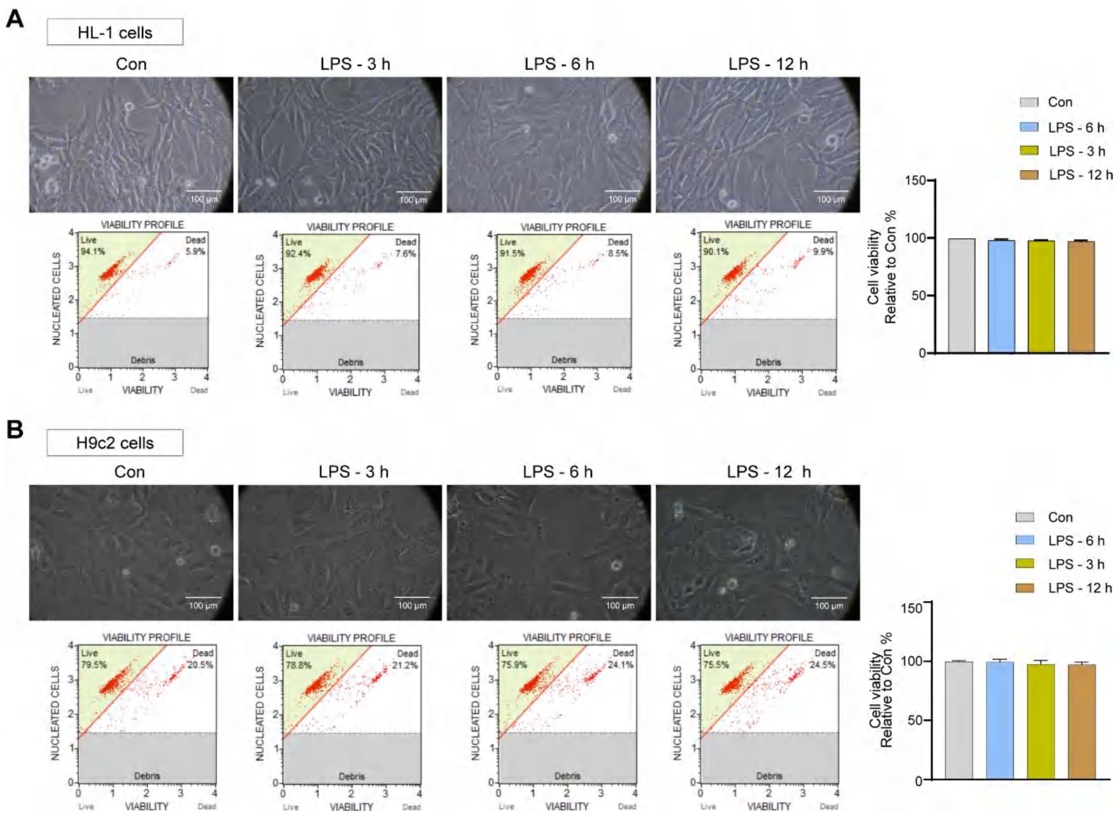


**Fig. S6 The construction of sepsis models *in vitro*. (A)** Cell viability in HL-1 cells 3, 6, and 12 h following LPS stimulation; and **(B)** Cell viability in H9c2 cells 3, 6, and 12 h following LPS stimulation. n=6 per group, Mean ± SD. $^{*}P$<0.05, $^{**}P$<0.01, $^{***}P$<0.001 vs. Con.

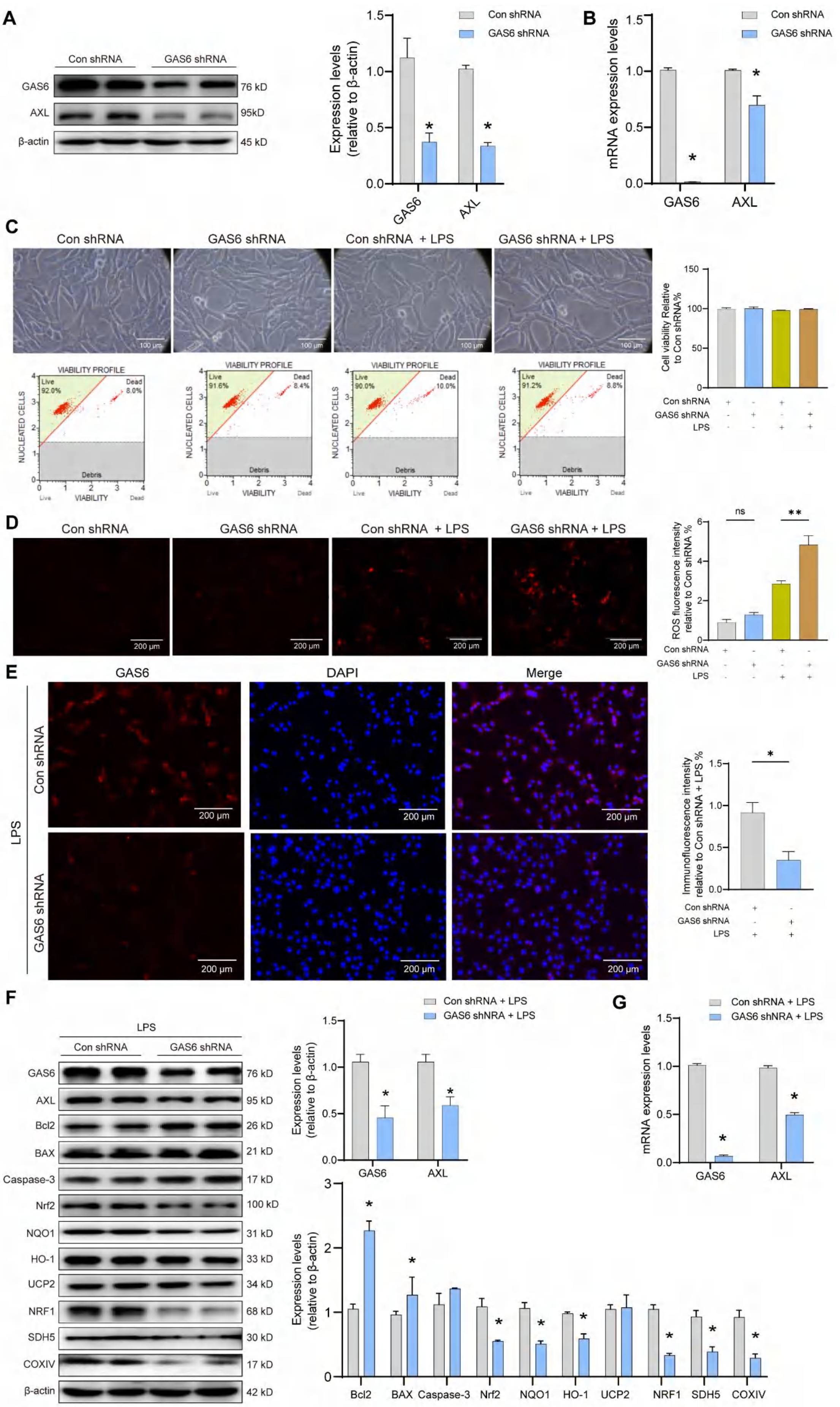

A
Con shRNA
GAS6 shRNA
GAS6
AXL
β-actin
76 kD
95kD
45 kD
Expression levels (relative to β-actin)
B
mRNA expression levels
C
Con shRNA
GAS6 shRNA
Con shRNA + LPS
GAS6 shRNA + LPS
VIABILITY PROFILE
NUCLEATED CELLS
VIABILITY
Live
Dead
Debris
Cell viability Relative to Con shRNA%
LPS
D
ROS fluorescence intensity relative to Con shRNA %
ns
E
DAPI
Merge
Immunofluorescence intensity relative to Con shRNA + LPS %
F
Bcl2
BAX
Caspase-3
Nrf2
NQO1
HO-1
UCP2
NRF1
SDH5
COXIV
G

**Fig. S7 GAS6 deficiency aggravated LPS injury in HL-1 cells**. **(A)** Representative and pooled Western blot analysis of GAS6 and AXL in HL-1 cells with GAS6 shRNA; **(B)** qRT-PCR analysis of *GAS6* and *AXL* mRNA in HL-1 cells with GAS6 shRNA; (**C**) Representative cell morphology images and cell viability of LPS-stimulated HL-1 cells with GAS6 deficiency; (**D**) Intracellular ROS levels of LPS-stimulated HL-1 cells with GAS6 deficiency; (**E**) Representative and pooled immunofluorescence photographs for GAS6 (red) and DAPI (blue) in LPS-stimulated HL-1 cells with GAS6 deficiency; (**F**) Representative and pooled Western blot analysis of GAS6, AXL, Bcl2, BAX, Caspase-3, Nrf2, NQO1, HO-1, UCP2, NRF1, SDH5 and COXIV following LPS-stimulated in HL-1 cells with GAS6 deficiency; and (**G**) qRT-PCR analysis of GAS6 and AXL mRNA following LPS-stimulated in HL-1 cells with GAS6 deficiency. Mean ± SD. n=6 per group, *$P$<0.05, **$P$<0.01, ***$P$<0.001 vs. Con shRNA (panel A-B) or Con shRNA-LPS (panel C-F).

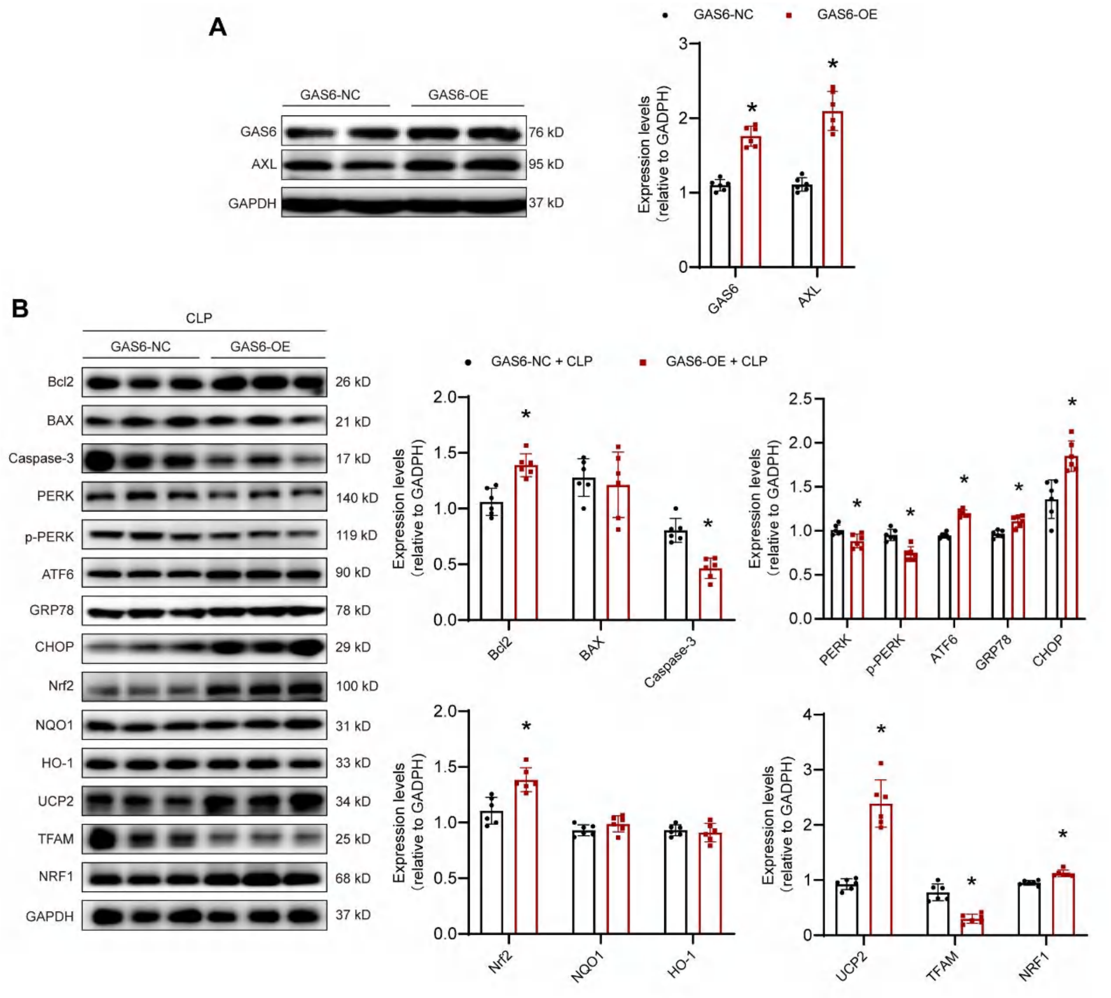


**Fig. S8 Identification of GAS6 overexpression in mice and the effects of GAS6 overexpression *in vivo* on the CLP-induced various pathways. (A)** Representative and pooled Western blot analysis of myocardial GAS6 and AXL in mice with GAS6-OE; and **(B)** Representative and pooled Western blot analysis of myocardial Bcl2, BAX, Caspase-3, PERK, p-PERK, ATF6, GRP78, CHOP, Nrf2, NQO1, HO-1, UCP2, TFAM and NRF1 in CLP-injured mice with GAS6-OE. Mean ± SD. n=6 per group, $^{*}P$<0.05, $^{**}P$<0.01, $^{***}P$<0.001 vs. GAS6-NC (panel A) or GAS6-NC-CLP (panel B).

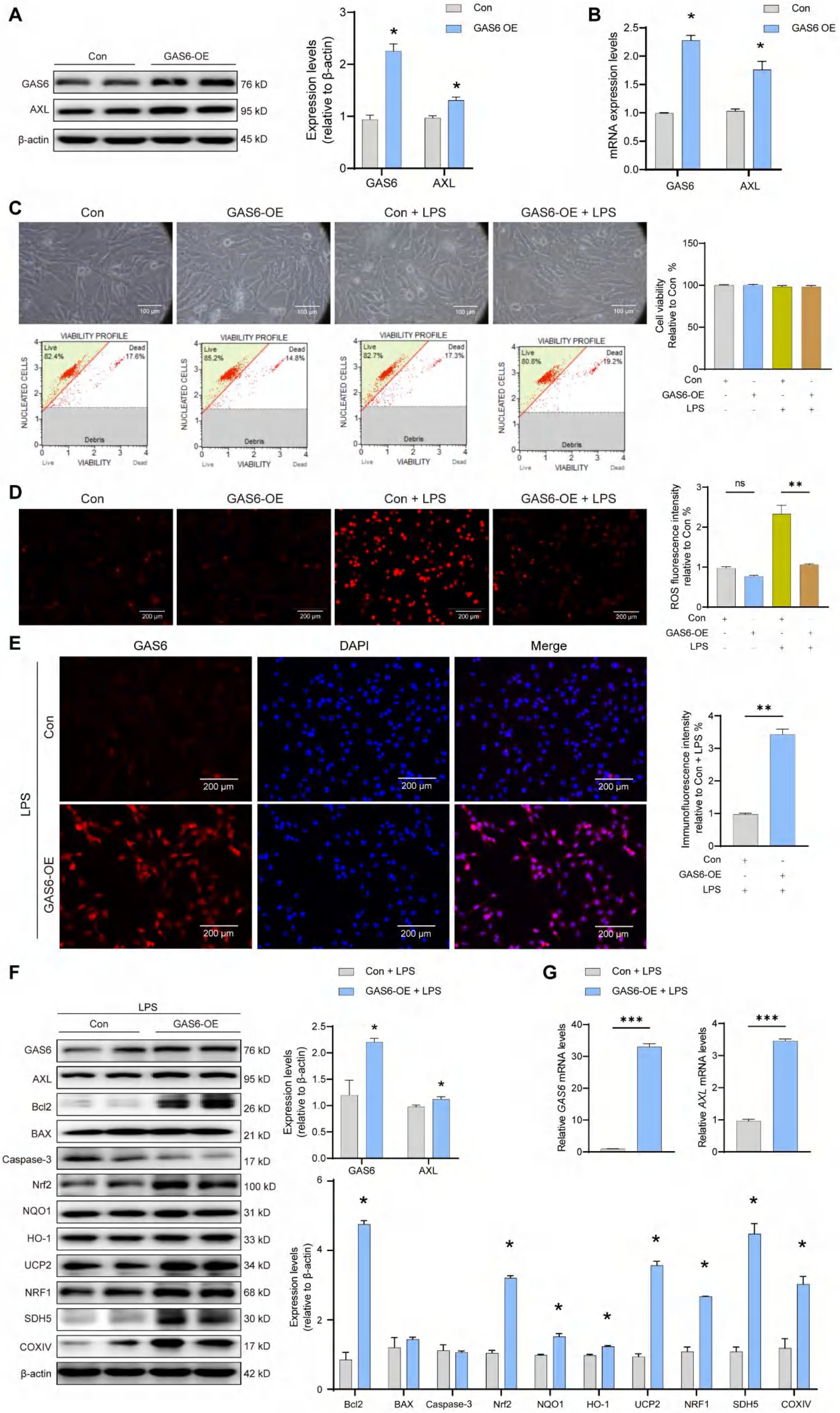
A
Con
GAS6-OE
GAS6
AXL
β-actin
76 kD
95 kD
45 kD
Expression levels (relative to β-actin)
GAS6 OE
B
mRNA expression levels
C
Con
GAS6-OE
Con + LPS
GAS6-OE + LPS
VIABILITY PROFILE
NUCLEATED CELLS
VIABILITY
Live
Dead
Debris
Live 82.4%
Dead 17.6%
Live 85.2%
Dead 14.8%
Live 82.7%
Dead 17.3%
Live 80.8%
Dead 19.2%
Cell viability Relative to Con %
LPS
D
ROS fluorescence intensity relative to Con %
ns
200 µm
100 µm
E
GAS6
DAPI
Merge
Immunofluorescence intensity relative to Con + LPS %
F
Con + LPS
GAS6-OE + LPS
Bcl2
BAX
Caspase-3
Nrf2
NQO1
HO-1
UCP2
NRF1
SDH5
COXIV
26 kD
21 kD
17 kD
100 kD
31 kD
33 kD
34 kD
68 kD
30 kD
42 kD
G
Relative GAS6 mRNA levels
Relative AXL mRNA levels

**Fig. S9 GAS6 overexpression alleviated LPS injury in HL-1 cells. (A)** Representative and pooled Western blot analysis of GAS6 and AXL in HL-1 cells with GAS6-OE; **(B)** qRT-PCR analysis of *GAS6* and *AXL* mRNA in HL-1 cells with GAS6-OE; **(C)** Representative cell morphology images and cell viability of LPS-stimulated HL-1 cells with GAS6-OE; **(D)** Intracellular ROS levels of LPS-stimulated HL-1 cells with GAS6-OE; **(E)** Representative and pooled immunofluorescence photographs for GAS6 (red) and DAPI (blue) in LPS-stimulated HL-1 cells with GAS6-OE; **(F)** Representative and pooled Western blot analysis of GAS6, AXL, Bcl2, BAX, Caspase-3, Nrf2, NQO1, HO-1, UCP2, NRF1, SDH5 and COXIV in LPS-stimulated HL-1 cells with GAS6-OE; and **(G)** qRT-PCR analysis of *GAS6* and *AXL* mRNA in LPS-stimulated HL-1 cells with GAS6-OE. Mean ± SD. n=6 per group, $^{*}P<0.05$, $^{**}P<0.01$, $^{***}P<0.001$ vs. Con (panel A-B) or Con-LPS (panel C-G).

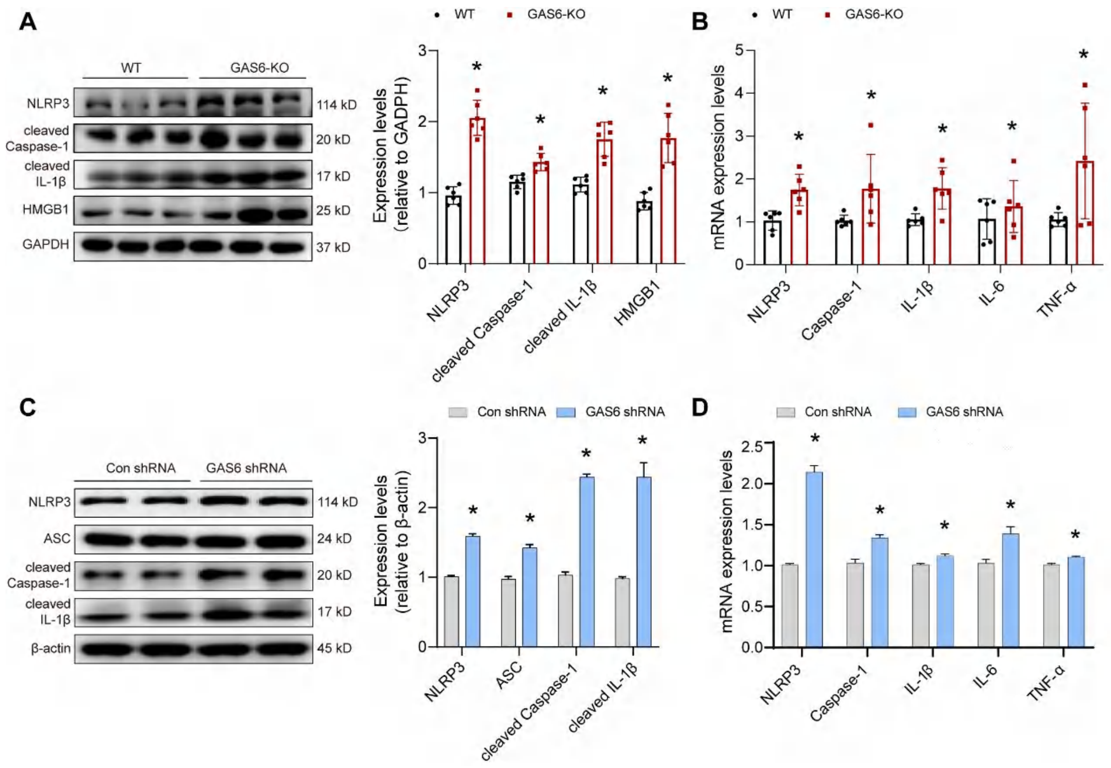


**Fig. S10 Effects of GAS6 deficiency alone on the production of NLRP3-mediated pro-inflammatory mediators *in vivo* or *in vitro*.** **(A)** Representative and pooled Western blot analysis of myocardial NLRP3, cleaved Caspase-1, cleaved IL-1β and HMGB1 in mice with GAS6 deficiency; **(B)** qRT-PCR analysis of myocardial *NLRP3, Caspase-1, IL-1β, IL-6* and *TNF-α* mRNA in mice with GAS6 deficiency; **(C)** Representative and pooled Western blot analysis of NLRP3, ASC, cleaved Caspase-1 and cleaved IL-1β in HL-1 cells with GAS6 shRNA; **(D)** qRT-PCR analysis of *NLRP3, Caspase-1, IL-1β, IL-6* and *TNF-α* mRNA in HL-1 cells with GAS6 shRNA. Mean ± SD. n = 6 per group, $^{*}P<0.05$ vs. WT (panel A-B) or Con shRNA (panel C-D).

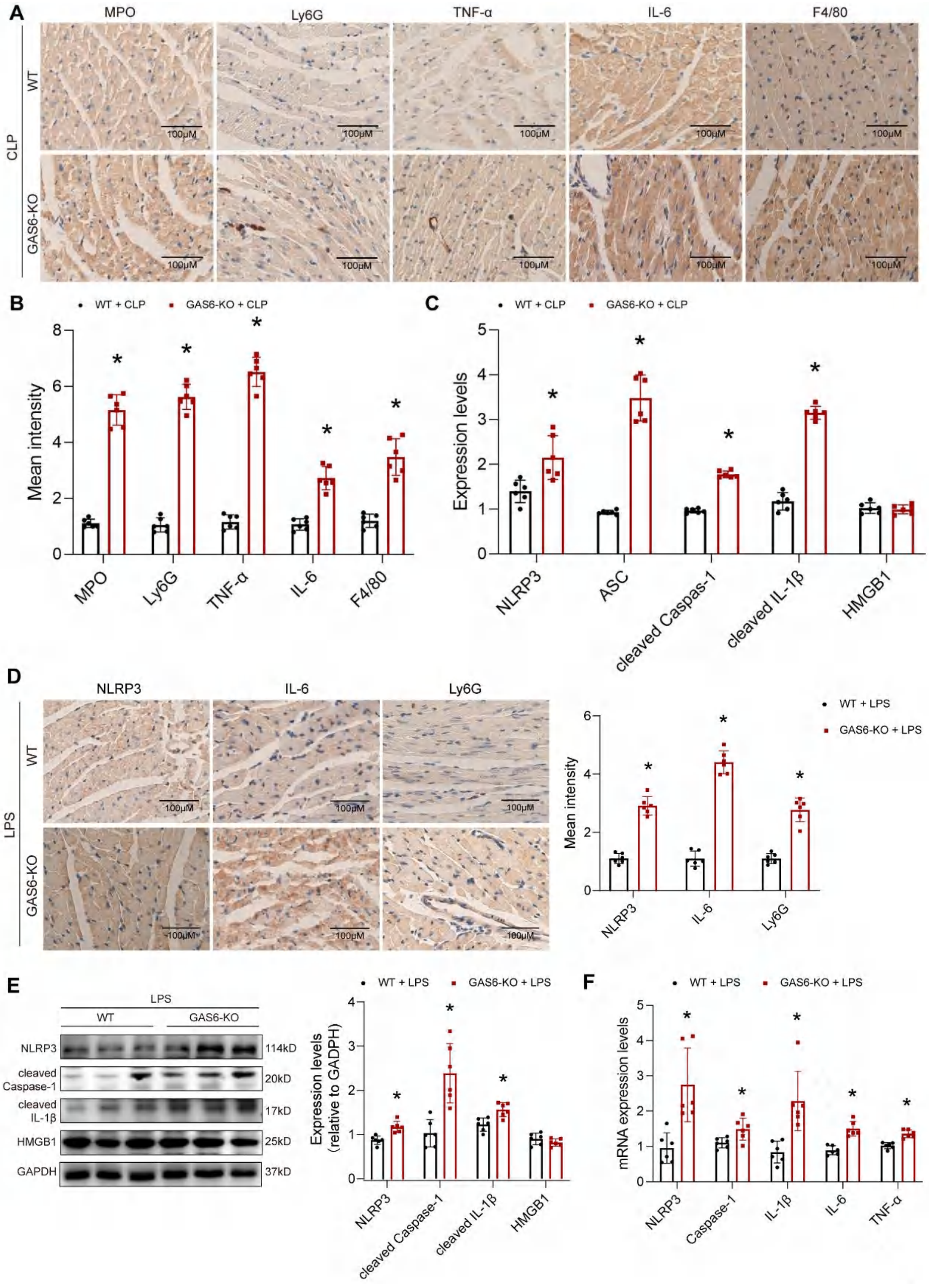


**Fig. S11 Effects of GAS6 knockout on NLRP3-related pro-inflammatory mediators in CLP or LPS mice. (A-B)** Representative and pooled immunohistochemistry staining results of myocardial MPO, Ly6G, TNF-α, IL-6 and F4/80 in mice with GAS6-KO 8 h following CLP; **(C)** Pooled Western blot analysis of myocardial NLRP3, ASC, cleaved Caspase-1, cleaved IL-1β and

HMGB1 in mice with GAS6-KO 8 h after CLP; **(D)** Representative and pooled IHC staining results of myocardial NLRP3, IL-6 and Ly6G in mice with GAS6-KO 8 h following LPS; **(E)** Representative and pooled Western blot analysis of myocardial NLRP3, cleaved Caspase-1, cleaved IL-1β and HMGB1 in mice with GAS6-KO 8 h after LPS injection; and **(F)** qRT-PCR analysis of myocardial NLRP3, Caspase-1, IL-1β, IL-6 and TNF-α mRNA in mice with GAS6-KO 8 h after LPS injection. Mean ± SD. n=6 per group, *$P$<0.05 vs. WT-CLP (panel A-C) or WT-LPS (panel D-F).

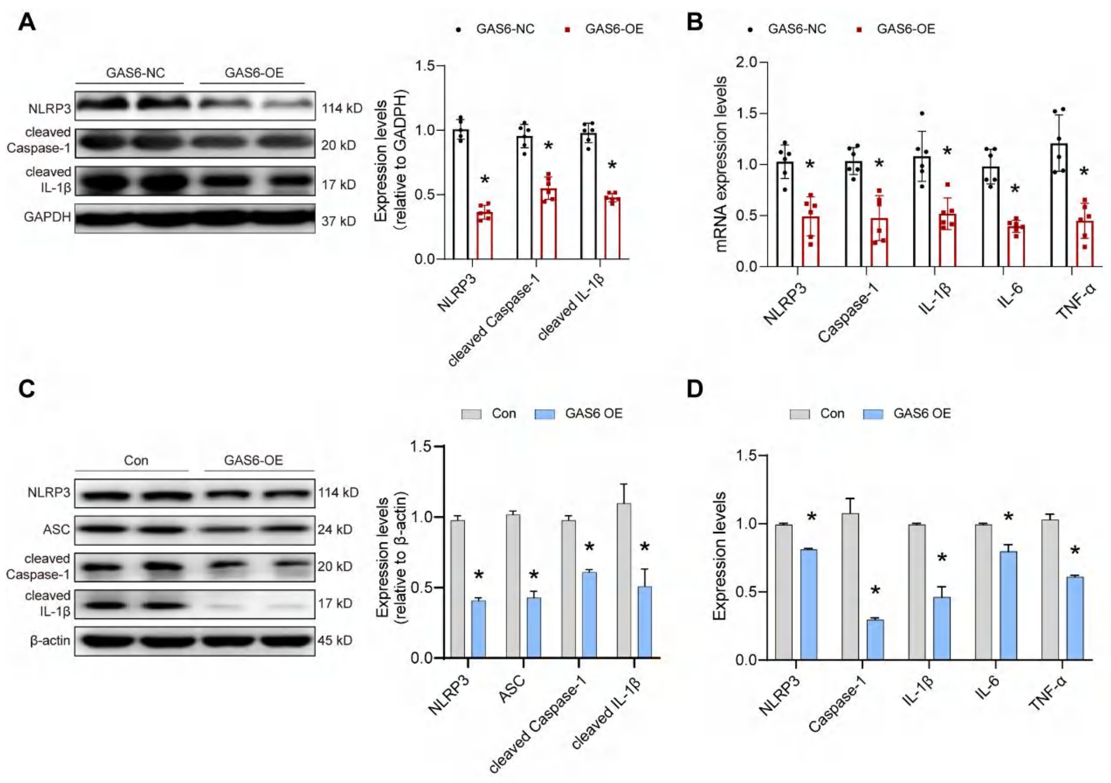


**Fig. S12 Effects of GAS6 overexpression alone on the production of NLRP3-mediated pro-inflammatory mediators *in vivo* or *in vitro*. (A)** Representative and pooled Western blot analysis of myocardial NLRP3, cleaved Caspase-1 and cleaved IL-1β in mice with GAS6-OE; and **(B)** qRT-PCR analysis of myocardial NLRP3, Caspase-1, IL-1β, IL-6 and TNF-α mRNA in mice with GAS6-OE. **(C)** Representative and pooled Western blot analysis of NLRP3, ASC, cleaved Caspase-1 and cleaved IL-1β in HL-1 cells with GAS6-OE; and **(D)** qRT-PCR analysis of *NLRP3, Caspase-1, IL-1β, IL-6* and *TNF-α* mRNA in HL-1 cells with GAS6-OE. Mean ± SD. n = 6 per group, $^{*}P<0.05$ vs. GAS6-NC (panel A-B) or Con (panel C-D).

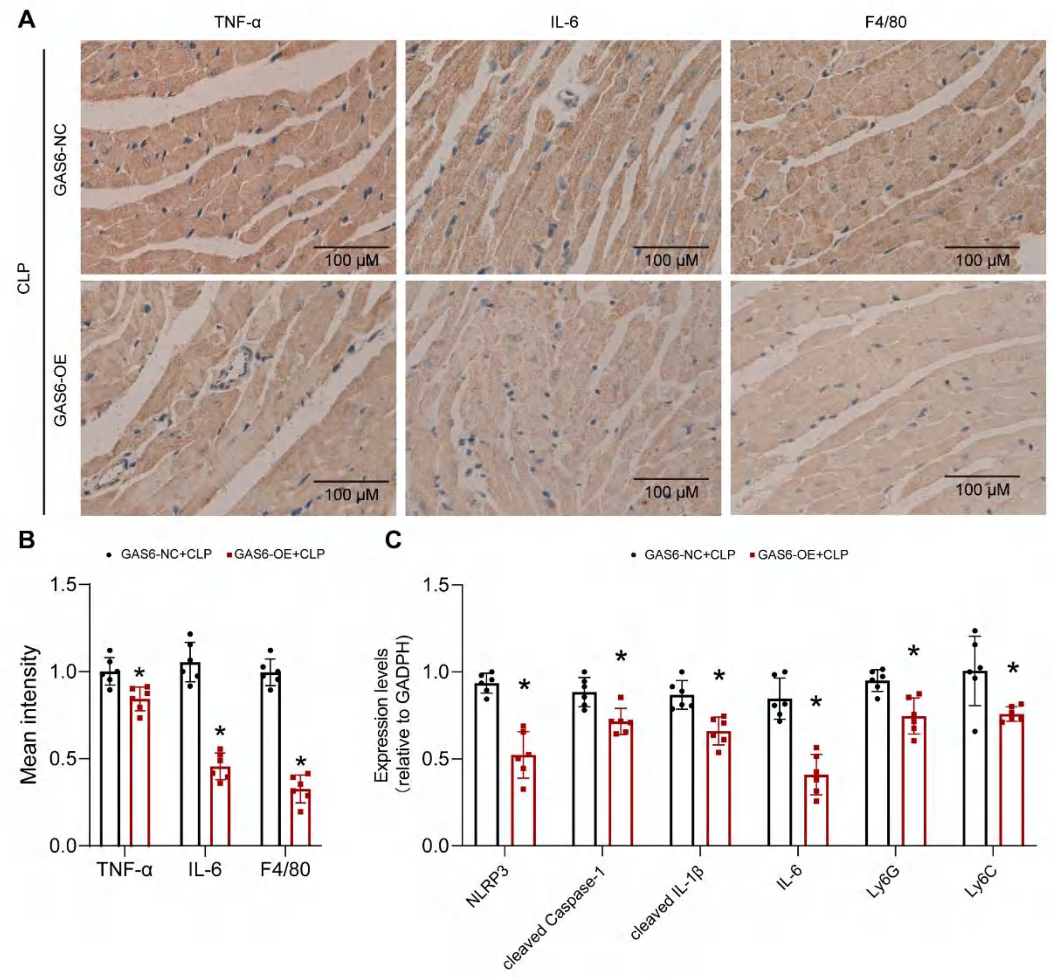


**Fig. S13 GAS6 overexpression down-regulated levels of NLRP3-mediated pro-inflammatory mediators in CLP-injured mice. (A-B)** Representative and pooled IHC staining results of myocardial TNF-α, IL-6 and F4/80 in mice with GAS6-OE 8 h following CLP; **(C)** Pooled Western blot analysis of myocardial NLRP3, cleaved Caspase-1, cleaved IL-1β, IL-6, Ly6G and Ly6C in mice with GAS6-OE 8 h after CLP. Mean ± SD. n=6 per group, $^{*}P<0.05$ vs. GAS6-NC-CLP.

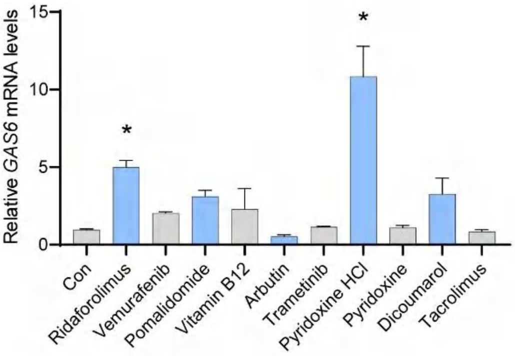
Relative GAS6 mRNA levels
15
10
5
0
*
*
Con
Ridaforolimus
Vemurafenib
Pomalidomide
Vitamin B12
Arbutin
Trametinib
Pyridoxine HCl
Pyridoxine
Dicoumarol
Tacrolimus

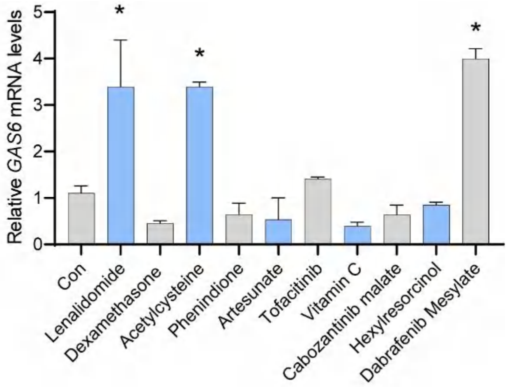
Relative GAS6 mRNA levels
5
4
3
2
1
0
*
*
*
Con
Lenalidomide
Dexamethasone
Acetylcysteine
Phenindione
Artesunate
Tofacitinib
Vitamin C
Cabozantinib malate
Hexylresorcinol
Dabrafenib Mesylate

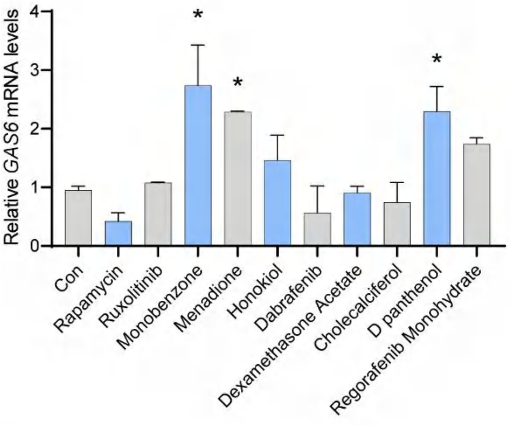
Relative GAS6 mRNA levels
4
3
2
1
0
*
*
*
Con
Rapamycin
Ruxolitinib
Monobenzone
Menadione
Honokiol
Dabrafenib
Dexamethasone Acetate
Cholecalciferol
D panthenol
Regorafenib Monohydrate

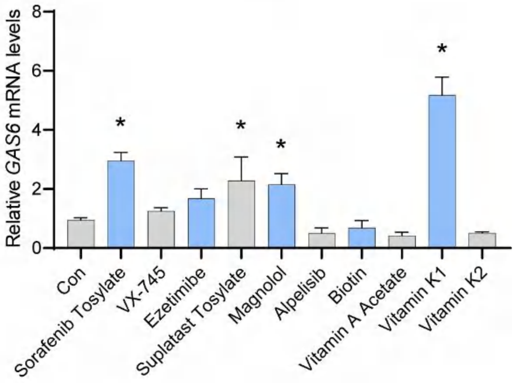
Relative GAS6 mRNA levels
8
6
4
2
0
*
*
*
*
Con
Sorafenib Tosylate
VX-745
Ezetimibe
Suplatast Tosylate
Magnolol
Alpelisib
Biotin
Vitamin A Acetate
Vitamin K1
Vitamin K2

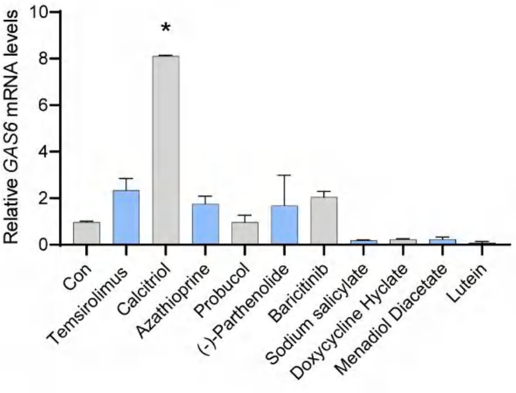
Relative GAS6 mRNA levels
10
8
6
4
2
0
*
Con
Temsirolimus
Calcitriol
Azathioprine
Probucol
(-)-Parthenolide
Baricitinib
Sodium salicylate
Doxycycline Hyclate
Menadiol Diacetate
Lutein

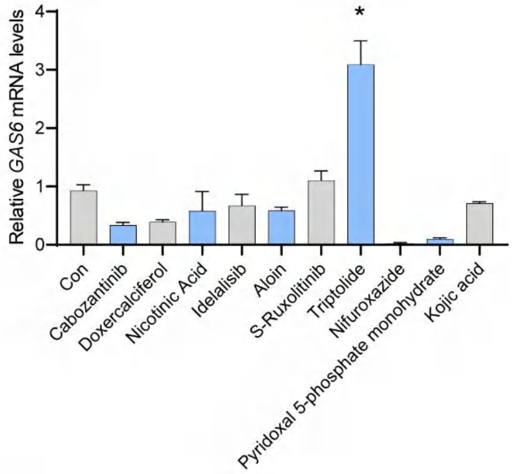
Relative GAS6 mRNA levels
4
3
2
1
0
*
Con
Cabozantinib
Doxercalciferol
Nicotinic Acid
Idelalisib
Aloin
S-Ruxolitinib
Triptolide
Nifuroxazide
Pyridoxal 5-phosphate monohydrate
Kojic acid

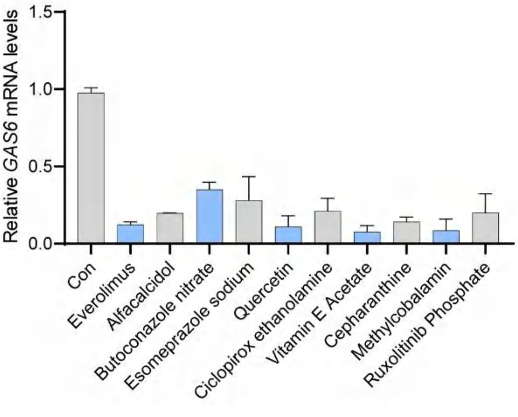
Relative GAS6 mRNA levels
1.5
1.0
0.5
0.0
Con
Everolimus
Alfacalcidol
Butoconazole nitrate
Esomeprazole sodium
Quercetin
Ciclopirox ethanolamine
Vitamin E Acetate
Cepharanthine
Methylcobalamin
Ruxolitinib Phosphate

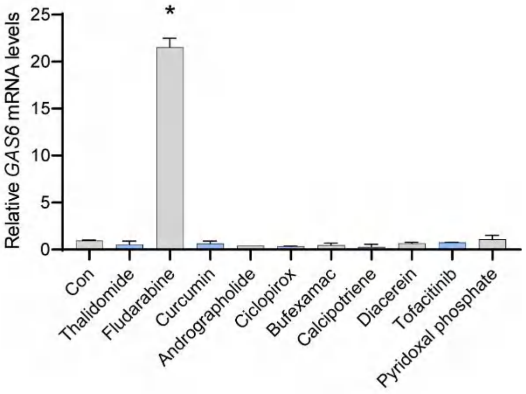
Relative GAS6 mRNA levels
25
20
15
10
5
0
*
Con
Thalidomide
Fludarabine
Curcumin
Andrographolide
Ciclopirox
Bufexamac
Calcipotriene
Diacerein
Tofacitinib
Pyridoxal phosphate

**Fig. S14 Screening of GAS6 pharmacological agonists.** qRT-PCR analysis of *GAS6* mRNA levels in HL-1 cells treated with 123 FDA-approved drugs for 3 h. Mean ± SD. n=6 for each group, $^{*}P<0.05$ vs. Con.

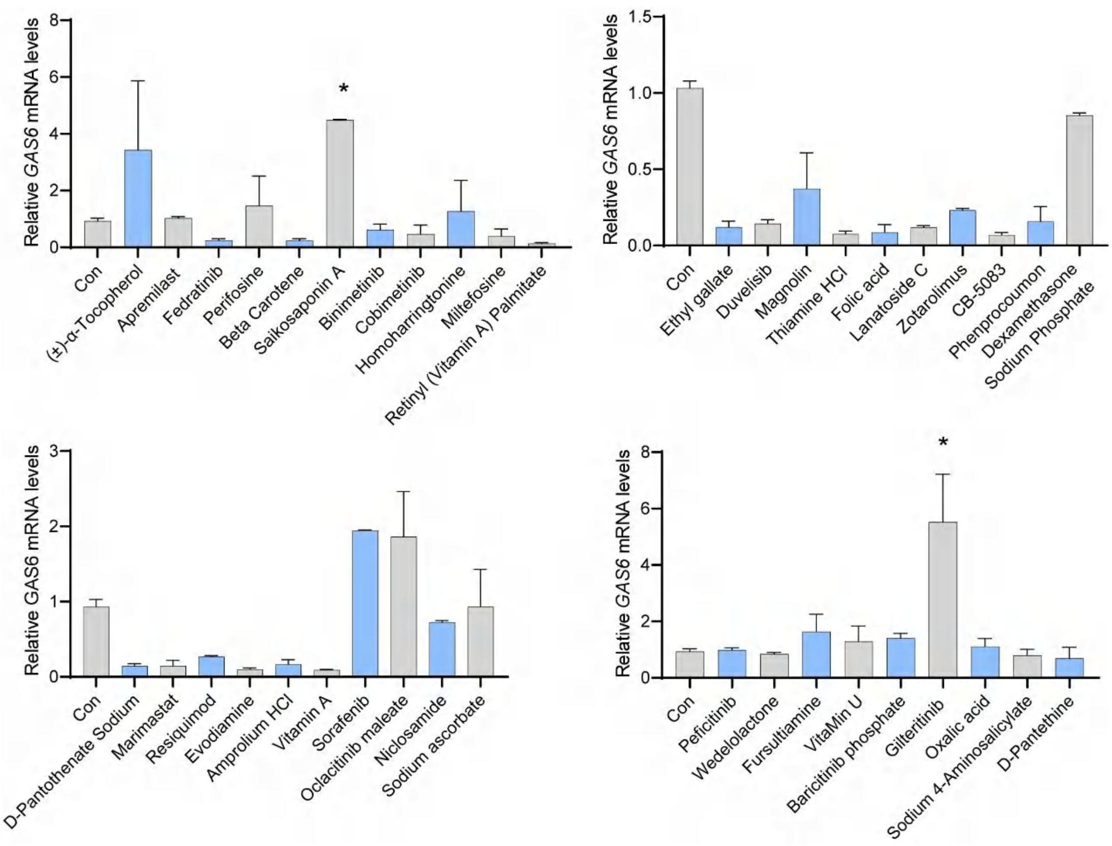


**Fig. S15 Screening of GAS6 pharmacological agonists.** qRT-PCR analysis of *GAS6* mRNA levels in HL-1 cells treated with 123 FDA-approved drugs for 3 h. Mean ± SD. n=6 for each group, $^{*}P<0.05$ vs. Con.

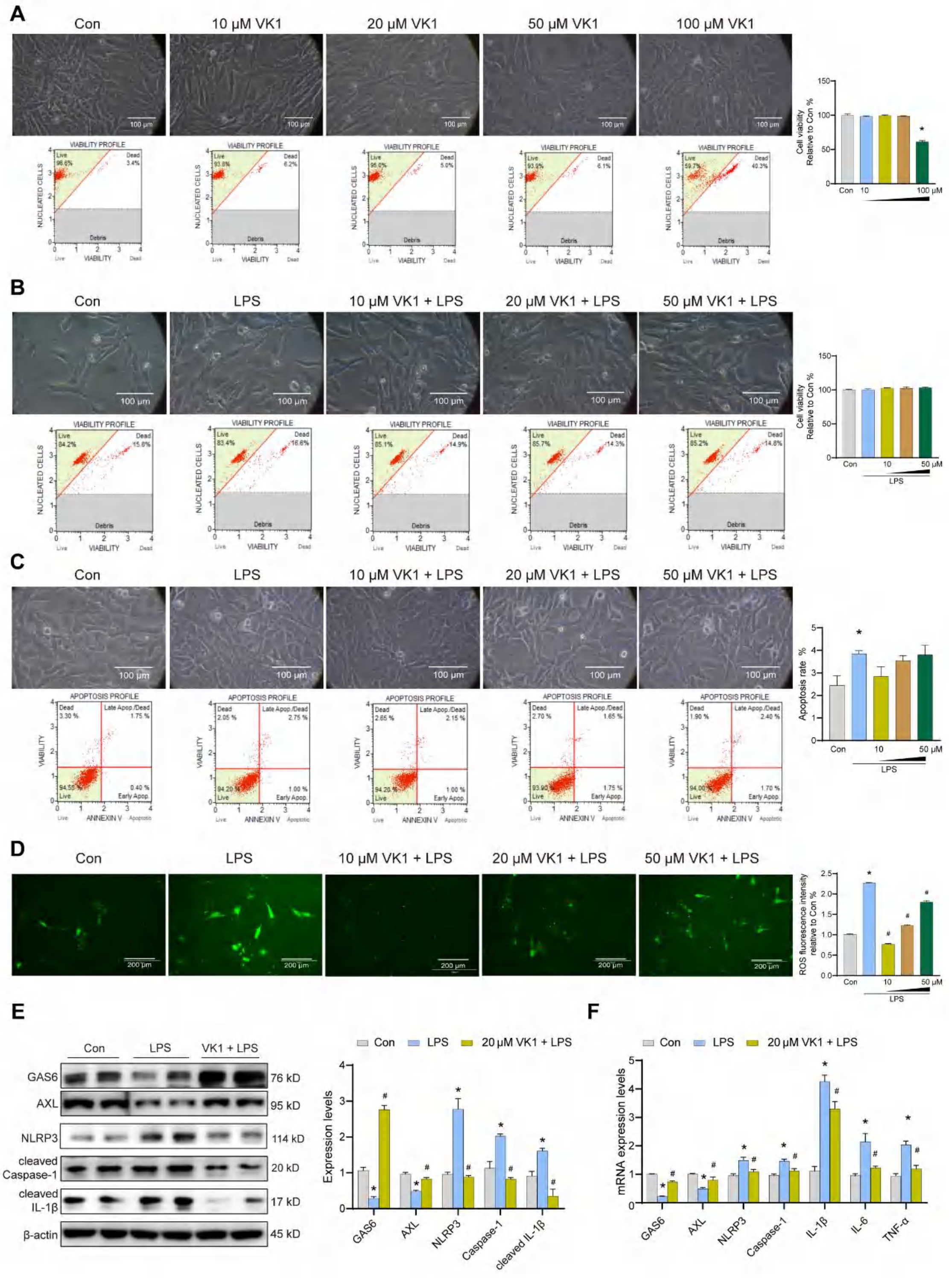


**Fig. S16 VK1 alleviated septic injury in HL-1 cells through a GAS6-dependent manner. (A)** Representative cell morphology images and cell viability of HL-1 cells treated with VK1 for 24 h, n=6; (**B**) Cell viability in HL-1 cells treated with VK1 at different concentrations (10, 20 or 50 μM) for 3 h prior to exposure to LPS, n=6; (**C**) Apoptosis rates of VK1 and LPS-treated HL-1 cells; (**D**)

Intracellular ROS levels of VK1 and LPS-treated HL-1 cells; (**E**) Representative and pooled Western blot analysis of GAS6, AXL, NLRP3, cleaved Caspase-1 and cleaved IL-1β in VK1 and LPS-treated HL-1 cells; and **(F)** qRT-PCR analysis of *GAS6, AXL, NLRP3, Caspase-1, IL-1β, IL-6* and *TNF-α* mRNA in VK1 and LPS-treated cells; n=6. Mean ± SD. **P*<0.05 vs. Con (panel A). or vs. LPS (panel B-F).

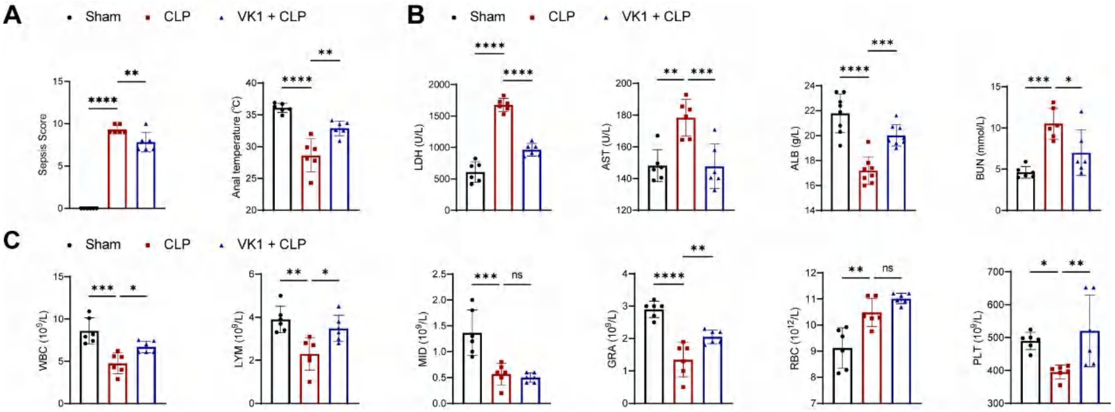


**Fig. S17 Effects of VK1 on the sepsis score, anal temperature, and systematical status of CLP mice. (A)** Sepsis score and anal temperature in mice subject to VK1 pretreatment and CLP operation **(B)** Blood biochemical parameters in mice subject to VK1 pretreatment and CLP operation; **(C)** Blood routine parameters in mice subject to VK1 pretreatment and CLP operation. Mean ± SD. $^{*}P$<0.05 vs. Sham.

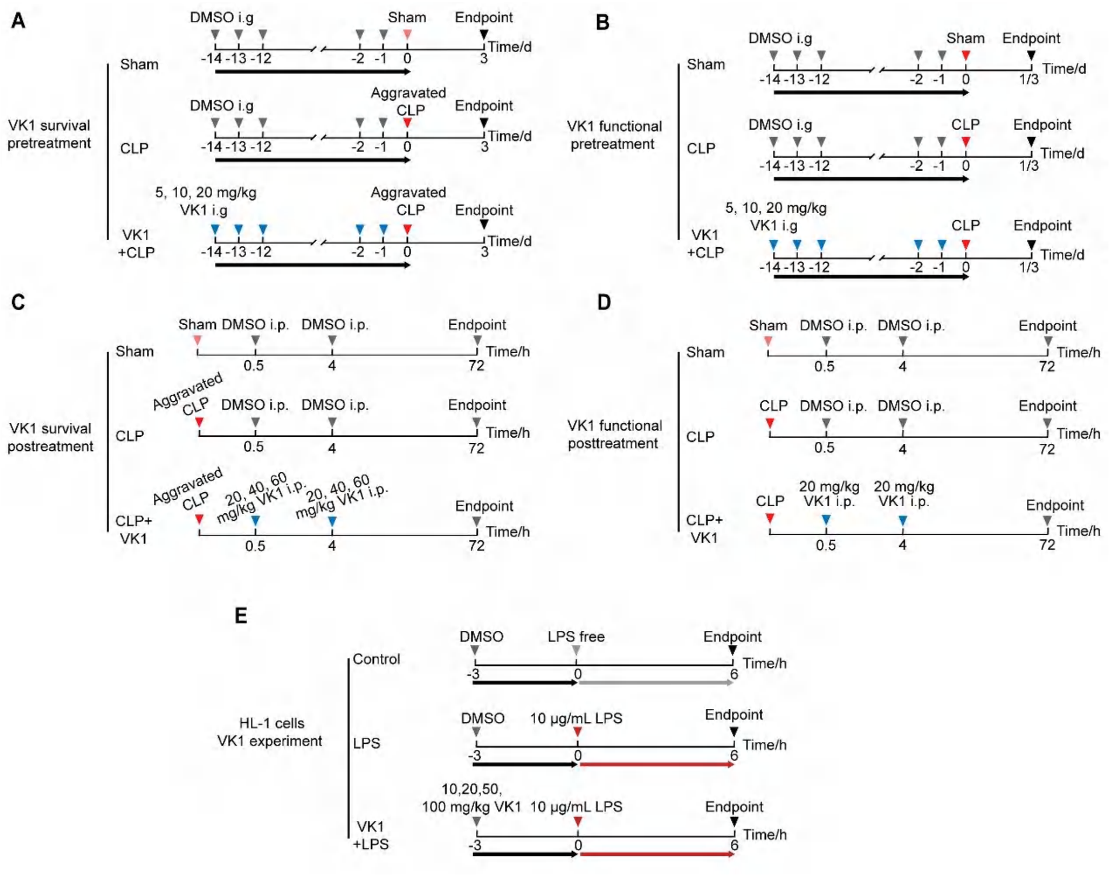


**Fig. S18 Experiment timeline. (A)** Experimental design of survival rate in CLP mice pretreated with VK1; (**B**) Experimental design of functional analysis in VK1-pretreated CLP mice; **(C)** Experimental design of survival rate in CLP mice post-treated with VK1; (**D**) Experimental design of functional analysis in mice with VK1 post-treated; and (**E**) Experimental design of VK1-treated HL-1 cells.
